# Phylogenetic affiliation of endophytic actinobacteria associated with selected orchid species and their role in growth promotion and suppression of phytopathogens

**DOI:** 10.3389/fpls.2022.1058867

**Published:** 2022-12-07

**Authors:** Juri Saikia, Rajkumari Mazumdar, Debajit Thakur

**Affiliations:** ^1^ Microbial Biotechnology Laboratory, Life Sciences Division, Institute of Advanced Study in Science and Technology (IASST), Guwahati, India; ^2^ Department of Biotechnology, Gauhati University, Guwahati, India; ^3^ Department of Molecular Biology & Biotechnology, Cotton University, Guwahati, India

**Keywords:** endophytic actinobacteria, orchid, PGP, antifungal, IAA, SEM, GC-MS, chili plant

## Abstract

Endophytic actinobacteria aid in plant development and disease resistance by boosting nutrient uptake or producing secondary metabolites. For the first time, we investigated the culturable endophytic actinobacteria associated with ten epiphytic orchid species of Assam, India. 51 morphologically distinct actinobacteria were recovered from surface sterilized roots and leaves of orchids and characterized based on different PGP and antifungal traits. According to the 16S rRNA gene sequence, these isolates were divided into six families and eight genera, where *Streptomyces* was most abundant (n=29, 56.86%), followed by *Actinomadura*, *Nocardia*, *Nocardiopsis*, *Nocardioides*, *Pseudonocardia*, *Microbacterium*, and *Mycolicibacterium*. Regarding PGP characteristics, 25 (49.01%) isolates demonstrated phosphate solubilization in the range of 61.1±4.4 - 289.7±11.9 µg/ml, whereas 27 (52.94%) isolates biosynthesized IAA in the range of 4.0 ± 0.08 - 43.8 ± 0.2 µg/ml, and 35 (68.62%) isolates generated ammonia in the range of 0.9 ± 0.1 - 5.9 ± 0.2 µmol/ml. These isolates also produced extracellular enzymes, viz. protease (43.13%), cellulase (23.52%), pectinase (21.56%), ACC deaminase (27.45%), and chitinase (37.25%). Out of 51 isolates, 27 (52.94%) showed antagonism against at least one test phytopathogen. In molecular screening, most isolates with antifungal and chitinase producing traits revealed the presence of 18 family chitinase genes. Two actinobacterial endophytes, *Streptomyces* sp. VCLA3 and *Streptomyces* sp. RVRA7 were ranked as the best strains based on PGP and antifungal activity on bonitur scale. GC-MS examination of ethyl acetate extract of these potent strains displayed antimicrobial compound phenol, 2,4-bis-(1,1-dimethylethyl) as the major metabolite along with other antifungal and plant growth beneficial bioactive chemicals. SEM analysis of fungal pathogen *F. oxysporum* (MTCC 4633) affected by *Streptomyces* sp. VCLA3 revealed significant destruction in the spore structure. An *in vivo* plant growth promotion experiment with VCLA3 and RVRA7 on chili plants exhibited statistically significant (p<0.05) improvements in all of the evaluated vegetative parameters compared to the control. Our research thus gives insight into the diversity, composition, and functional significance of endophytic actinobacteria associated with orchids. This research demonstrates that isolates with multiple plant development and broad-spectrum antifungal properties are beneficial for plant growth. They may provide a viable alternative to chemical fertilizers and pesticides and a sustainable solution for chemical inputs in agriculture.

## Introduction

Plants provide a multitude of niches for the growth and proliferation of diverse microorganisms in the rhizosphere, phyllosphere, and endosphere to form complex co-associations, which is beneficial for promoting the productivity and health of the plant in both natural and cultivated environments (Trivedi et al., 2020). On the external surface of the plants, various microbial interactions take place predominantly in the root zone (rhizosphere) and on aerial tissues, notably in the leaves (phyllosphere) (Velmourougane et al., 2017). Some fungi and bacteria from the rhizosphere and phyllosphere can penetrate the plant’s interior known as endophytes, colonize intercellular spaces and vascular tissues and establish a non-harmful relationship with their host without causing disease or significant morphological changes. Endophytes are ubiquitous and are present in all plant organs and species (Zhang et al., 2019). They are recognized as plant growth-promoting (PGPB) bacteria that flourish within plants and boost plant development under normal and stress conditions either directly by raising nutrient absorption and affecting growth and stress-related phytohormones or indirectly by employing antibiotics, hydrolytic enzymes, limiting nutrients, and priming plant defenses to combat pests and pathogens (Afzal et al., 2019). Plants are endophytically colonized by a diverse group of bacteria from different taxon. However, Actinobacteria has been discovered to be a prevalent phylum among endophytic bacteria in most plants (Matsumoto and Takahashi, 2017; Cao et al., 2020). Actinobacteria are gram-positive, aerobic bacteria with a high guanine-cytosine content (57–75%) in their genome. They are free-living in soil and can be found in the rhizosphere, phyllosphere, and endosphere. They serve a significant ecological function in cycling soil nutrients and are widespread, forming a stable and persistent population in many habitats (Narsing Rao and Li, 2022). Actinobacteria are well recognized for producing a variety of bioactive secondary metabolites that are useful in both the pharmaceutical and agricultural industries (Salwan and Sharma, 2020). Numerous plant species have been studied for endophytic actinobacteria, including rice (Gangwar et al., 2012), wheat (Elisandra et al., 2016), legumes (Xu et al., 2022), tomato (López et al., 2020), medicinal plants (Oberhofer et al., 2019; Mohamad et al., 2022), cereal crops (Patel et al., 2018), chinaberry (Zhao et al., 2018a), cucumber (Cao et al., 2020), tea (Borah et al., 2020), Chinese licorice (Zhao et al., 2018b), etc. Endophytic actinobacteria improve host plant growth through phosphate solubilization, biological nitrogen fixation, and synthesis of growth-exhilarating phytohormones like indole-3-acetic acid (IAA). They shield the host plants from pathogens and illness by producing bioactive antifungal chemicals, polymer hydrolyzing enzymes such as siderophores, cellulase, protease, and chitinase, which aid in fungal hyphal lysis, cause fungal cell wall disintegration, and inhibit spore germination (Liu et al., 2017; Ahmad et al., 2020). Furthermore, they assist the plant in coping with adverse climatic conditions by reducing the stress hormone ethylene *via* the aminocyclopropane carboxylate deaminase (ACCD) enzyme, which converts ACC to ethylene (Yoolon et al., 2019).

Chemical fertilizers and pesticides have been used on a large scale to increase agricultural yield to meet the growing food demand. These chemical-dependent agricultural methods significantly threaten the environment, human health, crop quality, and soil fertility. There is a pressing need of the hour to lower the use of these agrochemicals and encourage sustainable farming practices. Scientists have discovered that microorganisms with multifunctional activity were able to minimize the use of these chemicals by generating a wide range of bioactive substances, and enzymes that promote plant development and confer protection (Janardhan et al., 2014; Mitra et al., 2022). Endophytic bacteria, especially actinobacteria, have a strong potential for improving plant growth as bioinoculant or biocontrol weapons for combating phytopathogens. Endophytic actinobacterial diversity is less explored than other groups of microbes. Their diversity varies tremendously depending on the habitat of the host plant as well as between species and genera (Singh and Dubey, 2018). Taking this into account, investigating different plant species with varying habitats may boost the possibilities of finding functionally active endophytic actinobacteria for agricultural application. As a result, various studies over the last few decades have concentrated on the structure and functions of the endophytic actinobacteria in order to establish a link between plant fitness and agricultural productivity.

Orchidaceae is one of the most prominent plant families, with around 25,000 species (Christenhusz and Byng, 2016). Though orchids have therapeutic value, they are primarily grown for their lovely blossoms and are well known for their economic significance (Pant and Raskoti, 2013). Orchids are epiphytic (growing on plants), terrestrial (growing on soil), lithophytic (growing on rocks), or a combination of the three in some situations. Orchids’ native environments support the development of advantageous symbiotic interactions with various microorganisms. Although generated in thousands or millions of seeds per capsule, orchid seeds are minute in size (0.05-6 mm) and lack endosperm. Symbiotic association with suitable mycorrhizal fungi is critical for orchid seeds to germinate. Epiphytic orchids depend on mycorrhizas due to poor nutrient and water availability in aerial habitats (Suárez and KottKe, 2016). Therefore, orchids inevitably depend on mycorrhizal fungi for their growth and survival during all or some part of their life cycle (McCormick et al., 2018). The majority of work in the published literature on orchid microbiological research has focused on orchid endophytic or mycorrhizal fungal communities due to their relevance in the survival of these endangered plants. However, just a few research have looked into endophytic bacteria that focused on their capacity to improve plant growth and disease control. In orchids, bacterial endophytes have been studied in commercially valuable species, and various isolates have been discovered to improve seed germination, plant development, and the hardening of micro-propagated plants (Tsavkelova et al., 2007a; Tsavkelova et al., 2007b; Faria et al., 2013; Gontijo et al., 2018; Wang et al., 2019; Herrera et al., 2020; Shah et al., 2021; Bhatti and Thakur, 2022). From the literature survey, we have not found any report regarding the diversity and functional characterization of endophytic actinobacteria associated with orchids of North-East India. Though few studies on orchid endophytic diversity through an uncultured approach have shown the presence of actinobacteria as a major phylum (Wang et al., 2019; Alibrandi et al., 2020), there is no document about culturable endophytic actinobacteria associated with orchids. Thus, the present study is the first attempt to explore the endophytic actinobacterial community prevalent in different wild-grown epiphytic orchid species such as *Dendrobium nobile, Dendrobium chrysotoxum, Dendrobium moschatum, Dendrobium densiflorum, Dendrobium fugax, Rhynochostylis retusa, Vanda corulea, Renanthera imschootiana, Micropera obtusa, Cymbidium eburneum* of Assam, India through the phylogenetic, molecular and functional approach. Our study aims to estimate the composition and functional diversity of associative endophytic actinobacteria from healthy roots and leaves of the selected orchid species to fill the knowledge gap about epiphytic orchid associated endophytic actinobacteria.

## Materials and methods

### Sample collection and surface sterilization

The plant species selected for the study were *Dendrobium nobile*, *Dendrobium chrysotoxum*, *Dendrobium moschatum*, *Dendrobium densiflorum*, *Dendrobium fugax*, *Rhynochostylis retusa*, *Vanda corulea*, *Renanthera imschootiana*, *Micropera obtusa*, *Cymbidium eburneum*. Root and leaf tissues of healthy orchid plants were collected from the orchid society of eastern Himalaya, regional orchid germplasm conservation and propagation center, Tinsukia- 786156, Assam, India (**Supplementary Table 1**). All ten orchid species were identified and validated by the conservation center. Plant samples (roots and leaves) were sealed with wax and brought to the laboratory in an ice box. Orchid root and leaf tissues were surface sterilized by following several treatments. First, tissue samples were rinsed with tween 20 under tap water to remove any adherent debris. Then the samples were cut into small pieces (1cm^2^). Root tissues were surface sterilized step by step, immersing them in ethanol (70%) for one minute, sodium hypochlorite (2.5%) for ten minutes, and then rinsed with autoclaved distilled water (Schulz et al., 1993).

The same treatment was used on leaf samples, including immersing them in ethanol (70%) for thirty seconds, sodium hypochlorite (1.5%) for five minutes, multiple washing with autoclaved distilled water, and drying them in a laminar airflow chamber. Surface sterilized root and leaf tissues were crushed using a mortar pestle and serially diluted up to 10^-4^ with normal saline (0.8%).100 μl aliquot of each dilution was spread uniformly onto different selective isolation media, viz. starch casein agar, streptomyces agar, actinomycetes isolation agar, and international streptomyces project (ISP) medium 2, ISP3, ISP4, and ISP7, (HiMedia, India). Rifampicin (2.5 µg/mL) and amphotericin B (75 µg/mL) were used in all the media preparation to prevent the growth of endophytic bacteria and fungi. The plates were incubated at 28°C for 7 to 21 days and regularly observed for the appearance of dry and rough actinobacterial colonies. The colonies were picked based on their phenotypic morphology, and pure culture was attained by repeatedly re-culturing on GLM agar medium. 20% w/v glycerol stock was made and kept at -80°C for later use.

### Validation of surface sterilization

To isolate only the endophytes, the surface sterilization procedure was verified (Schulz et al., 1993). For this, 0.1 ml water from the sterilization process’s final wash was spread on the GLM agar plate and cultured for up to 7 days at 28°C. Without any microbial growth on the plates, the sterilizing process was deemed effective.

### Morphological characterization

Following the International Streptomyces Project, endophytic actinobacterial isolates were identified and characterized by evaluating cultural and morphological features such as pigment synthesis, substrate, and aerial mycelia color (Shirling and Gottlieb, 1966).

### Molecular characterization and phylogenetic analysis

#### Extraction of genomic DNA

For isolation of genomic DNA endophytic actinobacterial isolates were inoculated from freshly grown culture plates onto 200 ml GLM broth (yeast extract, 3g; malt extract, 3g; peptone, 5g; starch/glycerol, 10g; agar, 20g; distilled water, 1000mL; pH 7.4), and cultured for 5 days on a rotary shaker (150 rpm) at 28°C. Genomic DNA was extracted from the isolates according to the manufacturer’s instructions using the Nucleopore Fungus/Bacteria kit (Genetix, India) and analyzed in a 0.8% agarose gel. The purity of the extracted DNA was tested using a Nanodrop spectrophotometer (Thermo Scientific, USA) at 260/280 nm absorbance ratio, and the total DNA was expressed in ng ml^-l^.

#### 16S rRNA gene amplification and sequencing

Universal primer pairs 27F (5′-AGAGTTTGATCMTGGCTCAG-3′) and 1492R (5′-TACGGYTACCTTGTTACGACTT-3′) (Weisburg et al., 1991) were used for the amplification of 16 S rRNA gene. The reaction was performed on a master cycler nexus PCR system (Eppendorf, Germany) in a 50μl reaction mixture with 1.0μl template DNA (10 ng), 0.2μl of each primer (10μM), 1X Taq DNA polymerase buffer (2.5 U), 1.5 M MgCl_2_, and 0.2mM of each dNTP, 1 U Taq DNA polymerase enzyme (TaKaRa Bio Inc., Japan). The PCR was run under the following conditions: 94°C for 5 minutes-initial denaturation; followed by 35 cycles of denaturation at 94°C for 30 s, 52°C for 30 s, 72°C for 1 minute, 72°C for 1 minute -annealing, and 72°C for 7 minutes-final extension. The PCR result was examined using a 1.8% agarose gel dyed with EtBr (10 g/mL), and the gel electrophoresis was run at 70 V for 45 minutes. PCR bands were then visualized and imaged in a BioRad Gel Doc XR+ system (Hercules, CA, USA).

For molecular characterization of the actinobacterial isolates, the 16S rRNA gene was sequenced commercially by 1st BASE DNA sequence services, Malaysia. To prepare the sequence for analysis, it was first edited using the Codon Code Aligner program and then searched for homology with sequences of other organisms from the NCBI (National Center for Biotechnology Information) with BLAST (Basic Local Alignment Search Tool) search engine and the EzTaxon database (Chun et al., 2007). In the GenBank database, the partial 16S rRNA gene sequences were deposited, and accession numbers were obtained against each submitted sequence.

#### Phylogenetic analysis

To study the evolutionary relationship among the actinobacterial isolates, the 16S rRNA gene sequences were aligned with CLUSTAL W, and a phylogenetic tree was created based on the neighbor-joining method in MEGA X software (windows x64 version) with 1000 bootstraps value (Kumar et al., 2016) and Tamura -Nei distance model (Tamura and Nei, 1993).

### Detection of plant growth promoting traits *in vitro*

#### Indole-3-acetic acid production

The modified Gordon and Weber method (1951) was used for qualitative and quantitative estimation of IAA biosynthesis. Endophytic actinobacterial isolates were inoculated onto GLM broth, added with 0.2% l- tryptophan, and incubated at 28°C for 7 days in a shaking incubator (at 125 rpm). The culture was centrifuged, then100 ml of the supernatant was mixed with 200 ml of salkowski’s reagent (35% HClO_4_ and 10 mM FeCl_3_) and incubated at room temperature for 15 minutes in the dark. The development of pink to red color indicates a positive result, and the absorbance was recorded in a multimode reader at 530 nm. A standard curve of commercial IAA with a known concentration was used to evaluate the quantitative value of IAA produced by the actinobacterial isolates and expressed in µg/ml (Sigma-Aldrich, US).

#### Phosphate solubilization

Phosphate solubilization was estimated with a modified method by Katznelson and Bose (2010). Actinobacterial broth culture was spotted on pikovskaya’s agar plate and incubated for 7 days at 28°C. Positive actinobacterial isolates were identified by observing a clean halo zone around the colonies and were further used to quantify phosphate solubilization. Pikovskaya’s broth was used to culture the positive actinobacterial isolates for 7 days at 28°C in a shaking incubator (125 rpm). The culture broth was then centrifuged for 10 minutes at 7,000 g. An ammonium molybdate reagent comprising 10% ascorbic acid and 2.5% ammonium molybdate in 1N H_2_SO_4_ was mixed with the supernatant in a 1:1 ratio (Fiske and Subbarow, 1925). Absorbance was recorded at 650 nm in a multimode reader. The quantitative phosphate solubilization value was calculated with a standard curve of KH_2_PO_4_ and expressed in µg/ml.

#### Ammonia production

A method developed by Cappuccino and Sherman, 2014 was employed to estimate ammonia production. Actinobacterial broth cultures grown on peptone water were centrifuged for 10 minutes at 10000 rpm. 50 µl of Nessler’s reagent was added to 1 ml of each culture supernatant. A positive result for ammonia production was validated by the formation of dark yellow to brown color. At 530 nm, absorbance was recorded, and ammonia production was calculated using an ammonium sulfate standard curve and expressed in µmol/ml.

#### Siderophore production

Chrome Azurol S (CAS) agar plate assay was used to identify siderophore activity (Schwyn and Neilands, 1987). Spot inoculations of actinobacterial broth cultures were made on CAS (Chrome Azurol S) agar plates and incubated at 28°C for 7 days. The colonies with a yellow-orange clear zone were recognized as siderophore producers. The quantitative value of siderophore production was determined using a CAS-shuttle assay. After centrifuging the actinobacterial broth cultures at 10,000 rpm for 10 minutes, the culture supernatant was inoculated onto 96-well plates with CAS reagent in a 1:1 ratio. Pure GLM broth and CAS reagent in a 1:1 ratio was taken as a reference, and absorbance was measured at 630 nm. The amount of siderophore produced was calculated as follows (Pal and Gokarn, 2010).

Siderophore (%) = Ar − As/Ar × 100

Where, Ar = absorbance of the reference solution and As = absorbance of the sample at 630 nm.

### ACC deaminase activity

Aminocyclopropane-1-carboxylate (ACC) deaminase activity was detected using DF (Dworkin and Foster, 1958) salt medium. To enable the isolates to utilize 3 mM ACC (Onofre-Lemus et al., 2009) as their only nitrogen source, it was filter sterilized and spread on the DF media. Spot inoculations of actinobacterial isolates were made and incubated at 28°C for 7 days. After 7 days, actinobacterial growth was observed (Naik et al., 2008).

### Extracellular enzyme production

#### Protease

Skim milk agar was utilized to identify the proteolytic activity of the endophytic actinobacterial isolates. The composition of skim milk agar is (per liter) yeast extract -5g, glucose -1g, pancreatic digest of casein -5g, skim milk powder -28g, and agar -15g. Actinobacterial broth culture was spotted onto skim milk agar plates and incubated at 28°C for 7 days. Isolates that degraded the skim milk on the SMA plate and left a clean halo around their colonies were identified as protease enzyme producers (Kazanas, 1968).

#### Chitinase

To detect chitinase enzyme production, actinobacterial isolates were spot inoculated onto a colloidal chitin agar plate which contains (per liter) colloidal chitin (15g), yeast extract (0.5g), (NH4)_2_ SO_4_ (1g), MgSO_4_ 6H_2_O (0.3g), KH_2_PO_4_ (1.36g), agar (15g). A distinct halo zone encircling the colonies indicated a positive result (Skujins et al., 1965).

#### Cellulase

M9 minimal salt CMC medium was used to identify cellulase activity of the endophytic actinobacterial isolates. The composition of M9 CMC media is KH_2_PO_4_ (15g), Na_2_HPO_4_ (33.9g), NaCl (2.5g), NH_4_Cl (5g), agar (15g), yeast extract (1.2g), and carboxy methyl cellulose (10g). Actinobacterial broth cultures were spot-inoculated and kept at 28°C for 7 days. The plates were submerged in 1 ml of gram’s iodine and then destained with 9% (w/v) sodium chloride. The development of a distinct, clear zone surrounding the colonies served as a marker for positive isolates (Kasana et al., 2008).

#### Pectinase activity

M9 minimal salt medium amended with pectin (1% w/v) was used to detect pectinase activity. Actinobacterial isolates were initially spot-inoculated on an M9 minimal salt medium for 7 days at 28°C. A clear zone enclosing the actinobacterial colonies when the plates were submerged in congo red (0.12%) confirmed a positive result (Kasana et al., 2008).

### Detection of antifungal activity

#### Test fungal pathogens

Ten plant pathogenic fungi were used to detect the antifungal activity of all 51 endophytic actinobacterial isolates. The test pathogens selected were *Poria hypobrunnea* (ITCC 4141), *Colletotrichum capsici* (MTCC 8473)*, Fusarium oxysporum* (MTCC 284)*, Rhizoctonia solani* (MTCC 4633), *Phellinus lamaensis* (ITCC 292), *Fusarium solani* (MTCC 365), *Curvularia eragrostidis* (MTCC 8198)*, Glomerulla cingulata* (MTCC 2033), *Pestalotiopsis theae* (ITCC 6599), and *Nigrospora sphaerica* (KJ767520) (Dutta et al., 2015).

#### Antifungal activity

For screening of antifungal activity dual culture spot inoculation method was employed. The actinobacterial broth cultures were first set at 1×10^8^ CFU ml^-1^ and equidistantly spotted onto PDA plates. The pathogen’s fungal agar plug (5 mm) was kept in the center of the plate. A plate with just a fungal disc from the test pathogen was used as a control. The plates were incubated for 14 days at 28°C, and three replicates were taken for each experiment. The diameter of the fungal mycelia on the test and control plates was measured to determine the antagonistic effect, and the inhibition percentage was calculated as follows: % Inhibition= C-T/C×100, where C and T are the diameter of fungal mycelia on the control and test plate, respectively.

The disc diffusion method was also used for the screening of antifungal activity of the actinobacterial isolates. The antifungal bioassay was performed by using an ethyl acetate crude extract of the actinobacterial isolates (Foss et al., 2014). Crude extract of actinobacterial isolates was recovered from the culture filtrate by solvent extraction using ethyl acetate in a 1:1 ratio (v/v). The dried ethyl acetate actinobacterial extract was dissolved in 10% Dimethyl Sulfoxide (DMSO) at a concentration of 1 mg/mL for the antifungal bioassay. 30µL of the extract was loaded onto sterile discs (6 mm diameter) placed on PDA plates seeded with fungal pathogens (0.5 McFarland turbidity standards). Amphotericin B (30 µg/disc) served as a positive control, while a 10% DMSO-loaded disc served as a negative control. Antifungal activity was observed after 48-72 hours of incubation at 28°C.

#### Detection of chitinase gene

The chitinase gene was screened in endophytic actinobacteria with antifungal and chitinase activities using GAIF and GAIR (18 family chitinase gene) primers (Williamson et al., 2000). The PCR program was set as 5 minutes at 95°C for initial denaturation, 30 seconds at 98°C for denaturation, 30 seconds at 50°C for annealing, 1 minute at 72°C for extension, and 10 minutes at 72°C for the final extension. The reaction was carried out using a BIO-RAD T100 thermal cycler.

#### Concluding assessment of *in vitro* PGP and biocontrol traits

A scoring scale named bonitur scale was created to evaluate the most potent strains among the endophytic actinobacterial isolates with high PGP and biocontrol potential (Passari et al., 2015). This scoring scale assigned points for each screened PGP and biocontrol trait, with a maximum score of 41. Depending on the percentage of inhibition, the top score of antifungal activity against each pathogen is 3, for 10 fungal phytopathogens leading to a total of 30 points.

For the assessment of PGP traits, 3 points were assigned for each of the IAA production and phosphate solubilization traits, depending on their quantitative value. Siderophore, ammonia, ACC deaminase, and chitinase enzyme production scored 1 point each.

#### Characterization of *Streptomyces* sp. VCLA3 and *Streptomyces* sp. RVRA7

##### Cultural characteristics

*Streptomyces* sp. VCLA3 and *Streptomyces* sp. RVRA7 were cultured on different culture media (ISP2, ISP3, ISP4, ISP6, ISP7, GML, and Sabouraud Agar Media) at 28°C for 7–10 days (Shirling and Gottlieb (1966). The colony characteristics such as growth, production of soluble pigments, color of aerial and substrate mycelium were studied. Growth at various temperatures (4–45°C), and pH (3-11) was determined on ISP2 medium at 28°C for 14–21 days.

Morphological characteristics of *Streptomyces* sp. VCLA3 and *Streptomyces* sp. RVRA7 was observed under a Scanning Electron Microscope (SEM).

##### Biochemical profile

Biochemical characterization of the potent endophytic actinobacterial strains *Streptomyces* sp. VCLA3 and *Streptomyces* sp. RVRA7 was performed with several carbohydrate utilization tests such as esculin hydrolysis, β-galactosidase activity, malonate, mannitol, and citrate utilization. The KB009 Hi Carbohydrate kit was used for the experiment (HiMedia, India). Actinobacterial broth cultures were centrifuged at 3500 g, and the cells were amended in 0.9% NaCl saline solution after multiple washes. Each kit’s well was loaded with actinobacterial suspension (50 μl) and incubated for 4 to 7 days at 28°C. Changes in the media color indicate a positive result.

##### Gas chromatography-mass spectrometry analysis

Ethyl acetate extracts of potent actinobacterial isolates *Streptomyces* sp. VCLA3 and *Streptomyces* sp. RVRA7 was analyzed through GC-MS to identify the chemical compounds. Actinobacterial strains were inoculated from well-grown culture plates into 1L Erlenmeyer flasks, each containing 200 ml of GLM medium. Fermentation was carried out for 7 days at 28°C on a shaking incubator at 180 rpm. Centrifugation was carried out at 7000 g for 20 minutes and filtered with a 0.2 μm Whatman filter paper to separate the cultured cells from the supernatant. The supernatant was extracted by mixing the cell-free culture with ethyl acetate in a 1:1 (v/v) ratio and mixed vigorously. The organic phase was removed in a separatory funnel (Bharucha et al., 2013). A rotary evaporator was used to evaporate the extracted ethyl acetate fraction at 45°C. The extracted crude was collected, diluted in HPLC grade methanol, and filtered through a 0.2 µm filter. Finally, the bioactive metabolites present in the crude extract of actinobacterial strains were analyzed using GC-MS (Sun et al., 2015) with minor modifications. The machine column used was EB-5MS, measuring 30 m, 0.25 m, and 25 mm in length, thickness, and internal diameter, respectively. Helium (1 ml/minute) was used as a carrier gas to inject 0.2 µm filtered samples at 270°C. The temperature of the column was programmed at 40°C for 5 minutes, increased to 200°C at a rate of 10°C/minutes for 10 minutes, then isothermally maintained at that temperature for 5 minutes before rising to 290°C at 10°C/minutes and lastly, kept for 5 minutes. Electron ionization mode operated the mass spectrometer at 70 eV with a continuous scan from 45 to 600 m/z. The peaks were detected by comparing the mass spectra with the library of the National Institute of Standards and Technology (NIST, USA).

##### Scanning electron microscope analysis

SEM was used to study and observe the effect of strain *Streptomyces* sp. VCLA3 on test fungal pathogen *F. oxysporum* (MTCC 4633). Fungal cells were scraped from the side of the inhibition zone and processed for SEM analysis as described by Supaphon et al., 2013, with slight alterations. Glutaraldehyde 2.5% (v/v) in phosphate buffer saline (PBS) was used to fix the treated cells overnight and then washed with PBS (pH 7.4) and water. Dry acetone was used to dehydrate cells at various concentrations, 30%, 50%, 70%, and 100%. TMS was used at the final step. After curing for 30 minutes, the cells were secured to a steel stub with double-sided carbon tape and analyzed under SEM (Germany, Zeiss Sigma VP).

#### Assessment of plant growth promotion

##### Seed germination assay

Chili seeds were used for seed germination plate assay to test the effectiveness of the potent endophytic actinobacterial strains. The chili seeds were first cleaned with 70% ethanol, then surface sterilized for 5 minutes with 1% NaOCl, and washed with sterile distilled water. Seeds were inoculated with broth culture (10^3^ cells/ml) of each *Streptomyces* strain for two hours. On sterile petri plates with sterile moistened filter papers, 10 chili seeds were distributed. The plates were kept at room temperature and regularly sprayed with sterile distilled water. Surface sterilized chili seeds without bacterial suspension inoculation were treated as a control. All plates were monitored for seed germination, and the germination rate was determined using the following formula described by Ranganathan and Thavaranjit, 2015.

% germination = number of seeds that germinated/total number of seeds ×100.

##### Growth promotion experiment

To know the efficacy of plant growth promotion *in vivo*, two potent endophytic actinobacterial strains, VCLA3 and RVRA7, were chosen based on their *in vitro* PGP abilities. Chili (*Capsicum annum*) plant was taken as a test plant for the experiment due to their feasibility to study under lab conditions. For inoculum preparation, mass culture was obtained by inoculating the pure actinobacterial cultures onto GLM broth individually and grown for 7 days at 28°C at 180 rpm under agitated conditions. The broth cultures were centrifuged at 10,000 rpm for 15 minutes, washed with PBS (pH 7.4) two times, and diluted to obtain a final concentration of 10^8^ CFU/ml. 10 ml of the endophytic actinobacterial suspension was used to treat targeted plants. First healthy chili seeds were soaked in distilled water for 24 h before subjecting to surface sterilization according to the protocol described by Passari et al., 2015. Then the seeds were washed with 70% ethanol for 5 minutes, surface sterilized with 1% NaOCl for 5 minutes, and washed multiple times with sterile distilled water. Sterilized seeds were then transferred onto moist sterile tissue paper. To promote germination, the seeds were provided with 2 ml sterile distilled water for 2 to 3 days under dark conditions. After 3 weeks of incubation, the germinated chili seeds were planted separately in plastic pots filled with unsterilized garden soil. The experiment was conducted with three treatments, and five replicates each, including treatment 1: VCLA3 inoculation, treatment 2: RVRA7 inoculation, and treatment 3: inoculation of consortia of both the strains and control plants without bacterial inoculum. The plants were watered twice daily to retain the moisture of the soil. Treatments were done at 7 day intervals, and harvesting was done after 35 and 70 days. All vegetative parameters of the plants, such as leaf no, shoot and root length, shoot fresh-dry weight, root fresh-dry weight, and chlorophyll content, were recorded and compared to the control.

##### Chlorophyll estimation

The Hiscox and Israelstam (2011) method was used to estimate the chlorophyll in the leaf tissues of both treated and control chili plants. Leaf tissues were cut into fractions after rinsing in distilled water.100 mg of the leaf tissue was put into vials along with 7 ml of Dimethyl Sulfoxide (DMSO) (HiMedia) and incubated at 65°C for 1 hr to recover the chlorophyll into the fluid without crushing. The extract was put into a graduated tube, and DMSO was added to make a final volume of 10 ml. At 645 nm and 663 nm, absorbance was recorded in a multimode reader by taking DMSO as a blank. The following formula was employed to calculate chlorophyll a and b.

Chla = 11.75 × A663 − 2.35 × A645

Chlb = 18.61 × A645 − 3.96 × A663

#### Data analysis

All PGP and antifungal experiments were done by keeping three replicates for each test and represented as mean ± standard error (SE). One-way ANOVA (analysis of variance) was used to examine the plant growth parameters statistically. The threshold for statistical significance was set at p<0.05. Principal component analysis (PCA) in MATLAB R was used to assess the correlations between the treatments and vegetative parameters (2017a). A fold change analysis was performed to ascertain the difference between the means of the control group and the treatments. Log2 scale was used to express the final value for the up-regulation or down-regulation to be equally far from the baseline.

## Results

**Figure 1:**
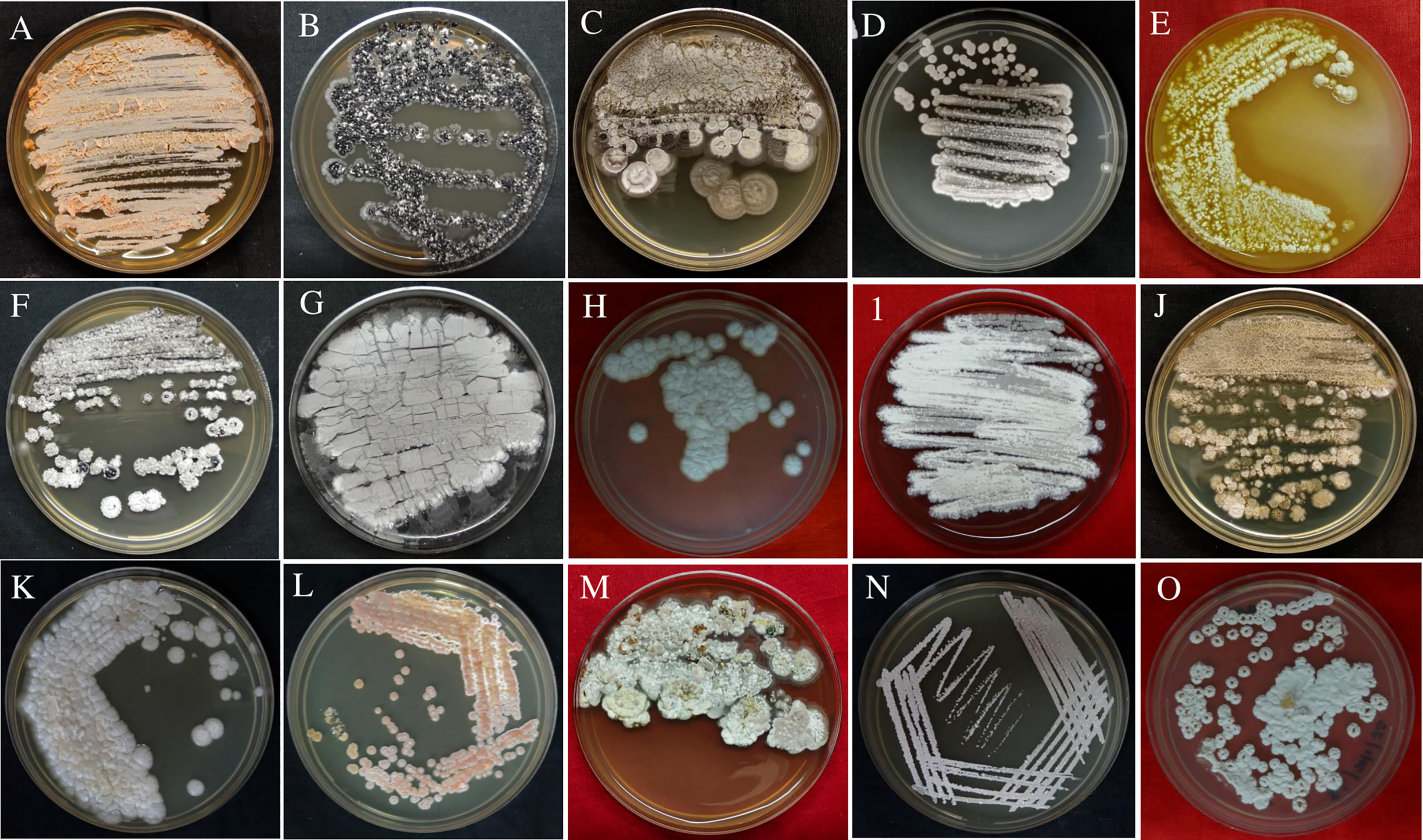
Pure culture plates of endophytic actinobacterial isolates obtained from root and leaf tissues of ten orchid species of Assam, India. The isolates streaked on GLM media were **(A)** DDRA3; **(B)** DCLA2; **(C)** DDLA2; **(D)** DNRA2; **(E)** CARA1; **(F)** DNRA1; **(G)** DMRA3; **(H)** DFRA2; **(I)** DCRA3; **(J)** DDLA1; **(K)** RVRA8; **(L)** RRLA1; **(M)** DMLA8; **(N)** DNLA1; **(O)** CARA3.

51 culturable endophytic actinobacteria were recovered from surface-sterilized leaves and roots of ten orchid species with seven different enriched culture media. Based on the colony form, ability to produce aerial hyphae, substrate mycelia, and production of diffusible pigments, isolates were identified and characterized (**Table 1** and **Figure 1**). Out of the 51 endophytic actinobacterial isolates, the majority, 33 (64.7%) isolates were recovered from roots, followed by 18 (35.29%) isolates from leaves.

**Table 1:** Morphological characterization of endophytic actinobacteria isolated from ten orchid species of Assam on the basis of phenotypic traits.

Sl. No.	Isolate Code	Colony morphology	Aerial mycelia color	Substrate mycelia Color	Diffusible pigment	Color Series	Texture	Tissue origin
1	DNRA1	Irregular, umbonate	Gray	Gray	NP	Gray	Dry	Root
2	DNRA2	Irregular, raised	White	White	NP	White	Rough	Root
3	DNRA3	Irregular flat	White	Brown	NP	White	Smooth	Root
4	DNRA4	Irregular umbonate	Off white	Off white	NP	White	Dry	Root
5	DNRA5	Irregular, raised	White	Brown	NP	White	Rough. Spongy	Root
6	DNLA1	Irregular, umbonate	White	White	NP	White	Dry	Leaf
7	DNLA2	Irregular raised	Gray	Gray	NP	Gray	Dry, Sticky	Leaf
8	DNLA13	Irregular raised	Blackish gray	Dark brown	NP	Gray	Dry	Leaf
9	DCRA1	Circular umbonate	White	Light brown	NP	White	Dry	Root
10	DCRA2	Circular raised	Off white	Gray	NP	White	Dry	Root
11	DCRA3	Undulate, flat	White	Brown	NP	White	Rough, dry	Root
12	DCLA1	Irregular raised	Off white	Light brown	NP	White	Dry	Leaf
13	DCLA2	Irregular undulate	Black	Black	NP	Gray	Rough dry	Leaf
14	DCLA5	Curled, umbonate	Brown	Brown	NP	Brown	Rough, dry	Leaf
15	DMRA1	Irregular raised	Off white	Light cream	NP	White	Dry	Root
16	DMRA2	Circular umbonate	Gray	Blackish gray	NP	Gray	Dry	Root
17	DMRA3	Irregular raised	Gray	Gray	NP	Gray	Dry	Root
18	DMLA8	Irregular umbonate	Off white	Off white	NP	White	Rough dry	Leaf
19	DDRA1	Irregular umbonate	Cream white	Brown	NP	White	Dry	Root
20	DDRA2	Circular umbonate	Brown	Brown	NP	Brown	Dry	Root
21	DDRA3	Circular raised	Orange	Cream white	NP	Orange	Dry	Root
22	DDRA4	scalloped, raised	Orange	Orange	Yellow	Orange	Smooth	Root
23	DDLA1	Circular pulvinate	Brownish-yellow	Light yellow	Light brown	Yellow	Dry	Leaf
24	DDLA2	Irregular, umbonate	Off white	Off white	NP	White	Dry	Leaf
25	DFRA1	Irregular raised	Grey	Light brown	NP	Gray	Dry	Root
26	DFRA2	Undulate raised	White	White	NP	White	Dry	Root
27	DFRA3	Circular raised	Gray	Light brown	NP	Gray	Dry	Root
28	DFLA1	Circular, raised	White	Orange	yellow	White	Rough	Leaf
29	DFLA4	Curled, raised	Pink	Yellow	NP	Pink	Rough, powdery	Leaf
30	CARA1	Circular flat	Yellow	White	NP	Yellow	Dry	Root
31	CARA2	Circular, umbonate	Gray	Light brown	NP	Gray	Dry	Root
32	CARA3	Irregular, Pulvinate	White	Light brown	NP	White	Dry	Root
33	CALA1	Undulate flat	Peach	Peach	NP	Orange	Dry	Root
34	RRRA1	Irregular flat	Gray	Brown	NP	Gray	Dry/spongy	Root
35	RRLA1	Circular umbonate	Orange	Light brown	Light brown	Orange	Dry	Leaf
36	RRR46	Irregular umbonate	Off white	White	NP	White	Dry	Root
37	RVRA1	Irregular convex	Red	Red	Light pink	Red	Dry	Root
38	RVRA2	Irregular, raised	Cream white	Light brown	NP	White	Dry	Root
39	RVRA3	Circular, umbonate	Yellow	Brown	NP	Yellow	Dry	Root
40	RVRA4	Circular, convex	Pink	Yellow	NP	Pink	Dry	Root
41	RVRA6	Undulate raised	yellow	Light brown	NP	Yellow	Dry	Root
42	RVRA7	Circular, crateriform	White	white	NP	White	Rough dry	Root
43	RVRA8	Irregular pulvinate	White	Light brown	Light brown	White	Rough dry	Root
44	RVLA1	Circular convex	Dark gray	Dark brown	NP	Gray	Rough dry	Leaf
45	MORA11	Irregular raised	Dark gray	Black	Black	Gray	Dry/powdery	Root
46	MORA2	Circular crateriform	Light brown	Light brown	NP	Brown	Dry	Root
47	MOLA1	Irregular, pulvinate	Gray	Brown	Light brown	Gray	Dry	Leaf
48	VCLA1	Circular raised	Off white	Light brown	NP	White	Dry	Leaf
49	VCLA3	Undulate raised	Gray	Light brown	NP	Gray	Dry	Leaf
50	VCRA1	Irregular umbonate	Cream white	Cream white	NP	White	Dry	Root
51	VCRA2	Circular raised	White	Yellow	Yellow	White	Rough dry	Root

### Molecular identification and characterization

**Figure 2:**
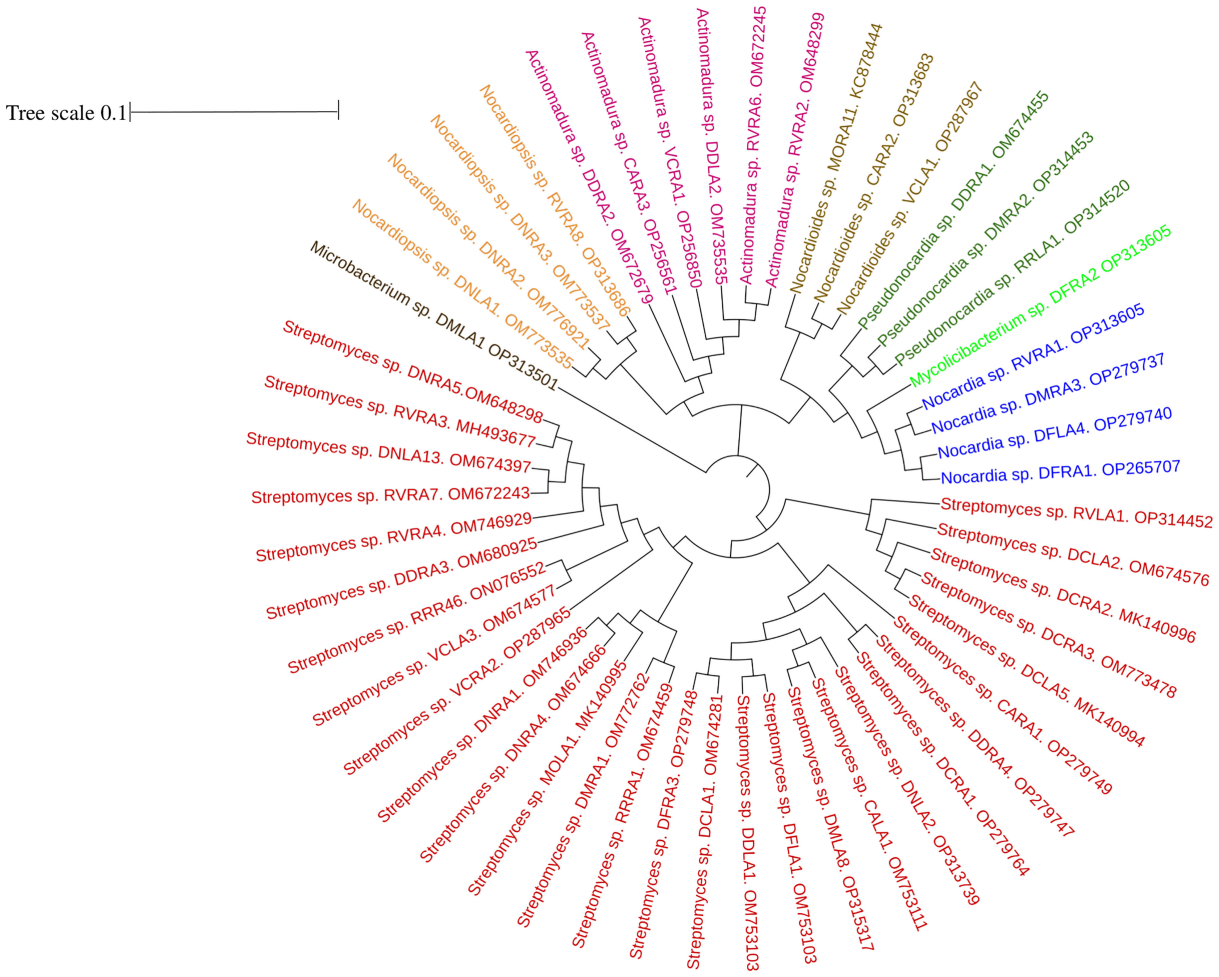
Phylogenetic tree based on NJ (Neighbor joining) method with 1000 bootstraps value showing 16S rRNA gene sequences of endophytic actinobacteria associated with ten orchid species. The scale bar represents the distance between the origin and most distant taxa.

16S rRNA gene sequences revealed a diverse community of actinobacterial isolates belonging to eight different genera, with 96.65–100 percent similarity in the sequence findings obtained from EzTaxon. GenBank accession numbers for 16s rRNA sequence of all the endophytic actinobacterial isolates were mentioned in **Supplementary Table 2**. Among 51 endophytic actinobacterial isolates obtained from ten orchid species, *Streptomyces* was dominant with 29 (56.86%) isolates. Other species include *Actinomadura* (6), *Nocardia* (4), *Noacrdiopsis* (4), *Pseudonocardia* (3) *Nocardioides* (3), *Microbacterium* (1), and *Mycolicibacterium* (1). The strains are from the orders Streptomycetales, Streptosporangeales, Pseudonocardiales, Prepionibacteriales, Micrococcales, and Cornybacteriales. To study the relationships among the endophytic actinobacterial isolates, 16S rRNA gene sequences were aligned with Clustal W to obtain a phylogenetic tree based on the NJ method with 1000 bootstraps. The phylogenetic tree demonstrated three major clades: one with the subclades of 29 strains of *Streptomyces* sp., the *Microbacterium* sp. formed a distinctively separate clade, and the other clade with the genus of *Nocardia*, *Nocardiopsis*, *Nocardioides*, *Pseudonocardia*, *Actinomadura*, and *Mycolicibacterium* (**Figure 2**).

### Plant growth promoting traits of endophytic actinobacteria

**Figure 3:**
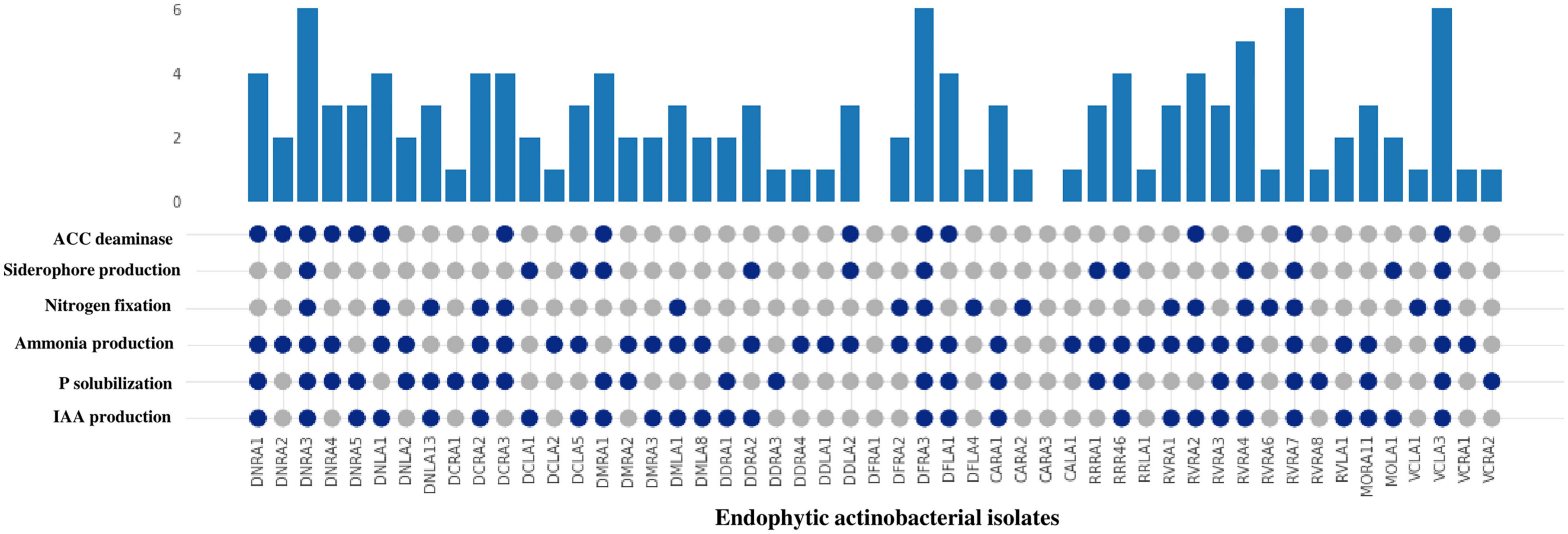
Comparison of PGP traits (IAA production, P (phosphate) solubilization, ammonia production, nitrogen fixation, siderophore production and ACC deaminase) among 51 endophytic actinobacterial isolates. The bar value represents the total no of PGP traits shown by the isolates. The presence and absence of PGP traits are represented by the blue and gray circles, respectively. 49 isolates exhibited presence of at least one PGP traits except isolates DFRA1 and CARA3. Isolates DNRA3, DFRA3, RVRA7 and VCLA3 showed the presence of all the PGP traits.

All 51 endophytic actinobacterial isolates were assessed for various PGP traits *in vitro*, and 49 (96.07%) isolates exhibited at least one PGP trait (**Table 2**). In addition, four isolates demonstrated positive results for all the tested PGP characters, including IAA synthesis, phosphate solubilization, siderophore, ammonia production, nitrogen fixation, and ACC deaminase activity (**Figure 3** and **Supplementary Figure 1**).

**Table 2:** Plant growth promotion traits and extracellular enzyme production by endophytic actinobacteria associated with orchid.

Sl. no.	Strain code	PGP traits						Extracellular enzyme productions			
		IAA production(µg/ml)	P solubilization (µg/ml)	Ammonia production (µmol/ml)	N_2_ fixation	Siderophore production (%)	ACC deaminase	Chitinase	Protease	Cellulase	Pectinase
1	DNRA1	20.7 ± 0.4	289.7 ± 11.1	2.1 ± 0.1	–	ND	+	–	+	–	–
2	DNRA2	ND	ND	3.4 ± 0.1	–	ND	+	–	–	–	+
3	DNRA3	13.6 ± 0.2	134.3 ± 5.2	3.3 ± 0.2	+	21.3 ± 0.2	+	–	+	–	–
4	DNRA4	ND	80.5 ± 6.0	3.1 ± 0.5	–	ND	+	+	+	+	–
5	DNRA5	32.2 ± 0.5	112.4 ± 10	ND	–	ND	+	+	–	–	+
6	DNLA1	14.6 ± 0.08	ND	3.6 ± 0.1	+	ND	+	+	+	+	+
7	DNLA2	ND	169.6 ± 6.2	2.3 ± 0.1	–	ND	–	–	–	–	–
8	DNLA13	5.8 ± 0.08	123.5 ± 2.8	ND	+	ND	–	+	–	–	–
9	DCRA1	ND	61.1 ± 4.4	ND	–	ND	–	–	+	–	+
10	DCRA2	24.3 ± 0.1	89.8 ± 3.0	4.5 ± 0.2	+	ND	–	+	+	+	–
11	DCRA3	ND	193.1 ± 9.2	3.7 ± 0.3	+	ND	+	–	+	–	–
12	DCLA1	12.8 ± 0.2	ND	ND	–	40.5 ± 0.6	–	+	–	–	–
13	DCLA2	ND	ND	3.1 ± 0.2	–	ND	–	–	–	+	–
14	DCLA5	14.4 ± 0.06	ND	5.9 ± 0.2	–	32.1 ± 0.3	–	–	+	–	–
15	DMRA1	10 ± 0.05	76.3 ± 4.2	ND	–	53.2 ± 0.5	+	–	–	–	–
16	DMRA2	ND	181.2 ± 2.3	3.9 ± 0.3	–	ND	–	–	+	–	+
17	DMRA3	18.5 ± 0.2	ND	3.4 ± 0.6	–	ND	–	–	–	–	–
18	DMLA1	29.1 ± 0.03	ND	2.7 ± 0.2	+	ND	–	–	–	–	–
19	DMLA8	7.5 ± 0.1	ND	0.9 ± 0.1	–	ND	–	–	+	+	–
20	DDRA1	22.7 ± 0.1	124.9 ± 0.8	ND	–	ND	–	–	–	–	–
21	DDRA2	10.3 ± 0.2	ND	1.5 ± 0.1	–	26.1 ± 0.8	–	+	–	–	–
22	DDRA3	ND	180.2 ± 1.5	ND	–	ND	–	–	+	+	+
23	DDRA4	ND	ND	3.1 ± 0.1	–	ND	–	+	–	–	–
24	DDLA1	ND	ND	1.3 ± 0.2	–	ND	–	–	–	+	–
25	DDLA2	ND	ND	2.2 ± 0.2	–	39.2 ± 0.3	+	–	+	–	–
26	DFRA1	ND	ND	ND	–	ND	–	–	–	–	–
27	DFRA2	ND	ND	2.5 ± 0.3	+	ND	–	–	–	+	–
28	DFRA3	18.6 ± 0.08	85.8 ± 3.2	3.2 ± 1.1	+	59.4 ± 0.4	+	–	–	–	–
29	DFLA1	9.4 ± 0.2	93.2 ± 2.3	1.9 ± 0.2	–	ND	+	+	+	–	–
30	DFLA4	ND	ND	ND	+	ND	–	+	–	–	–
31	CARA1	23.3 ± 0.1	132.5 ± 2.3	3.9 ± 0.4	–	ND	–	–	–	–	–
32	CARA2	ND	ND	ND	+	ND	–	–	–	–	–
33	CARA3	ND	ND	ND	–	ND	–	–	+	–	–
34	CALA1	ND	ND	2.5 ± 0.4	–	ND	–	–	–	–	–
35	RRRA1	ND	96.3 ± 2.0	2.6 ± 0.1	–	25.7 ± 0.6	–	–	–	–	–
36	RRR46	8.4 ± 0.2	213.2 ± 0.3	2.3 ± 0.1	–	61.2 ± 0.2	–	+	+	+	–
37	RRLA1	ND	ND	4.8 ± 0.2	–	ND	–	–	+	–	+
38	RVRA1	4 ± 0.08	ND	2.9 ± 0.1	+	ND	–	+	–	–	–
39	RVRA2	33.9 ± 0.4	ND	1.3 ± 0.1	+	ND	+	+	+	–	+
40	RVRA3	27.1 ± 0.1	148.3 ± 1.2	2.6 ± 0.1	–	ND	–	+	+	+	–
41	RVRA4	11.3 ± 0.2	103.6 ± 0.4	3.1 ± 0.2	+	38.3 ± 0.5	–	+	–	–	–
42	RVRA6	ND	ND	ND	+	ND	–	–	+	–	–
43	RVRA7	38.2 ± 0.2	239.1 ± 0.8	4.9 ± 0.2	+	39.7 ± 0.2	+	+	–	–	–
44	RVRA8	ND	81.2 ± 0.2	ND	–	ND	–	+	–	–	+
45	RVLA1	19.1 ± 0.8	ND	1.5 ± 0.1	–	ND	–	–	–	–	–
46	MORA11	12.4 ± 0.1	90.3 ± 1.2	2.6 ± 0.1	–	ND	–	–	+	–	–
47	MOLA1	8.4 ± 0.1	ND	ND	–	21.3 ± 0.4	–	–	–	–	–
48	VCLA1	ND	ND	ND	+	ND	–	–	–	+	+
49	VCLA3	43.8 ± 0.2	258.2 ± 6.7	4.1 ± 0.4	+	31.3 ± 0.2	+	+	+	+	–
50	VCRA1	ND	ND	4.1 ± 0.4	–	ND	–	–	–	–	+
51	VCRA2	ND	115.6 ± 1.1	ND	–	ND	–	+	+	–	–

The values represent mean value (n=3), **±** (standard error, SE), (**+**) activity or growth, (**-**) represents negative activity or no growth. IAA, Indole Acetic Acid production; P solubilization, Phosphate solubilization; ND, Not detected.

### Phosphate solubilization and production of indole-3-acetic acid

Among the 51 endophytic actinobacterial isolates, 25 (49.01%) were identified as potential phosphate solubilizers based on the formation of a transparent halo zone surrounding the colony on pikovskaya’s agar plates. In quantitative estimation, phosphate solubilization range was found from 61.1±4.4 - 289.7±11.9 µg/ml. The highest phosphate solubilization was detected in *Streptomyces* sp. DNRA1 (289.7±11.9) µg/ml, followed by *Streptomyces* sp.VCLA3 (258.2±6.7) µg/ml and *Streptomyces* sp. RVRA7 (239.1±5.0) µg/ml.

Out of 51 endophytic actinobacterial isolates, 27 (52.94%) isolates showed the ability to biosynthesize IAA in the presence of l-tryptophan in media. Quantitative IAA production among the endophytic actinobacterial isolates ranged from 4.0 ± 0.08 - 43.8 ± 0.2 µg/ml. *Streptomyces* sp. VCLA3 produced the most IAA with 43.8 ± 0.2 µg/ml, followed by *Streptomyces* sp. RVRA7 with 38.2 ± 0.2 µg/ml and *Actinomadura* sp. RVRA2 with 33.9 ± 0.4 µg/ml. Moreover, 10 isolates have produced more than 20 µg/ml of IAA.

### Estimation of ammonia and siderophore production

The maximum number of isolates, 35 (68.62%) out of the total 51 isolates of endophytic actinobacteria, showed the ability to produce ammonia at concentrations ranging from 0.9 ± 0.1-5.9 ± 0.2 µmol/ml. Isolate *Streptomyces* sp. DCLA5 produced the maximum amount of ammonia (5.9 ± 0.2 µmol/ml). In addition, 17 (33.33%) isolates were grown on nitrogen-depleted media. NF and Jensen agar medium are nitrogen-free media for detecting nitrogen-fixing bacteria. The ability of endophytic actinobacterial isolates to grow on these mediums proves their ability to fix nitrogen in the atmosphere.

Siderophore production was detected in 13 (25.49%) isolates on CAS agar medium with the development of a clear orange halo zones around the colonies. The percentage of siderophore production by endophytic actinobacterial isolates ranged from 10 to 50%.

### Extracellular enzyme production assay

All 51 actinobacterial endophytes were qualitatively tested for secretion of extracellular enzymes such as protease, cellulase, pectinase, and chitinase, of which 22 (43.13%) isolates showed production of protease by degrading protein and formed a clear halos around colonies on SMA medium. 19 (37.25%) isolates showed positive results for chitinase enzyme production, 11 (21.56%) isolates showed pectinase production, and 12 (23.52%) isolates showed cellulase enzyme production and 14 (27.45%) isolates used ACC as the sole source of nitrogen and confirmed ACC deaminase (**Table 2** and **Supplementary Figure 1**).

### Evaluation of *in vitro* antifungal activity

**Figure 4:**
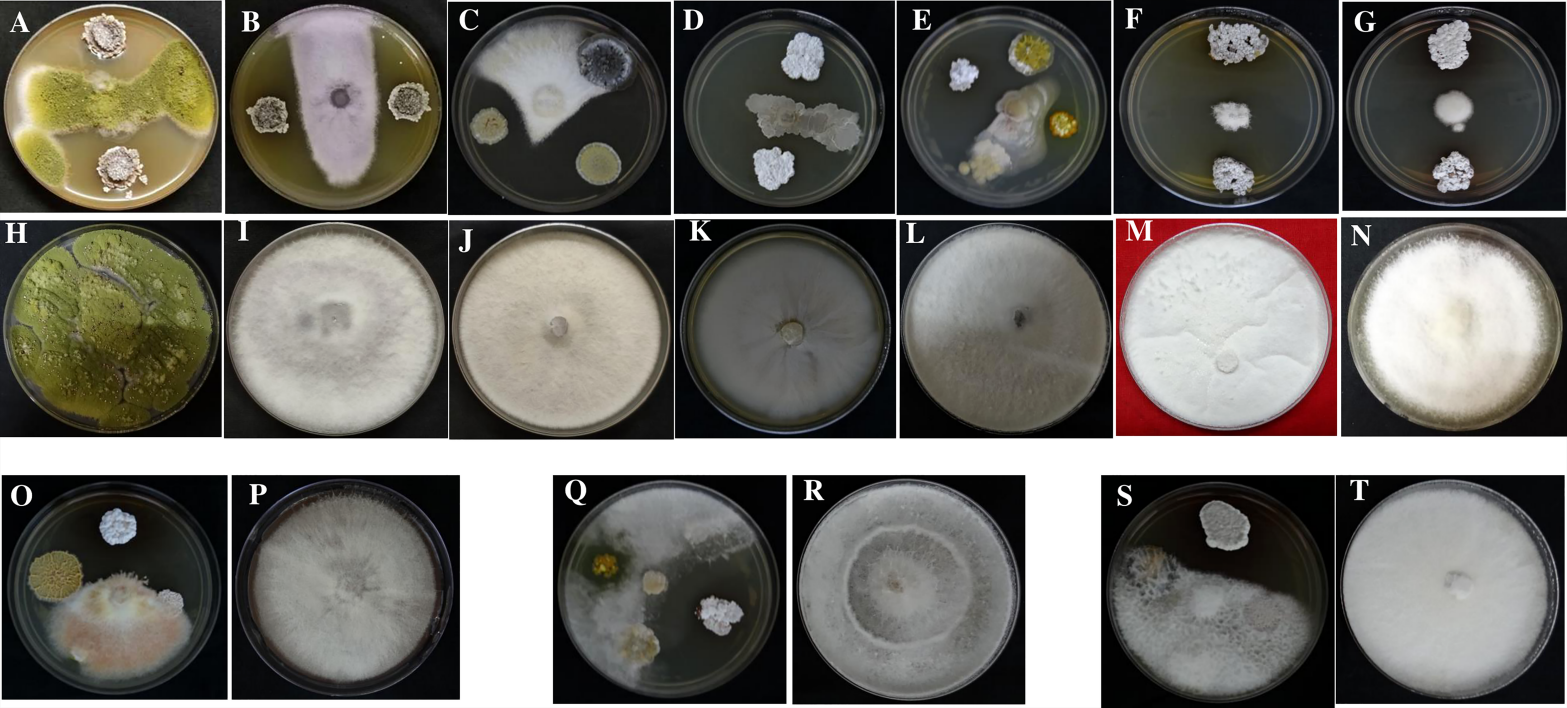
Antifungal activity of endophytic actinobacteria against fungal phytopathogens **(A)** *Colletotrichum capsici* (MTCC 8473) treatment and **(H)** Control; **(B)** *Fusarium oxysporum* (MTCC 284) treatment and **(I)** Control; **(C)** *Fusarium solani* (MTCC 365) treatment **(J)** Control; **(D)** *Nigrospora sphaerica* (KJ767520) treatment and **(K)** Control; **(E)** *Phellinus lamaensis* (ITCC 292) treatment and **(L)** Control; **(F)** *Poria hypobrunnea* (ITCC 4141) treatment and **(M)** Control; **(G)** *Glomerulla cingulata* (MTCC 2033) treatment and **(N)** Control; **(O)** *Rhizoctonia solani* (MTCC 4633) treatment and **(P)** Control; **(Q)** *Curvularia eragrostidis* (MTCC 8198) treatment and **(R)** Control; **(S)** *Pestalotiopsis theae* (ITCC 6599), treatment and **(T)** Control.

All 51 endophytic actinobacterial isolates were screened for antifungal activity against ten fungal phytopathogens, namely *Poria hypobrunnea* (ITCC 4141) *Rhizoctonia solani* (MTCC 4633)*, Fusarium oxysporum* (MTCC-284), *Phellinus lamaensis* (ITCC292), *Colletotrichum capsici* (MTCC-8473)*, Curvularia eragrostidis* (MTCC-8198)*, Pestalotiopsis theae* (ITCC 6599)*, Fusarium solani* (MTCC 365), *Glomerulla cingulata* (MTCC 2033), *Nigrospora sphaerica* (KJ767520) (Dutta et al., 2015). 27 (52.94%) isolates showed antagonism against at least one of the tested pathogens. 19 (37.25%) isolates suppressed the growth of *F. oxysporum*. 14 (27.45) isolates showed antagonism against *F. solani*. 15 (29.41%) and 13 (25.49%) isolates inhibited the growth of pathogens *P. thea* and *P.lamaensis*, respectively.7 (13.72%) isolates showed antagonism against *C. eragrostidis*. 10 (19.60%) and 8 (15.68%) isolates showed antagonism against *G. cingulata* and *N. sphaerica*, respectively. 15 (29.41%), 17 (33.33%), and 13 (25.49%) isolates inhibited the growth of *R. solani*, *C. capsici*, and *P. hypobrunneae* (**Figure 4** and **Table 3**). Moreover, 2 isolates have shown significant antifungal activity ranging from 48.7% to 92.2% against all the test fungal phytopathogens.

**Table 3:** Antifungal activity of endophytic actinobacteria associated with orchid against ten fungal plant pathogens.

Sl. no.	Strain code	% Inhibition of pathogen fungal mycelia									
		1	2	3	4	5	6	7	8	9	10
1	DNRA1	–	–	–	–	–	–	–	–	–	–
2	DNRA2	–	–	–	–	–	–	–	–	–	–
3	DNRA3	–	–	58.4 ± 0.4	–	–	–	–	–	46.7 ± 0.6	–
4	DNRA4	49.2 ± 0.9	46.6 ± 0.4	–	59.5 ± 0.1	41.2 ± 0.2	62.7 ± 0.6	–	–	–	–
5	DNRA5	54.3 ± 1.3	–	–	62.5 ± 0.8	46.5 ± 0.1	–	42.1 ± 0.4	–	45.2 ± 0.4	–
6	DNLA1	–	61.2 ± 0.6	–	49.6 ± 0.1	–	–	56.3 ± 1.1	–	–	–
7	DNLA2	–	–	–	–	–	–	–	–	–	–
8	DNLA13	82.4 ± 0.8	80 ± 1.2	58.4 ± 0.6	64.5 ± 0.3	55.7 ± 0.4	58.9 ± 0.6	48.7 ± 0.6	61.3 ± 0.8	84.5 ± 0.2	71.2 ± 0.8
9	DCRA1	67.5 ± 0.6	–	31.6 ± 0.4	53.4 ± 0.4	49.3 ± 0.2	65.9 ± 1.3	43.1 ± 0.4	–	–	58.7 ± 0.1
10	DCRA2	52.3 ± 0.4	–	49.5 ± 0.2	58.2 ± 0.3	27.2 ± 0.4	–	–	–	–	–
11	DCRA3	–	–	–	–	–	–	–	–	–	–
12	DCLA1	51.5 ± 0.3	58.5 ± 0.4	–	43.6 ± 0.9	–	–	40.2 ± 0.6	–	–	–
13	DCLA2	–	–	–	–	–	–	–	–	–	–
14	DCLA5	–	46.2 ± 0.9	58.6 ± 0.8	43.4 ± 0.2	–	48.5 ± 0.2	–	–	–	–
15	DMRA1	–	–	–	–	–	–	–	–	–	–
16	DMRA2	–	–	–	–	–	–	–			
17	DMRA3	–	–	–	–	–	–	–	–	–	–
18	DMLA1	–	–	53.2 ± 0.2	–	57.4 ± 0.4	–	59.2 ± 0.7	–	–	
19	DMLA8	–	–	–	–	–	–	–	–	–	–
20	DDRA1	–	–	–	–	–	–	–	–	–	–
21	DDRA2	43.3 ± 0.7	–	48.5 ± 0.6	57.3 ± 0.4	43.2 ± 0.2	–	55.3 ± 0.1	–	–	–
22	DDRA3	–	–	–	–	–	–	–	–	–	–
23	DDRA4	–	59.3 ± 0.3	42.1 ± 1.1	46.2 ± 0.4	–	–	–	–	38.6 ± 0.4	–
24	DDLA1	–	–	–	–	–	–	–	–	–	–
25	DDLA2	–	–	–	–	–	–	–	–	–	–
26	DFRA1	–	–	–	–	–	–	–	–	–	–
27	DFRA2	–	–	–	–	–	–	–	–	–	–
28	DFRA3	46.6 ± 0.8	–	–	–	–	–	–	–	64.3 ± 0.4	55 ± 0.9
29	DFLA1	–	61.3 ± 0.4	41.9 ± 0.9	43.2 ± 0.9	51.2 ± 0.1	40 ± 0.2	51.1 ± 0.3	–	–	–
30	DFLA4	–	65.71 ± 1.1	28.2 ± 0.6	46.7 ± 0.3	–	38.8 ± 0.3	–	–	–	45.6 ± 0.4
31	CARA1	–	–	–	–	–	–	–	–	–	–
32	CARA2	–	–	52.1 ± 0.3	–	–	–	41.2 ± 0.4	–	–	–
33	CARA3	–	–	–	–	–	–	–	–	–	–
34	CALA1	–	–	–	–	–	–	–	–	–	–
35	RRRA1	–	–	–	–	–	–	–	–	–	–
36	RRR46	53.2 ± 0.3	–	–	62 ± 0.6	64.4 ± 0.4	60 ± 0.8	–	–	51.1 ± 0.4	41.8 ± 0.2
37	RRLA1	–	44.4 ± 0.2	–	–	58.6 ± 0.2	–	–	44.2 ± 0.4	–	–
38	RVRA1	41.9 ± 1.1	39.6 ± 0.8	47.7 ± 0.4	26.1 ± 0.9	–	–	–	–	51.3 ± 0.8	50 ± 0.4
39	RVRA2	–	–	–	–	61.1 ± 0.4	31.5 ± 0.2	–	–	–	56.6 ± 0.6
40	RVRA3	–	–	–	43.2 ± 0.8	51.3 ± 0.3	58.1 ± 0.6	–	45 ± 0.4	–	–
41	RVRA4	–	–	–	57 ± 0.4	48.5 ± 0.5	43.1 ± 0.1	41.2 ± 0.4	31.2 ± 0.4	–	–
42	RVRA6	–	–	–	–	–	–	–	–	–	35.4 ± 0.3
43	RVRA7	42.1 ± 1.0	51.1 ± 1.5	68.3 ± 0.5	72.3 ± 1.3	77.2 ± 0.6	62.4 ± 1.1	56.5 ± 1.2	74.4 ± 0.4	–	–
44	RVRA8	–	58.8 ± 0.4	–	–	53.3 ± 1.4	–	67.6 ± 0.4	–	–	58.4 ± 0.4
45	RVLA1	–	–	–	–	–	–	–	–	–	–
46	MORA11	–	–	–	–	–	–	–	–	–	–
47	MOLA1	–	–	–	–	–	–	51.2 ± 04	–	–	–
48	VCLA1	–	–	–	–	–	–	–	–	–	–
49	VCLA3	90.2 ± 0.7	61.1 ± 0.6	71.1 ± 1.2	45.5 ± 1.4	88.8 ± 1.3	91.1 ± 1.7	65.51 ± 1.2	83.2 ± 0.8	–	–
50	VCRA1	–	–	–	–	–	–	–	–	–	–
51	VCRA2	83.3 ± 1.2	92.2 ± 1.2	80 ± 0.8	62.6 ± 1.1	81.1 ± 1.3	52.1 ± 0.5	78.3 ± 0.4	55.5 ± 0.9	85.1 ± 0.4	89.2 ± 0.2

The values represent mean value (n=3), ± (standard error, SE), (-) =No activity; 1- *Poria hypobrunnea* (ITCC 4141); 2- *Fusarium solani* (MTCC 365); 3- *Rhizoctonia solani* (MTCC 4633); 4- *Fusarium oxysporum* (MTCC 284); 5- *Colletotrichum capsici* (MTCC 8473); 6-*Phellinus lamaensis* (ITCC 292); 7- *Pestalotiopsis theae* (ITCC 6599); 8- *Curvularia eragrostidis* (MTCC 8198); 9- *Nigrospora sphaerica* (KJ767520); 10-*Glomerulla cingulata* (MTCC 2033).

Ethyl acetate crude extract of the actinobacterial isolates showed promising antifungal activity. The antifungal activity of ethyl acetate crude extract of the actinobacterial isolates along with the controls (10% DMSO; negative control and antifungal amphotericin B positive control) against *Poria hypobrunnea* (ITCC 4141) and *Colletotrichum capsici* (MTCC-8473) were shown in **Supplementary Figure 2**.

### Detection of chitinase gene

The presence of chitinase gene was screened among endophytic actinobacterial isolates that demonstrated antifungal and chitinase activity in the plate experiment. PCR analysis identified the chitinase 18 glycosyl hydrolase family gene in 19 isolates. (**Supplementary Figure 3**).

### Analysis of PGP and biocontrol traits

The bonitur scale was used to select the most potent endophytic actinobacteria with PGP and biocontrol characters (Passari et al., 2015). The evaluation showed that the strain *Streptomyces* sp. VCLA3 is the highest-ranked, with an ∑ assessed value of 34, followed by *Streptomyces* sp. RVRA7 with an ∑ assessed value of 32 (**Table 4**). The top two endophytic actinobacterial strains, VCLA3 and RVRA7 were subsequently screened to evaluate their PGP efficacy under *in vivo* conditions by experimenting on chili plants.

**Table 4:** Bonitur scale assessment and ranking of orchid associated endophytic actinobacteria based on PGP and antifungal traits.

Isolate code	PGP traits					Antifungal mechanism		Antifungal traits										Total assessment score (41)	Rank
	IAA^a^	PS^b^	AM^c^	NF^d^	ACCD^e^	Sid^f^	Chi^g^	P.h^h^	F.s^i^	R.s^j^	F.o^k^	C.c^l^	P.l^m^	P.t^n^	C.e°	N.g^p^	G.c^q^		
VCLA3	3	3	1	1	1	1	1	3	3	3	2	3	3	3	3	0	0	34	1^st^
RVRA7	3	3	1	1	1	1	1	2	2	3	3	3	3	2	3	0	0	32	2^nd^
VCRA2	0	1	0	0	0	0	1	3	3	2	3	2	2	2	3	3	3	30	3^rd^
DNLA13	1	2	0	1	0	0	1	1	3	2	3	3	2	2	3	3	3	30	3^rd^
RRR46	1	3	1	0	0	1	1	2	0	0	3	3	2	0	0	2	2	21	4^th^
DNRA5	3	2	0	0	1	0	1	2	0	0	3	2	0	2	0	2	0	18	5^th^
DFLA1	1	1	1	0	1	0	1	0	3	2	2	2	2	2	0	0	0	18	5^th^

IAA^a^, Indole Acetic Acid production (1 ≤ 15, 2 = 15–30, 3 > 30 μg mL–1); PS^b^, Phosphate solubilization (1 ≤100, 2 = 100–200, 3 ≥ 300 µg mL−1); Amm^c^, Ammonia production (µmol mL−1) (1 = positive); Nit^d^, Nitrogen fixation (1 = positive); ACC^e^, ACC deaminase activity (1 = positive); Sid^f^, Siderophore production (%) (1 = positive); Chi^g^, Chitinase activity (1 = positive);

GI(%): Growth Inhibition %(1 = 10-30%, 2 = 30-60%, 3 = 60-100%). P.h^h^, *Poria hypobrunnea* (ITCC 4141); F.s^i^, *Fusarium solani* (MTCC 365); R.s^j^, *Rhizoctonia solani* (MTCC 4633); F.o^k^, *Fusarium oxysporum* (MTCC-284); C.c^l^, *Colletotrichum ingula* (MTCC-8473); P.l^m^, *Phellinus lamaensis* (ITCC292); P.t^n^, *Pestalotiopsis theae* (ITCC 6599); C.e°, *Curvularia eragrostidis* (MTCC-8198); N.g^p^, *Nigrospora sphaerica* (KJ767520) (Dutta et al., 2015); and G.c^q^, *Glomerulla cingulata* (MTCC 2033).

### Characterization of *Streptomyces* sp. VCLA3 and *Streptomyces* sp. RVRA7

#### Cultural characteristics

Both the *Streptomyces* strains VCLA3 and RVRA7 were able to grow well on all the eight tested culture media except actinomycetes isolation agar. Comparatively, poor growth was observed in actinomycetes isolation agar. Their cultural characteristics such as aerial mycelium, substrate mycelium, and soluble pigments on the tested culture medium were assessed (**Supplementary Figure 4**). *Streptomyces* sp. VCLA3 produced light brown color pigment on ISP3 media, whereas *Streptomyces* sp. RVRA7 produced light brown and light-yellow color pigments in ISP3 and streptomyces agar media respectively. Both strains represent long, straight, or flexuous spore chains under SEM analysis (**Supplementary Figure 5**). Both the strains were able to grow within the pH range 4 to 12, VCLA3 showed optimal growth at pH 9, and RVRA7 at pH 7. The strains were moderately thermotolerant, the temperature range for growth of VCLA3 was 20°C to 45°C and for RVRA7 it was 20°C to 42°C.

#### Biochemical characterization

Biochemical characterization of the top two potent strains, VCLA3 and RVRA7, was done by several carbohydrate utilization tests. VCLA3 showed positive for 25 tests, and RVRA7 exhibited 27 positive tests (**Supplementary Table 3** and **Supplementary Figure 1**).

#### Gas chromatography-mass spectrometry analysis

**Figure 5:**
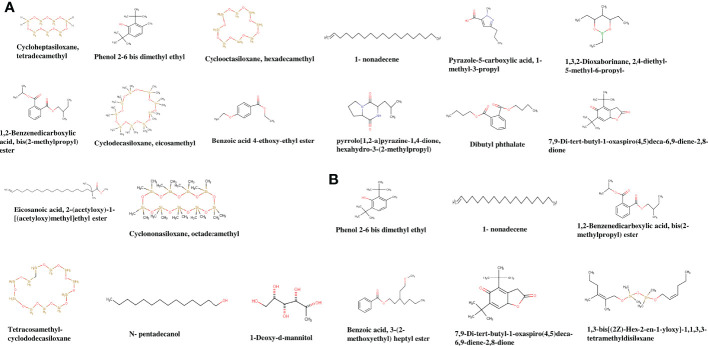
Chemical structures of the identified compounds from the crude extract of **(A)** *Streptomyces* sp. VCLA3 and **(B)** *Streptomyces* sp. RVRA7.

To know the major constituents, ethyl acetate extracts of the potent endophytic actinobacterial strains VCLA3 and RVRA7 were analyzed using GC-MS. The analysis identified several chemical compounds with various biological functions based on retention time and mass spectra with the National Institute of Standards and Technology (NIST) library (**Table 5** and **Figures 5A, B**). The crude extract of both the *Streptomyces* strains confirmed phenol, 2,4-bis-(1,1-dimethylethyl) as a major compound.

**Table 5:** Compounds identified from the ethyl acetate crude extract of *Streptomyces* sp. VCLA3 and *Streptomyces* sp. RVRA7 through GC-MS.

Compounds identified in ethyl acetate extract of VCLA3							
SL. No	Compound name	RT time	Area%	Molecular formula	Molecular mass	Activity	Reference
1	Cycloheptasiloxane, tetradecamethyl-	22.798	0.81	C_14_H_42_O_7_Si_7_	519.08	Antibacterial, immunomodulatory, antitumor, antifungal antioxidant, anti-inflammatory, anti-diabetic, and wound healing	Patil and Jadhav, 2014; Lutfia et al., 2021; Prasathkumar et al., 2021
2	phenol, 2,4-bis-(1,1-dimethylethyl)	24.563	17.88	C_15_H_24_O	220.35	Antimicrobial	Yogeswari et al., 2012
3	Pyrazole-5-carboxylic acid, 1-methyl-3-propyl	32.127	0.91	C_8_H_11_N_2_O_2_	167.186	No activity reported	
4	1-nonadecene	35.179	1.72	C_19_H_38_	266.51	Antimicrobial, antifungal, antituberculosis, antioxidant and anticancer activity	Rukachaisirikul et al., 2004; Lee et al., 2007; Samoi et al., 2012
5	Eicosanoic acid, 2-(acetyloxy)-1-[(acetyloxy)methyl]ethyl ester	35.494	5.65	C _21_H_42_O _2_	340.58	No activity reported	–
6	1,3,2-Dioxaborinane, 2,4-diethyl-5-methyl-6-propyl	36.232	4.5	C11H23BO2	198.11	No activity reported	–
7	1,2-Benzenedicarboxylic acid, bis(2-methylpropyl) ester	37.468	2.07	C_16_H_22_O_4_	278.3435	Antioxidant, cytotoxic, antimicrobial	Krishnan et al., 2014; Abdel-Kareem et al., 2019; Druzian et al., 2020
8	7,9-Di-tert-butyl-1-oxaspiro(4,5)deca-6,9-diene-2,8-dione	39.087	2.89	C_17_H_24_	276.371	No activity reported	–
9	pyrrolo[1,2-a]pyrazine-1,4-dione, hexahydro-3-(2-methylpropyl)	39.53	0.88	C_11_H_18_N_2_O_2_	210.2728	Antibiotic, biocontrol	Kiran et al., 2018
10	Cyclodecasiloxane, eicosamethyl	40.322	3.1	C_20_H_60_O_10_Si_10_	741.5394	Antirheumatic, hepatoprotective, and anti-spasmodic	Prasathkumar et al., 2021
11	Dibutyl phthalate	40.704	11.44	C16H22O4	278.34	No activity reported	–
12	Cyclooctasiloxane, hexadecamethyl	44.951	1.72	C_16_H_48_	593.232	Antibacterial, antifungal antioxidant, anti-inflammatory, anti-diabetic, and wound healing	Lutfia et al., 2021; Prasathkumar et al., 2021
13	Benzoic acid, 4-ethoxy-ethyl ester	59.155	0.41	C_11_H_14_O_3_	194.2271	Antimicrobial activity	Kalpana and Rajeswari, 2019
14	Cyclononasiloxane, octadecamethyl	60.134	2.48	C_18_H_54_O_9_Si_9_	666.169	Antifungal	Alghamdi and Ababutain, 2019
**Compounds identified in ethyl acetate extract of RVRA7**							
1	1-Deoxy-d-mannitol	6.745	2.68	C_6_H_14_O_5_	211.17	Increased tolerance to biotic and abiotic stress, protect against pathogen attack	Stoop et al., 1996
2	phenol, 2,4-bis-(1,1-dimethylethyl)	24.637	26.4	C_15_H_24_O	220.35	Antimicrobial	Yogeswari et al., 2012
3	n-pentadecanol	35.297	3.16	C_15_H_32_O	228.4140	Antibacterial, antidiabetic, anticancer	Chatterjee et al., 2018; Ali et al., 2021
4	Tetracosamethyl-cyclododecasiloxane	56.465	1.6	C_24_H_72_O_12_Si_12_	889.847	Phytochemical, antioxidant, and antimicrobial	Al Bratty et al., 2020; Pradhan et al., 2021
5	1,3-bis[(2Z)-Hex-2-en-1-yloxy]-1,1,3,3-tetramethyldisiloxane	57.81	1.16	C_16_H_34_O_3_Si_2_	330.610	No activity reported	–
6	Benzoic acid, 3-(2-methoxyethyl) heptyl ester	36.595	0.39	C_14_H_20_O_2_	220.3074	Antimicrobial	Kalpana and Rajeswari, 2019
7	1,2-Benzenedicarboxylic acid, bis(2-methylpropyl) ester	37.606	1.78	C_16_H_22_O_4_	278.3435	Antioxidant, cytotoxic, antimicrobial	Krishnan et al., 2014; Abdel-Kareem et al., 2019; Druzian et al., 2020
8	7,9-Di-tert-butyl-1-oxaspiro (4,5) deca-6,9-diene-2,8-dione	39.065	4.16	C17H24O3	276.4	No activity reported	–
9	1-Nonadecene	42.072	2.33	C_19_H_38_	266.51	Antimicrobial, antifungal, antituberculosis, antioxidant and anticancer activity	Rukachaisirikul et al., 2004; Lee et al., 2007; Samoi et al., 2012

In VCLA3 ethyl acetate extract 14 compounds were detected, which include (1) Cycloheptasiloxane, tetradecamethyl, (2) Phenol, 2,6-bis-(1,1-dimethylethyl) (3) Pyrazole-5-carboxylic acid, 1-methyl-3-propyl, (4) 1-nonadecene, (5) Eicosanoic acid, 2-(acetyloxy)-1-[(acetyloxy)methyl]ethyl ester, (6) 1,3,2-Dioxaborinane, 2,4-diethyl-5-methyl-6-propyl, (7) 1,2-Benzenedicarboxylic acid, bis(2-methylpropyl) ester, (8) 7,9-Di-tert-butyl-1-oxaspiro(4,5)deca-6,9-diene-2,8-dione, (9) pyrrolo[1,2-a]pyrazine-1,4-dione, hexahydro-3-(2-methylpropyl), (10) Cyclodecasiloxane, eicosamethyl, (11) Dibutyl phthalate, (12) Cyclooctasiloxane, hexadecamethyl, (13) Benzoic acid, 4 ethoxy-ethyl ester, (14) Cyclononasiloxane, octadecamethyl.

A total of nine compounds were identified in RVRA7, including (1) 1-Deoxy-d-mannitol, (2) phenol, 2,4-bis-(1,1-dimethylethyl), (3) n-pentadecanol, (4) n-pentadecanol, (5) 1,3-bis[(2Z)-Hex-2-en-1-yloxy]-1,1,3,3-tetramethyldisiloxane, (6) Benzoic acid, 3-(2-methoxyethyl) heptyl ester, (7) 1,2-Benzenedicarboxylic acid, bis(2-methylpropyl) ester, (8) 7,9-Di-tert-butyl-1-oxaspiro (4,5) deca-6,9-diene-2,8-dione, (9) 1-Nonadecene.

#### Scanning electron microscope analysis

**Figure 6:**
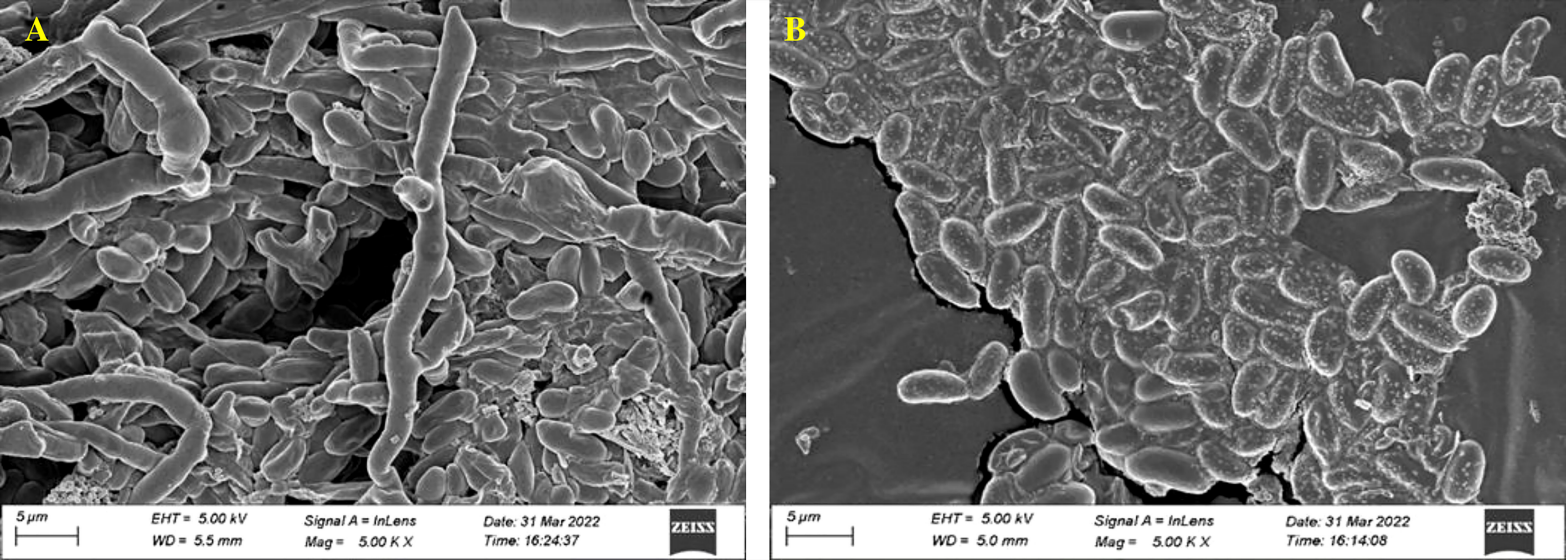
Fungal morphology of *F oxysporum* (MTCC 284) affected by *Streptomyces*. sp. VCLA3 under Scanning Electron Microscope (SEM). **(A)** Control, **(B)** Treatment.

The control (untreated) test fungal pathogen was uniform in structure and grew and spored luxuriously. The fungal spore on the treated sample showed major surface morphology change, confirming that the treatment has affected the fungal spores and restricted their further growth and development (**Figure 6**).

### *In vivo* plant growth promotion assay

#### Seed germination assay

**Figure 7:**
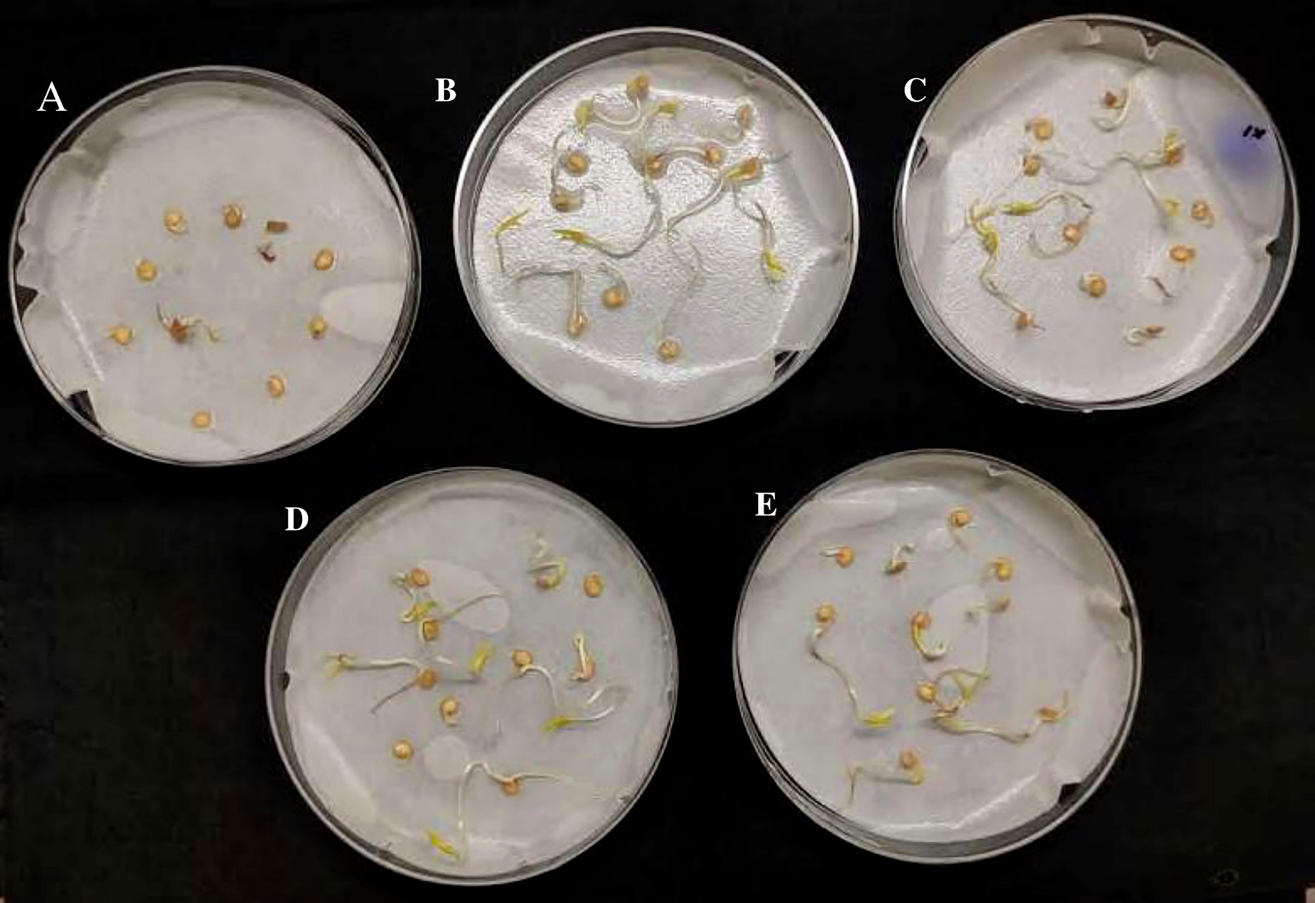
Seed germination plate assay in chili seeds, **(A)** Control; **(B, C)**. - chili seeds treated with *Streptomyces* sp. VCLA3; **(D, E)**- chili seeds treated with *Streptomyces* sp. RVRA7.

Chili seeds inoculated with actinobacterial strains VCLA3 and RVRA7 in seed germination plate assay showed better seedling growth in comparison to non-inoculated control. The treated seeds had a greater germination rate (100%) as compared to the control (80%) (**Figure 7**), indicating that the treatment enhanced the germination.

#### Growth promotion assay in chili plant

**Figure 8:**
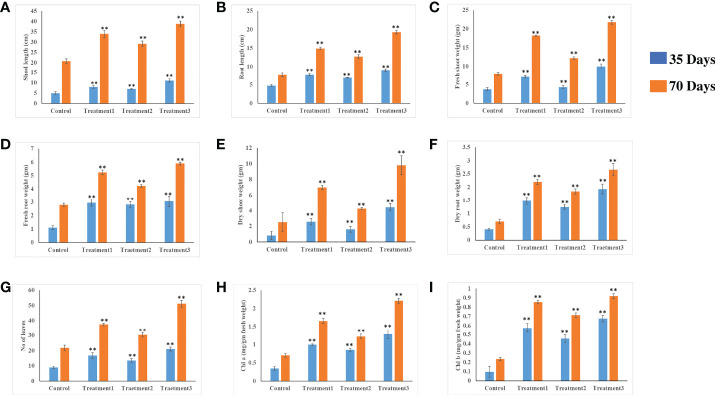
*In vivo* plant growth-promoting effect of endophytic actinobacterial isolates on chili plants. Treatment 1-VCLA3 Treatment 2-RVRA7, Treatment 3-VCLA3+RVRA7 **(A)** Shoot length (cm), **(B)** root length (cm), **(C)** Shoot weight fresh (g), **(D)** Root weight fresh (g), **(E)** Shoot weight dry (g), **(F)** Root weight dry (g), **(G)** No of leaves, **(H)** Chlorophyll a (mg/g fresh weight), **(I)** Chlorophyll b (mg/g fresh weight) after 35 and 70 days of inoculation. The values are mean ± Standard error (*n* = 5). ^∗∗^Represents statistical significance (*p* < 0.05, One-way ANOVA).

**Figure 9:**
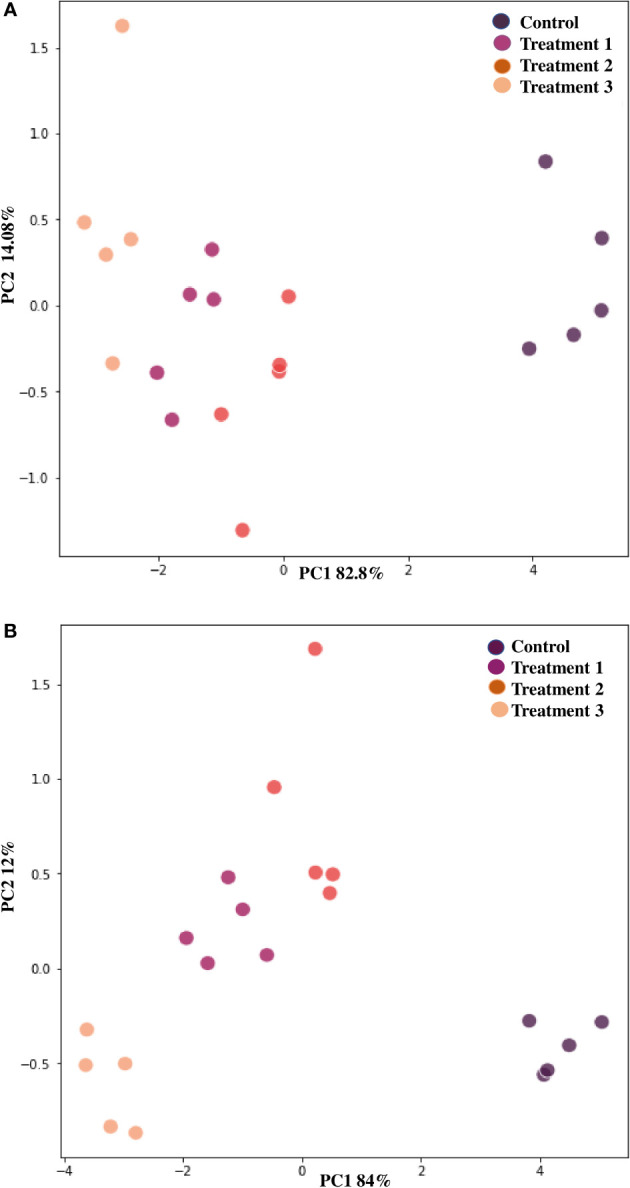
Principle component analysis based on the eigen values in a matrix on the PGP datasets to study the correlation between growth parameters and endophytic actinobacterial treatment in the PGP experiment. **(A)** After 35 days and **(B)** After 70 days of treatment. PCA resulted in two major principal components (PC), PC1 and PC2, on reduction of the dimension of the various PGP parameters. PC1 indicates the highest variance in the dataset with 82.8% (35 days), 84% (70 days), and PC2 shows the lowest variance in the dataset with 14.08% (35 days) and 12% (70 days).

Chili plants were used for a pot experiment to test the effectiveness of the potent endophytic actinobacterial inoculum (VCLA3 and RVRA7) for improving plant growth compared to control plants (**Supplementary Figures 6A–C**). All the endophytic isolates showed significant differences (P<0.05) between the treated plants and the control in terms of plant growth promoting parameters (**Figure 8**). In the treated chili plants, growth indices such as root-shoot length, fresh and dry root-shoot weight were considerably boosted. Fold change analysis revealed an increase in all the vegetative parameters compared to control in both single and consortia treatments, irrespective of the time period with 1.3 to 2.34 fold in the number of leaves, 1.4 to 2.1 fold in the length of the shoot, 1.4 to 2.5 fold in the length of the root, 1.5 to 2.7 fold in the weight of the fresh shoot, 1.5 to 2.78 fold in the weight of the fresh root, 1.6 to 5.3 fold in the weight of the dry shoot and 2.6 to 4.7 fold in the dry root weights (**Supplementary Figure 7**). The increased chlorophyll content in treated plants proves their effect on photosynthesis and growth. We examined the results of 35 and 70 day plant growth enhancement experiments with PCA. The PCA reduced the various PGP parameters to two major components, PC1, and PC2, where PC1 indicates the highest variance in the dataset with 82.8% (35 days), 84% (70 days), and PC2 shows the lowest variance in the dataset with 14.08% (35 days), 12% (70 days) (**Figures 9A, B**).

## Discussion

Large amounts of chemical fertilizers, insecticides, and herbicides used in agriculture to boost crop output and avoid global food shortages have generated environmental problems and are one of the key concerns in agricultural production. To achieve long-term plant development in horticulture and agriculture, researchers are being compelled to explore alternative approaches based on natural sources (Srivastav, 2020). Endophytic actinobacteria are of particular interest because they possess a number of characteristics that stimulate plant growth. Actinobacteria with significant PGP and antimicrobial activities have been discovered in various host plants (Chukwuneme et al., 2020). However, no information is available on the diversity and roles of endophytic actinobacteria associated with epiphytic orchids of North-East India. Here, we have investigated the affiliation of ten epiphytic orchid species with their endophytic actinobacterial microbiota, defined their phylogenetic relationship, and assessed their antifungal and plant growth-promoting characteristics. To our knowledge, no comprehensive research has previously been done on the taxonomic and functional characterization of actinobacteria that live inside the root and leaf tissues of orchids.

51 endophytic actinobacteria were recovered from surface-sterilized root and leaf tissues of ten orchid species of Assam, India, using 7 selective nutrient media. Endophytic actinobacteria are predominant in the host plant’s roots, as a greater number of isolates were found in the roots (n=33; 64.70%) than in the leaves (n=18; 35.29%). Similar findings have been reported by other researchers, with root tissues recovering the highest no of endophytic actinobacteria from other host plants (Kemung et al., 2018; Musa et al., 2020). This might be because roots are the prime organ involved in nutrient and water absorption. Additionally, actinobacteria can get inside the plant when the epidermal layer is disrupted due to the emergence of side roots from the main root (Golinska et al., 2015).

Molecular identification and genetic diversity of all the endophytic actinobacterial isolates were determined by PCR amplification and sequencing of the 16S rRNA genes. The 16S rRNA gene sequences were searched for similarity with EzTaxon and NCBI GenBank database for confirmation and identification of species and analyzed with the help of a phylogenetic tree. The isolates showed 96.65–100% similarity with the reference sequences. Seven isolates showed relatively low similarity with their type strains (96.65-97.90%) in the EzTaxon search. Among the 51 isolates, the majority (n=29, 56.86%) belong to the genus *Streptomyces*. In our study, a few more rare genera have also been identified, including *Nocardiopsis*, *Nocardia*, *Actinomadura*, *Pseudonocardia*, *Nocardioides*, *Microbacterium*, and *Mycolicibacterium*. This indicates that the endophytic actinobacteria in orchid plants are diverse. However, these genera were previously isolated and described as endophytes from various hosts (Shimizu, 2011; Matsumoto and Takahashi, 2017). Endophytic bacteria are species specific and are not distributed randomly throughout the plant organs. Numerous research using diverse plant organs have indicated considerable changes in the composition of the plant endophytic microbiota (Li et al., 2017; Yang et al., 2017). Endophytic actinobacterial species obtained from the roots of *Renanthera imschootiana* were diverse and included *Streptomyces* sp., *Nocardiopsis* sp., *Nocardia* sp., and *Actinomadura* sp., however only culturable *Streptomyces* sp. was obtained from the leaf tissues. Furthermore, only *Streptomyces* sp. was obtained from the roots and leaves of *Dendrobium chrysotoxum*. In *Micropera obtusa* root tissues, only *Nocardioides* sp. was present, while only *Pseudonocardia* sp. was isolated from the leaf tissues of *Rhynochostylis retusa*. This condition shows that orchid plants have an active role in selecting specific bacterial taxa as they progress from below-ground to aerial sections.

Phosphorus is an essential micronutrient for crucial plant functions such as energy transmission, photosynthesis, nutrient movement, sugar, and starch transformation. Despite a large amount of phosphorus in the soil, only a tiny portion of it is readily available to plants since the majority of the phosphorus present is insoluble and exists as mineral salts or organic phosphate, which plants find difficult to absorb. The phosphate solubilizing bacteria (PSB), including actinobacteria, can upsurge the availability of soluble phosphate through various actions. They generate low molecular weight organic acid with hydroxyl and carboxyl groups, which operate as a chelating agent and chelate the cations (mainly Ca) attached to phosphate and transform them into soluble forms. They can also involve enzymatic processes, primarily phosphatase, and phytase, for the solubilization of inorganic phosphate (Boukhatem et al., 2022). Out of the 51 isolates screened in the current investigation, 25 (49%) showed phosphate solubilization activity by solubilizing inorganic tricalcium phosphate in the PKA medium. Total phosphate solubilization falls in the range of 6.7 ± 4.4 -289.7 ± 11.9 µg/ml, similar to the previous findings of Qin et al., 2011. Chand et al., 2020 reported that endophytic fungi associated with *Vanda cristata* solubilize phosphate in the range of 0.194 µg/ml - 0.728 µg/ml. A fall in pH during the growth of phosphate solubilizers in the broth medium containing an insoluble source of P was observed, which was previously reported in other studies. This was shown to be due to the production of low molecular weight organic acids such as gluconic acid, leading to the conclusion that pH and soluble phosphate concentration were inversely proportional (Jog et al., 2014).

Biosynthesis of phytohormones like IAA by beneficial endophytic bacteria is a crucial trait that enables plants to build up a well-structured root system, which increases the plant’s nutrient uptake. In the current study, 27 (52.94%) out of 51 isolates could biosynthesize IAA within the range of 4.0 to 43.8 µg/ml. Production of IAA by endophytic actinobacteria in the presence of l-tryptophan has been well documented in different hosts (Khamna et al., 2010; Goudjal et al., 2013), but in orchids, primarily endophytic bacteria (Tsavkelova et al., 2007a; Tsavkelova et al., 2007b; Faria et al., 2013; Shah et al., 2021) and fungi (Chand et al., 2020) were reported for IAA production. However, A recent study by Tedsree et al., 2022 reported that endophytic actinobacteria associated with Thai orchids produced IAA in the range of 0.04 to 67.30µg/ml.

Some groups of beneficial bacteria can influence biological nitrogen fixation, a process that converts atmospheric nitrogen (N2) to ammonia NH_3_ or NH_4_^+^ (Meena et al., 2017), which plants quickly absorb. Nitrogen is the core component of chlorophyll and plant proteins that build up plant cells and tissues. In the current study, 35 (68.6%) isolates showed ammonia production in peptone water broth with values ranging from 0.9 to 5.9 µmol/ml, which is in accord with the findings of Mei et al., 2021. In qualitative estimation, *Streptomyces* sp. DCLA5 produced the highest ammonia (5.9 µmol/ml). Bacterial ammonia synthesis can accumulate and offer N2 to the plant, improving photosynthetic activity and root, shoots and biomass. Overproduction of ammonia can defend against phytopathogens by reducing pathogen colonization with host plants and inhibiting growth (Swarnalakshmi et al., 2016). Chand et al., 2020 reported the ability of orchid-associated fungi to produce ammonia.

The ability of endophytic actinobacteria to produce siderophores, a chelating agent that can chelate iron from the environment, is a critical characteristic that aids PGP and antagonistic actions. The uptake of iron by plants *via* microbial siderophores, which change iron’s insoluble state into a soluble form, is sufficiently demonstrated by the previous works of Wang et al., 2014; Rangseekaew et al., 2021. Additionally, microbes that produce siderophores may act as a biocontrol agent by biosynthesizing additional antimicrobial substances or by lowering the bioavailability of iron to pathogens. In our study 13 (25.49%), actinobacterial isolates showed siderophore production ability. There have been reports of siderophore production activity from both *Streptomyces* and non-*Streptomyces* genera from a variety of hosts plants, including rice (Gopalakrishnan et al., 2013), wheat (Allali et al., 2019), sorghum, chickpea (Alekhya and Gopalakrishnan, 2016), and medicinal plants (Zhou et al., 2017), etc. Chowdappa et al., 2020 studied the antibacterial properties of siderophores produced by orchid associated endophytic fungi.

In this study, 14 (27.45%) isolates showed ACC deaminase activity. ACC deaminase producing endophytic actinobacteria can induce plant growth by lowering ethylene levels under environmental stress. Ethylene is an essential metabolite for plant development, but under extreme stress conditions, ethylene levels can rise drastically and affect plant development. Endophytic actinobacteria can reduce the stress caused by increased ethylene concentrations by releasing ACC deaminase, which can hydrolyze ACC into ammonia, methionine, and alpha-ketobutyrate to regulate ethylene synthesis (Mulani et al., 2021). Previous literature showed that ACC deaminase-producing endophytic actinobacteria promoted plant growth by reducing ET levels in different hosts (El-Tarabily et al., 2019).

Extracellular enzymes such as cellulase, pectinase, protease, and chitinase produced by microorganisms not only aid in the decomposition of organic matter and stimulation of plant growth but also play a significant part in the prevention of disease by inhibiting soil-borne pathogens (Jog et al., 2016). The production of hydrolytic enzymes is an important trait of endophytic actinobacteria that facilitates plant root colonization and the spread of bacteria inside the plants (Afzal et al., 2019). In our study, out of 51 isolates, 12 (23.5%) isolates were detected as cellulase producers. Most of the endophytic actinobacterial isolates 22 (43.13%) showed protease activity. Chitinase plays a crucial part in the breakdown of crystalline polysaccharides, making them vital enzymes for various applications. They catalyze the hydrolysis of chitin’s -1,4-linkages, which directly impede the growth of hyphal fungal pathogens (Oyeleye and Normi, 2018). In our study, extracellular chitinase activity was detected in 19 (41.1%) isolates.

We checked the antifungal activity of all 51 actinobacterial endophytes against 10 major fungal phytopathogens *in vitro*. 27 (52.94%) isolates confirmed the antagonistic activity against at least one fungal pathogen. Of all examined strains, 19 (37.25%) demonstrated considerable antifungal activity against *Fusarium oxysporum*. In our study, a relatively higher no of endophytic actinobacterial strains exhibited antifungal activity in contrast to Musa et al., 2020, who reported antifungal activity of 54 (42.58%) out of 126 endophytic actinobacteria derived from *Thymus roseus*. The percentage of inhibition among the antifungal actinobacterial strains was in the range of 26.1 ± 0.9 to 92.2 ± 1.2. The highest percentage of inhibition was shown by *Streptomyces* sp. VCRA2, against *F. solani* (92.2 ± 1.2), followed by *Streptomyces* sp. VCLA3 against *P. lamaensis* (91.1 ± 1.7) and *P. hypobrunneae* (90.2 ± 0.7). The majority of the *Streptomyces* strains exhibited antifungal activity. Other genera such as *Actinomadura*, *Nocardia*, *Nocardiopsis*, *Nocardioides*, and *Microbacterium* also showed considerable antifungal activity. Moreover, two isolates *Streptomyces* sp. VCRA2 and *Streptomyces* sp. DNLA13 has demonstrated broad-spectrum antifungal activity against all the tested fungal pathogens. Many *Streptomyces* sp., as well as rare actinobacteria species, were identified as producers of antifungal compounds (Palaniyandi et al., 2013; Musa et al., 2020). *Streptomyces*, have a unique and well-documented ability to create novel antibiotics, and these species have enormous practical significance. Some well-known antibiotics derived from *Streptomyces* have served as fungicides (O’Sullivan et al., 2021). Wang et al., 2013 reported strong biocontrol action of *Streptomyces* against a wide range of phytopathogens. Antimicrobial activity of orchid associated bacteria has been reported against both human and plant pathogens (Wang et al., 2019; Tedsree et al., 2022). These findings suggest that orchid plants may be a one-of-a-kind source of new actinobacteria with the potential to create highly bioactive compounds.

We also screened the potent antifungal strains for the presence of the chitinase gene. A total of 19 (37.25%) strains showed the amplification of 18 family chitinase genes. Detection of chitinase genes in the actinobacterial isolates provides strong evidence that the chitinase enzyme was responsible for the antifungal activity. The presence of these genes indicates the availability of unique mechanisms to stop the growth of fungal phytopathogens. However, additional mechanisms or bioactive substances may be involved in the development of antifungal activity; therefore, the presence or absence of chitinase genes does not exclusively dictate the antagonistic activity of the isolates.

Bonitur scoring based on plant growth and antagonistic traits, two strains *Streptomyces* sp. VCLA3 and *Streptomyces* sp. RVRA7 were identified as the highest ranked among all the other isolates. These isolates were further characterized on the basis of their cultural, physiological and biochemical characteristics. Both the strains were moderately thermotolerant and showed optimal growth at 28°C. The optimal pH for growth of VCLA3 and RVRA7 was pH 9 and pH 7 respectively. In biochemical analysis, both strains could utilize lactose, maltose, fructose, dextrose, galactose, mannitol, arabitol, rhamnose, cellobiose, D-arabinose, and glucose.

SEM analysis of phytopathogen *Fusarium oxysporum* (MTCC-284) treated with VCLA3 confirmed antifungal activity as considerable spore and mycelial morphological alterations were observed in the treatment. Spores were flat and distorted as cytoplasmic structures were washed out from the cells. Our findings are consistent with several studies conducted by other researchers (Kamboj et al., 2017; Chen et al., 2018). A study by Tang-um and Niamsup (2012) reported that *Fusarium oxysporum* f. sp. *lycopersici* affected by *Streptomyces* sp. P4 exhibited cell wall breakdown. The bioactive compounds found in the strain extract might contribute to the morphological modification of the fungus. Leakage of the contents of the cell and cytoplasm coagulation may result from the breakdown of the cell wall, rupture, and damage of the cellular membrane or proteins (Perveen et al., 2022).

Gas chromatography-mass spectrometry (GC-MS) is a reliable analytical instrument for detecting volatile microbial metabolites. Actinobacteria have a great potential for production of volatile organic compounds (VOCs). VOCs mediate a multitude of microbe-microbe interactions, including antagonism. These VOCs can also shape the structure and function of microbial communities (Choudoir et al., 2019). *Streptomyces* species can produce VOCs with antifungal and biocontrol properties. *Streptomyces* have been reported to produce volatile antifungal substances and were studied for their biocontrol efficacy on plant diseases (Palaniyandi et al., 2013). Moore-Landecker and Stotzky (1973) reported that volatile substances from actinomycetes could cause several morphological abnormalities in fungi such as *Aspergillus giganteus*, *F. oxysporum*, *Penicillium viridicatum*, *Trichoderma viride*, and *Zygorhynchus vuilleminii*.

For metabolomics analysis of the microbial crude extract, both liquid chromatography and gas chromatography coupled with a mass detector is mostly used. GC-MS is a powerful analytical tool that combines the separation power of GC with the detection power of MS to produce accurate and efficient data. Because of a well-established library such as the NIST database, the reproducible nature of the technique, robustness, and excellent separation capability, it is one of the most effective, repeatable, and widely used analytical platforms for metabolomic studies (Beale et al., 2018).

Bacteria can produce a diverse range of metabolites from highly polar to non-polar. However, in the current study, ethyl acetate solvent was used to extract the semi-polar to non-polar compounds produced by *Streptomyces* sp. VCLA3 and *Streptomyces* sp. RVRA7 in broth culture. Ethyl acetate is immiscible with the culture broth which allows maximum extraction of semi polar and non-polar compounds from the broth culture. Furthermore, solvents such as methanol and ethanol could be used to extract the polar compounds. These solvents were not used for the current study as they are miscible with culture broth. Moreover, ethyl acetate crude extracts of actinobacterial strains exhibited promising antifungal activity (**Supplementary Figure 2**). Hence, the ethyl acetate crude extracts of the strains *Streptomyces* sp. VCLA3 and *Streptomyces* sp. RVRA7 was analyzed through GC-MS to know the chemical compounds present.

Various chemical compounds were detected at different retention times from both strains. The compounds detected are alkaloids, polymeric aldehyde, phenols, hydrocarbons, and fatty acid esters. In both the *Streptomyces* strains phenol-2,4-bis-(1,1-dimethyl ethyl) was represented as the major chemical compound. The area % of phenol, 2,4-bis-(1,1-dimethylethyl) in VCLA3 and RVRA7 was 17.88 and 26.4 respectively. Phenolic compounds were considered effective antimicrobial agents. They are also free radical eliminators, which can reduce free radicals by donating hydrogen molecules (Yogeswari et al., 2012). As reported by Kumar et al. (2014), the GC-MS fractions with the maximum concentrations of phenolic compounds displayed strong antibacterial activity. Phenol, 2,4-bis-(1,1-dimethyl ethyl), detected in *Streptomyces* sp. CB-75 demonstrated antifungal activity (Chen et al., 2018). However, in VCLA3, phenol 2,6-bis-(1,1-dimethyl ethyl) was also detected. 1-Nonadecene, 1,2-Benzenedicarboxylic acid, bis(2-methyl propyl) ester were detected in both the extracts of VCLA3 and RVRA7. 1-Nonadecene is reported to have antimicrobial, antifungal, antituberculosis, antioxidant and anticancer activity (Samoi et al, 2012);, whereas 1,2-Benzenedicarboxylic acid, bis(2-methyl propyl) ester has strong antioxidant properties (Druzian et al., 2020) and their cytotoxic activity was displayed by a marine-derived *Streptomyces* sp. VITSJK8 (Krishnan et al., 2014). They were also reported to have antimicrobial and biocontrol activity against *Saccharomyces cerevisiae* (Abdel-Kareem et al., 2019). Benzoic acid, 2,6-bis[(trimethylsilyl)oxy]-, trimethylsilyl ester and Benzoic acid, 3-[(2-methoxyethoxy)methoxy] were detected in VCLA3 and RVRA7 respectively, which exhibited antimicrobial activity and also used as preservatives (Kalpana and Rajeswari, 2019).

Cycloheptasiloxane, tetradecamethyl was detected in the *Streptomyces* sp. VCLA3 extract. The antibacterial, immunomodulatory, and antitumor activities of cycloheptasiloxane, teradecamethyl have been well documented (Patil and Jadhav, 2014). It also possess antibacterial, antifungal antioxidant, anti-inflammatory, anti-diabetic, and wound healing properties (Lutfia et al., 2021; Prasathkumar et al., 2021). Cyclononasiloxan octadecamethyl has antifungal activity (Alghamdi and Ababutain, 2019). Kiran et al., 2018 reported about effective biocontrol of multidrug-resistant *Staphylococcus aureus* by antibiotic agent pyrrolo[1,2-a]pyrazine-1,4-dione, hexahydro-3-(2-methylpropyl) extracted from a marine bacteria *Bacillus tequilensis* MS145. Cyclodecasiloxane, eicosamethyl is a bioactive molecule with antirheumatic, hepatoprotective, and anti-spasmodic effects (Prasathkumar et al., 2021).

1-Deoxy-d-mannitol was detected in RVRA7, mannitol act as a compatible solute and hence increases the tolerance of plants to salt and osmotic stress. Mannitol metabolism is implicated in plant responses to pathogen attack, hence it may be involved in plant responses to both biotic and abiotic stresses (Stoop et al., 1996). N-pentadecanol has antibacterial properties (Chatterjee et al., 2018). Ali et al., 2021 discovered n- pentadecanol as one of the primary components in GC-MS analysis of the ethyl acetate extract of *Santolina chamaecyparissus*, which possesses antidiabetic and anticancer properties. Tetracosamethyl-cyclododecasiloxane includes phytochemical, antioxidant, and antimicrobial activity (Al Bratty et al., 2020; Heptasiloxane, hexadecamethyl has phytochemical and antimicrobial properties (Yassin et al., 2021).

Other chemicals found in the GC-MS analysis of VCLA3 and RVRA7 ethyl acetate extracts included Pyrazole-5-carboxylic acid; 1-methyl-3-propyl; 7,9-Di-tert-butyl-1-oxaspiro(4,5)deca-6,9-diene-2,8-dione; Eicosanoic acid, 2-(acetyloxy)-1-[(acetyloxy)methyl]ethyl ester; 1,3,2-Dioxaborinane 2,4-diethyl-5-methyl-6-propyl; Dibutyl phthalate; and 1,3-bis[(2Z)-Hex-2-en-1-yloxy]-1,1,3,3tetramethyl disiloxane. These compounds are not reported to have any biological activity. Though these compounds’ biological activity has not been studied yet, they may have a role in the development of antifungal and plant growth promoting characteristics. The bioactive compounds detected in GC-MS analysis of both stains are well recognized for their antifungal and plant development properties. The major compound phenol, 2,4-bis-(1,1-dimethylethyl) along with other bioactive chemicals identified in both the strains may contribute to the broad-spectrum antifungal activity against an extended range of fungal pathogens. Earlier studies by Sharma and Thakur (2020), Ser et al. (2015), and Tan et al. (2015) demonstrated similar characteristics of metabolites detected through GC-MS.

We have studied the efficacy of two strains, *Streptomyces* sp. VCLA3 and *Streptomyces* sp. RVRA7 for seed germination and *in vivo* plant growth promotion on chili plants. Chili (*Capsicum annuum*) was selected as a test plant for the experiment as they germinate easily. Orchid seeds are difficult to germinate and require a different experimental setup. Moreover, orchid plants are slow growers and require a long period to study the PGP effect. To avoid experimental error and check the beneficial properties of the endophytic actinobacteria within a definite period, the experiment was performed on chili plants. *In vivo* experiment was carried out to determine how well the best strains, VCLA3 and RVRA7 promoted the growth of the test plant. The results revealed a significant (p<0.05) increase in root length, shoot height, fresh and dry weight of chili plants. This study found that the single inoculation of each actinobacterial strain could enhance the growth of chili plants. Maximum growth was exhibited by the consortia of the two actinobacterial strains (*Streptomyces* sp.VCLA3 and *Streptomyces* sp. RVRA7) compared to untreated control in all the vegetative parameters. PCA analysis revealed significant differences between the treated and control chili plants in all growth parameters, which were validated by One-Way ANOVA and fold change analysis. The highest increase was detected in the shoot and root dry weight in all the treatments, with 1.6 to 5.3 and 2.6 to 4.7 fold, respectively. It is well established that endophytic actinobacteria promote growth in various host plants through direct and indirect mechanisms, such as increased stress tolerance, disease prevention, and improved agricultural yields. Many reports have demonstrated the ability of actinobacteria to increase plant biomass by root and shoot elongation. The biological activities of the isolates, such as IAA generation and the uptake of phosphorus, iron, etc., are the major attribute that contributed to the chili plant growth such as height, biomass, and leaf number. Similar findings were reported in the study by Passari et al., 2015, where chili plant growth was enhanced by the inoculation of actinobacteria derived from different medicinal plants. Thilagam and Hemalatha, 2019 also reported that the application of *Streptomyces violaceoruber* fermentation broth significantly increased germination rate (%) total plant height, fresh weight, and chlorophyll content in chili plants, thereby boosting the growth of chili seedlings compared to the control.

## Conclusion

In this study, endophytic actinobacteria residing in ten different epiphytic orchid species of Assam, India have been explored. The majority of the strains demonstrated positive results for *in vitro* extracellular enzyme production, PGP and biocontrol activity against fungal phytopathogens. Two strains that were chosen to assess their *in vivo* PGP efficacy were able to accelerate the growth of chili plants. Since *in vitro* studies are a prerequisite to any nursery or field investigations, these results give persuasive proof that the endophytic actinobacteria living inside orchid plants play a significant role in plant growth promotion. From the PGP experiment, the potentiality of these isolates to promote growth in other plants was also established. However, to establish these PGP strains as bioinoculant or biofertilizers, further study is necessary to assess how they interact with other natural soil microflora, and adopt or colonize into the new soil environment. The orchid associated endophytic actinobacteria with biocontrol and plant growth-promoting properties could be considered promising candidates for sustainable crop production.

## Data availability statement

The datasets presented in this study can be found in online repositories. The names of the repository/repositories and accession number(s) can be found in the article/**Supplementary Material**.

## Author contributions

JS planned, designed, conducted the laboratory experiments, acquired, analyzed the data, interpreted the results, and written and reviewed the original draft. RM assists in manuscript editing. DT conceptualized, supervised, and guided the experimental design of the research work and reviewed and edited the original draft. All authors contributed to the article and approved the submitted version.

## Funding

Institutional core fund, IASST, Guwahati, Assam, India.

## Acknowledgments

We thank the Director, Institute of Advanced Study in Science and Technology (IASST), Guwahati, Assam, India, for providing facilities for this work; the orchid society of eastern Himalaya, regional orchid germplasm conservation and propagation center, Tinsukia-786156, Assam, India for identification and validation of the orchid species, approving, and assisting in sample collection; Bioinformatics Facility (BIF), IASST for providing facilities for data analysis; and Sophisticated Analytical Instrument Centre (SAIC), IASST for providing infrastructure facilities to carry out part of the work. JS wishes to thank the University Grants Commission (UGC), Govt. of India, for offering her the Rajiv Gandhi National Fellowship (F1-17.1/2014-15/RGNF-2014-15-SC-ASS-75533 /(SA-III/Website).

## Conflict of interest

The authors declare that the research was conducted in the absence of any commercial or financial relationships that could be construed as a potential conflict of interest.

## Publisher’s note

All claims expressed in this article are solely those of the authors and do not necessarily represent those of their affiliated organizations, or those of the publisher, the editors and the reviewers. Any product that may be evaluated in this article, or claim that may be made by its manufacturer, is not guaranteed or endorsed by the publisher.

## References

[B1] Abdel-Kareem M. M. Rasmey A. M. Zohri A. A. (2019). The action mechanism and biocontrol potentiality of novel isolates of saccharomyces cerevisiae against the aflatoxigenic aspergillus flavus. Lett. Appl. Microbiol. 68 (2), 104–111. doi: 10.1111/lam.13105 30554415

[B2] Afzal I. Shinwari Z. K. Sikandar S. Shahzad S. (2019). Plant beneficial endophytic bacteria: mechanisms, diversity, host range and genetic determinants. Microbiol. Res. 221, 36–49. doi: 10.1016/j.micres.2019.02.001 30825940

[B3] Ahmad E. Sharma S. K. Sharma P. K. (2020). Deciphering operation of tryptophan-independent pathway in high indole-3-acetic acid (IAA) producing micrococcus aloeverae DCB-20. FEMS Microbiol. Lett. 367, fnaa190. doi: 10.1093/femsle/fnaa190 33201985

[B4] Al Bratty M. Makeen H. A. Alhazmi H. A. Syame S. M. Abdalla A. N. Homeida H. E.. (2020). Phytochemical, cytotoxic, and antimicrobial evaluation of the fruits of miswak plant, salvadora persica l. phytochemical, cytotoxic, and antimicrobial evaluation of the fruits of miswak plant, salvadora persica l. J. Chem. 2020, 4521951. doi: 10.1155/2020/4521951

[B5] Alekhya G. Gopalakrishnan S. (2016). Exploiting plant growth-promoting *Amycolatopsis* sp. in chickpea and sorghum for improving growth and yield. J. Food Legumes. 29, 225–231. doi: 10.1080/03235408.2018.1553472

[B6] Alghamdi A. I. Ababutain I. M. (2019). Research article phytochemical screening and antibacterial activity of eucalyptus camaldulensis’s leaves and bark extracts. Asian. J. Sci. Res. 12 (2), 202–210. doi: 10.3923/ajsr.2019.202.210

[B7] Ali A. Ali A. Warsi M. H. Ahmad W.. (2021). Chemical characterization, antidiabetic and anticancer activities of Santolina chamaecyparissus. Saudi J. Biol. Sci. 28 (8), 4575–4580. doi: 10.1016/j.sjbs.2021.04.060 34354443 PMC8325052

[B8] Alibrandi P. Schnell S. Perotto S. Cardinale M. (2020). Diversity and structure of the endophytic bacterial communities associated with three terrestrial orchid species as revealed by 16S rRNA gene metabarcoding. Front. Microbiol. 11. doi: 10.3389/fmicb.2020.604964 PMC7839077 33519751

[B9] Allali K. Goudjal Y. Zamoum M. Bouznada K. Sabaou N. Zitouni A. (2019). *Nocardiopsis dassonvillei* strain MB22 from the Algerian Sahara promotes wheat seedlings growth and potentially controls the common root rot pathogen bipolaris sorokiniana. J. Plant Pathol. 101, 1115–1125. doi: 10.1007/s42161-019-00347-x

[B10] Beale D. J. Pinu F. R. Kouremenos K. A. Poojary M. M. Narayana V. K. Boughton B. A.. (2018). Review of recent developments in GC–MS approaches to metabolomics-based research. Metabolomics 14, 152. doi: 10.1007/s11306-018-1449-2 30830421

[B11] Bharucha U. Patel K. Trivedi U. B. (2013). Optimization of indole acetic acid production by *Pseudomonas putida* UB1 and its effect as plant growth-promoting rhizobacteria on mustard (*Brassica nigra*). Agric. Res. 2, 215–221. doi: 10.1007/s40003-013-0065-7

[B12] Bhatti S. K. Thakur M. (2022). An overview on orchids and their interaction with endophytes. Bot. Rev., 1–20. doi: 10.1007/s12229-022-09275-5

[B13] Borah A. Thakur D. (2020). Phylogenetic and functional characterization of culturable endophytic actinobacteria associated with camellia spp. for growth promotion in commercial tea cultivars. Front. Microbiol. 11. doi: 10.3389/fmicb.2020.00318 PMC7059647 32180767

[B14] Boukhatem Z. F. Merabet C. Tsaki H. (2022). Plant growth promoting actinobacteria, the most promising candidates as bioinoculants? Front. Agron. 14. doi: 10.3389/gagro.2022.849911

[B15] Cao P. Li C. Wang H. Yu Z. Xu X. Wang X.. (2020). Community structures and antifungal activity of root-associated endophytic actinobacteria in healthy and diseased cucumber plants and streptomyces sp. HAAG3-15 as a promising biocontrol agent. Microorganisms 8 (2), 236. doi: 10.3390/microorganisms8020236 32050670 PMC7074843

[B16] Cappuccino J. G. Sherman N. (2014). “Microbiology,” in A laboratory manual, 10th Edn (London: Pearson).

[B17] Chand K. Shah S. Sharma J. Paudel M. R. Pant B. (2020). Isolation, characterization, and plant growth-promoting activities of endophytic fungi from a wild orchid vanda cristata. Plant Signal. Behav. 15 (5), 1744294. doi: 10.1080/15592324.2020.1744294 32208892 PMC7238887

[B18] Chatterjee S. Karmakar A. Azmi S. A. Barik A. (2018). Antibacterial activity of long-chain primary alcohols from solena amplexicaulis leaves. Proc. Zool. Soc. 71 (4), 313–319. doi: 10.1007/s12595-017-0208-0

[B19] Chen Y. Zhou D. Qi D. Gao Z. Xie J. Luo Y. (2018). Growth promotion and disease suppression ability of a streptomyces sp. CB-75 from banana rhizosphere soil. Front. Microbiol. 8. doi: 10.3389/fmicb.2017.02704 PMC5776099 29387049

[B20] Choudoir M. Rossabi S. Gebert M. Helmig D. Fierer N. (2019). A phylogenetic and functional perspective on volatile organic compound production by actinobacteria. MSystems 4 (2), e00295–e00218. doi: 10.1128/mSystems.00295-18 PMC6401417 30863793

[B21] Chowdappa S. Jagannath S. Konappa N. Udayashankar A. C. Jogaiah S. (2020). Detection and characterization of antibacterial siderophores secreted by endophytic fungi from *Cymbidium aloifolium* . Biomolecules 10 (10), 1412. doi: 10.3390/biom10101412 33036284 PMC7600725

[B22] Christenhusz M. J. Byng J. W. (2016). The number of known plants species in the world and its annual increase. Phytotaxa 261 (3), 201–217. doi: 10.11646/phytotaxa.261.3.1

[B23] Chukwuneme C. F. Babalola O. O. Kutu F. R. Ojuederie O. B. (2020). Characterization of actinomycetes isolates for plant growth promoting traits and their effects on drought tolerance in maize. J. Plant Interact. 15, 93–105. doi: 10.1080/17429145.2020.1752833

[B24] Chun J. Lee J. H. Jung Y. Kim M. Kim S. Kim B. K.. (2007). EzTaxon: a web-based tool for the identification of prokaryotes based on 16S ribosomal RNA gene sequences. Int. J. Syst. Evol. Microbiol. 57, 2259–2261. doi: 10.1099/ijs.0.64915-0 17911292

[B25] Druzian S. P. Pinheiro L. N. Susin N. M. B. Dal Prá V. Mazutti M. A. Kuhn R. C.. (2020). Production of metabolites with antioxidant activity by botryosphaeria dothidea in submerged fermentation. Bioprocess Biosyst. Eng. 43 (1), 13–20. doi: 10.1007/s00449-019-02200-y 31578605

[B26] Dutta J. Gupta S. Thakur D. Handique P. J. (2015). First report of nigrospora leaf blight on tea caused by nigrospora sphaerica in India. Plant Dis. 99, 417. doi: 10.1094/PDIS-05-14-0545-PDN 30699719

[B27] Dworkin M. Foster J. W. (1958). Experiments with some microorganisms which utilize ethane and hydrogen. J. Bacteriol. 75, 592–603. doi: 10.1128/jb.75.5.592-603.1958 13538930 PMC290115

[B28] Elisandra M. Luciana P. M. Cristina S. Thaisa F. Ana E. B. Jos C. G.. (2016). Screening endophytic actinobacteria with potential antifungal activity against bipolaris sorokiniana and growth promotion of wheat seedlings. Afr. J. Microbiol. Res. 10 (36), 1494–1505. doi: 10.5897/AJMR2016.8164

[B29] El-Tarabily K. A. AlKhajeh A. S. Ayyash M. M. Alnuaimi L. H. Sham A. ElBaghdady K. Z.. (2019). Growth promotion of salicornia bigelovii by *Micromonospora chalcea* UAE1, an endophytic 1-aminocyclopropane-1- carboxylic acid deaminase-producing actinobacterial isolate. Front. Microbiol. 10. doi: 10.3389/fmicb.2019.01694 PMC6668420 31396194

[B30] Faria D. C. Dias A. C. F. Melo I. S. de Carvalho Costa F. E. (2013). Endophytic bacteria isolated from orchid and their potential to promote plant growth. World J. Microbiol. Biotechnol. 29 (2), 217–221. doi: 10.1007/s11274-012-1173-4 23014841

[B31] Fiske C. Subbarow Y. (1925). The colorimetric determination of phosphorous. J. Biol. Chem. 66, 375–400. doi: 10.1007/s13398-014-0173-7.2

[B32] Foss S. R. Nakamura C. V. Ueda-Nakamura T. Cortez D. A. Endo E. H. Dias Filho B. P. (2014). Antifungal activity of pomegranate peel extract and isolated compound punicalagin against dermatophytes. Ann. Clin. Microbiol. Antimicrob. 13 (1), 1–6. doi: 10.1186/s12941-014-0032-6 25260038 PMC4353666

[B33] Gangwar M. Rani S. Sharma N. (2012). Investigating endophytic actinomycetes diversity from rice for plant growth promoting and antifungal activity. Int. J. Adv. Life Sci. 1, 12.

[B34] Golinska P. Wypij M. Agarkar G. Rathod D. Dahm H. Rai M. (2015). Endophytic actinobacteria of medicinal plants: diversity and bioactivity. AVL 108, 267–289. doi: 10.1007/s10482-015-0502-7 PMC4491368 26093915

[B35] Gontijo J. B. Andrade G. V. S. Baldotto M. A. Baldotto L. E. B. (2018). Bioprospecting and selection of growth-promoting bacteria for cymbidium sp. orchids. Scientia Agricola 75, 368–374. doi: 10.1590/1678-992x-2017-0117

[B36] Gopalakrishnan S. Srinivas V. Vidya M. S. Rathore A. (2013). Plant growth-promoting activities of *Streptomyces* spp. in sorghum and rice. Springer Plus 2, 1–8. doi: 10.1186/2193-1801-2-574 24255867 PMC3825066

[B37] Gordon S. A. Weber R. P. (1951). Colorimetric estimation of indoleacetic acid. Plant Physiol. 26, 192–195. doi: 10.1104/pp.26.1.192 16654351 PMC437633

[B38] Goudjal Y. Toumatia O. Sabaou N. Barakate M. Mathieu F. Zitouni A. (2013). Endophytic actinomycetes from spontaneous plants of Algerian Sahara: indole-3-acetic acid production and tomato plants growth promoting activity. World J. Microbiol. Biotechnol. 29 (10), 1821–1829. doi: 10.1007/s11274-013-1344-y 23579766

[B39] Herrera H. Sanhueza T. Novotná A. Charles T. C. Arriagada C. (2020). Isolation and identification of endophytic bacteria from mycorrhizal tissues of terrestrial orchids from southern Chile. Diversity 12 (2), 55. doi: 10.3390/d12020055

[B40] Hiscox J. D. Israelstam G. F. (2011). Erratum: a method for the extraction of chlorophyll from leaf tissue without maceration. Can. J. Bot. 57, 1332–1334. doi: 10.1139/b79-163

[B41] Janardhan A. Kumar A. P. Viswanath B. Saigopal D. V. R. Narasimha G. (2014). Production of bioactive compounds by actinomycetes and their antioxidant properties. Biotechnol. Res. Int. 2014, 217030. doi: 10.1155/2014/217030 24790761 PMC3984769

[B42] Jog R. Nareshkumar G. Rajkumar S. (2016). “Enhancing soil health and plant growth promotion by actinomycetes,” in Plant growth promoting actinobacteria (Singapore: Springer), 33–45. doi: 10.1007/978-981-10-0707-1_3

[B43] Jog R. Pandya M. Nareshkumar G. Rajkumar S. (2014). Mechanism of phosphate solubilisation and antifungal activity of *Streptomyces* sp. isolated from wheat roots and rhizosphere and their application in improving plant growth. Microbiology 160, 778–788. doi: 10.1099/mic.0.074146-0 24430493

[B44] Kalpana V. N. Rajeswari V. D. (2019). “Preservatives in beverages: perception and needs,” in Preservatives and preservation approaches in beverages (Cambridge, US: Acad. Press), 1–30. doi: 10.1016/B978-0-12-816685-7.00001-X

[B45] Kamboj P. Gangwar M. Singh N. (2017). Scanning electron microscopy of endophytic actinomycete isolate against *Fusarium oxysporum* for various growth parameters on musk melon. *Int. j. curr. microbiol* . Appl. Sci. 6, 458–464. doi: 10.20546/ijcmas.2017.611.054

[B46] Kasana R. C. Salwan R. Dhar H. Dutt S. Gulati A. (2008). A rapid and easy method for the detection of microbial cellulases on agar plates using gram’s iodine. Curr. Microbiol. 57, 503–507. doi: 10.1007/s00284-008-9276-8 18810533

[B47] Katznelson H. Bose B. (2010). Metabolic activity and phosphate-dissolving capability of bacterial isolates from wheat roots, rhizosphere, and non-rhizosphere soil. Can. J. Microbiol. 5, 79–85. doi: 10.1139/m59-010 13629388

[B48] Kazanas N. (1968). Proteolytic activity of microorganisms isolated from freshwater fish. Appl. Microbiol. 16, 128–132. doi: 10.1128/aem.16.1.128-132.1968 5636454 PMC547330

[B49] Kemung H. M. Tan L. T.-H. Khan T. M. Chan K.-G. Pusparajah P. Goh B.-H.. (2018). Streptomyces as a prominent resource of future anti-MRSA drugs. Front. Microbiol. 9. doi: 10.3389/fmicb.2018.02221 PMC6165876 30319563

[B50] Khamna S. Yokota A. Peberdy J. F. Lumyong S. (2010). Indole-3-acetic acid production by streptomyces sp. isolated from some Thai medicinal plant rhizosphere soils. EurAsia J. Biosci. 4, 23–32. doi: 10.5053/ejobios.2010.4.0.4

[B51] Kiran G. S. Priyadharsini S. Sajayan A. Ravindran A. Selvin J. (2018). An antibiotic agent pyrrolo [1, 2-a] pyrazine-1, 4-dione, hexahydro isolated from a marine bacteria bacillus tequilensis MSI45 effectively controls multi-drug resistant staphylococcus aureus. RSC Adv. 8 (32), 17837–17846. doi: 10.1039/C8RA00820E 35542054 PMC9080480

[B52] Krishnan K. Mani A. Jasmine S. (2014). Cytotoxic activity of bioactive compound 1, 2-benzene dicarboxylic acid, mono 2-ethylhexyl ester extracted from a marine derived streptomyces sp. VITSJK8. Int. J. Mol. Cell. Med. 3 (4), 246. 25635251 PMC4293612

[B53] Kumar P. S. Duraipandiyan V. Ignacimuthu S. (2014). Isolation, screening and partial purification of antimicrobial antibiotics from soil streptomyces sp. SCA 7. kaohsiung. J. Med. Sci. 30, 435–446. doi: 10.1016/j.kjms.2014.05.006 PMC11916805 25224766

[B54] Kumar S. Stecher G. Tamura K. (2016). MEGA7: molecular evolutionary genetics analysis version 7.0 for bigger datasets. Mol. Biol. Evol. 33, 1870–1874. doi: 10.1093/molbev/msw054 27004904 PMC8210823

[B55] Lee Y. S. Kang M. H. Cho S. Y. Jeong C. S. (2007). Effects of constituents of *Amomum xanthioides* on gastritis in rats and on growth of gastric cancer cells. Arch. Pharm. Res. 30, 436–443. doi: 10.1007/BF02980217 17489359

[B56] Liu H. Carvalhais L. C. Crawford M. Singh E. Dennis P. G. Pieterse C. M. J.. (2017). Inner plant values: diversity, colonization and benefits from endophytic bacteria. Front. Microbiol. 8. doi: 10.3389/fmicb.2017.02552 PMC5742157 29312235

[B57] Li O. Xiao R. Sun L. Guan C. Kong D. Hu X. (2017). Bacterial and diazotrophic diversities of endophytes in dendrobium catenatum determined through barcoded pyrosequencing. PLoS One 12, e0184717. doi: 10.1371/journal.pone.0184717 28931073 PMC5607135

[B58] López S. M. Y. Pastorino G. N. Fernández-González A. J. Franco M. E. E. Fernández-López M. Balatti P. A. (2020). The endosphere bacteriome of diseased and healthy tomato plants. Arch. Microbiol. 202 (10), 2629–2642. doi: 10.1007/s00203-020-01987-9 32710156

[B59] Lutfia A. Munir E. Yurnaliza Y. Basyuni M. (2021). Chemical analysis and anticancer activity of sesterterpenoid from an endophytic fungus hypomontagnella monticulosa Zg15SU and its host zingiber griffithii baker. Heliyon 7 (2), e06292. doi: 10.1016/j.heliyon.2021.e06292 33665446 PMC7900702

[B60] Matsumoto A. Takahashi Y. (2017). Endophytic actinomycetes: promising source of novel bioactive compounds. J. Antibiot. 70, 514–519. doi: 10.1038/ja 28270688

[B61] McCormick M. K. Whigham D. F. Canchani-Viruet A. (2018). Mycorrhizal fungi affect orchid distribution and population dynamics. New Phytol. 219 (4), 1207–1215. doi: 10.1111/nph.15223 29790578

[B62] Meena M. Swapnil P. Zehra A. Aamir M. Dubey M. Goutam J.. (2017). “Beneficial microbes for disease suppression and plant growth promotion,” in Plant-microbe interactions in agro-ecological perspectives. Eds. Singh D. P. Singh H. B. Prabha R. (Singapore: Springer). doi: 10.1007/978-981-10-6593-4_16

[B63] Mei C. Chretien R. L. Amaradasa B. S. He Y. Turner A. Lowman S. (2021). Characterization of phosphate solubilizing bacterial endophytes and plant growth promotion *in vitro* and in greenhouse. Microorganisms 9 (9), 1935. doi: 10.3390/microorganisms9091935 34576829 PMC8469958

[B64] Mitra D. Mondal R. Khoshru B. Senapati A. Radha T. K. Mahakur B.. (2022). Actinobacteria-enhanced plant growth, nutrient acquisition, and crop protection: Advances in soil, plant, and microbial multifactorial interactions. Pedosphere 32 (1), 149–170. doi: 10.1016/S1002-0160(21)60042-5

[B65] Mohamad O. A. A. Liu Y. H. Huang Y. Li L. Ma J. B. Egamberdieva D.. (2022). The metabolic potential of endophytic actinobacteria associated with medicinal plant thymus roseus as a plant-growth stimulator. Microorganisms 10 (9), 1802. doi: 10.3390/microorganisms1009182 36144404 PMC9505248

[B66] Moore-Landecker E. Stotzky G. (1973). Morphological abnormalities of fungi induced by volatile microbial metabolites. Mycologia 65 (3), 519–530. doi: 10.1080/00275514.1973.12019467 4199221

[B67] Mulani R. Mehta K. Saraf M. Goswami D. (2021). Decoding the mojo of plant-growth-promoting microbiomes. Physiolo. Mol. Plant Pathol. 115, 101687. doi: 10.1016/j.pmpp.2021.101687

[B68] Musa Z. Ma J. Egamberdieva D. Abdelshafy Mohamad O. A. Abaydulla G. Liu Y.. (2020). Diversity and antimicrobial potential of cultivable endophytic actinobacteria associated with the medicinal plant thymus roseus. Front. Microbiol. 11. doi: 10.3389/fmicb.2020.00191 PMC7080825 32226412

[B69] Naik P. R. Sahoo N. Goswami D. Ayyadurai N. Sakthivel N. (2008). Genetic and functional diversity among fluorescent *Pseudomonas* isolated from the rhizosphere of banana. Microb. Ecol. 56, 492–504. doi: 10.1007/s00248-008-9368-9 18347847

[B70] Narsing Rao M. P. Li W. J. (2022). “Diversity of actinobacteria in various habitats,” in Actinobacteria (Singapore: Springer), 37–58. doi: 10.1007/978-981-16-5835-8_2

[B71] Oberhofer M. Hess J. Leutgeb M. Gössnitzer F. Rattei T. Wawrosch C.. (2019). Exploring actinobacteria associated with rhizosphere and endosphere of the native alpine medicinal plant leontopodium nivale subspecies alpinum. Front. Microbiol. 10. doi: 10.3389/fmicb.2019.02531 PMC6857621 31781058

[B72] Onofre-Lemus J. Hernández-Lucas I. Girard L. Caballero-Mellado J.. (2009). ACC (1-aminocyclopropane-1-carboxylate) deaminase activity, a widespread trait in Burkholderia species, and its growth-promoting effect on tomato plants. Appl. Environ. Microbiol. 75, 6581–6590. doi: 10.1128/AEM.01240-09 19700546 PMC2765135

[B73] O’Sullivan C. A. Roper M. M. Myers C. A. Thatcher L. F. (2021). Developing actinobacterial endophytes as biocontrol products for fusarium pseudograminearum in wheat. Front. Bioeng 555. doi: 10.3389/fbioe.2021.691770 PMC8276002 34268299

[B74] Oyeleye A. Normi Y. M. (2018). Chitinase: diversity, limitations, and trends in engineering for suitable applications. Biosci. Rep. 38 (4), BSR2018032300. doi: 10.1042/BSR20180323 30042170 PMC6131217

[B75] Palaniyandi S. A. Yang S. H. Zhang L. Suh J.-W. (2013). Effects of actinobacteria on plant disease suppression and growth promotion. Appl. Microbiol. Biotechnol. 97, 9621–9636. doi: 10.1007/s00253-013-5206-1 24092003

[B76] Pal R. B. Gokarn K. (2010). Siderophores and pathogenecity of microorganisms. J. Biosci. Technol. 1, 127–134.

[B77] Pant B. Raskoti B. B. (2013). Medicinal orchids of Nepal (Nepal: Himalayan map house), 104.

[B78] Passari A. K. Mishra V. K. Gupta V. K. Yadav M. K. Saikia R. Singh B. P.. (2015). In vitro and in vivo plant growth promoting activities and DNA fingerprinting of antagonistic endophytic actinomycetes associates with medicinal plants. PLoS One 10, e0139468. doi: 10.1371/journal.pone.0139468 26422789 PMC4589368

[B79] Patel J. K. Madaan S. Archana G. (2018). Antibiotic producing endophytic streptomyces spp. colonize above-ground plant parts and promote shoot growth in multiple healthy and pathogen-challenged cereal crops. Microbiol. Res. 215, 36–45. doi: 10.1016/j.micres.2018.06.003 30172307

[B80] Patil A. Jadhav V. (2014). GC-MS analysis of bioactive components from methanol leaf extract of toddaliaasiatica (L.). Int. J. Pharm. Sci. Rev. Res. 29 (1), 18–20.

[B81] Perveen K. Bukhari N. A. Al Masoudi L. M. Alqahtani A. N. Alruways M. W. Alkhattaf F. S. (2022). Antifungal potential, chemical composition of chlorella vulgaris and SEM analysis of morphological changes in *Fusarium oxysporum* . Saudi J. Biol. Sci. 29 (4), 2501–2505. doi: 10.1016/j.sjbs.2021.12.033 35531239 PMC9073035

[B82] Pradhan S. Dubey R. C.. (2021). GC–MS analysis and molecular docking of bioactive compounds of Camellia sinensis and Camellia assamica. Arch. Microbiol. 203 (5), 2501–2510. doi: 10.1007/s00203-021-02209-6 33677633

[B83] Prasathkumar M. Raja K. Vasanth K. Khusro A. Sadhasivam S. Sahibzada M. U. K.. (2021). Phytochemical screening and *in vitro* antibacterial, antioxidant, anti-inflammatory, anti-diabetic, and wound healing attributes of senna auriculata (L.) roxb. leaves. Arab. J. Chem. 14 (9), 103345. doi: 10.1590/1519-6984.182959

[B84] Qin S. Xing K. Jiang J. H. Xu L. H. Li W. J. (2011). Biodiversity, bioactive natural products and biotechnological potential of plant-associated endophytic actinobacteria. appl. Microbiol. Biotechnol. 89, 457–473. doi: 10.1007/s00253-010-2923-6 20941490

[B85] Ranganathan K. Thavaranjit A. C. (2015). Promotion of vegetable seed germination by soil borne bacteria. Arch. Appl. Sci. Res. 7 (8), 17–20. doi: 10.1007/s13213-010-0117-1

[B86] Rangseekaew P. Barros-Rodríguez A. Pathom-Aree W. Manzanera M. (2021). Deep-sea actinobacteria mitigate salinity stress in tomato seedlings and their biosafety testing. Plants 8, 1687. doi: 10.3390/plants10081687 PMC8401925 34451732

[B87] Rukachaisirikul T. Siriwattanakit P. Sukcharoenphol K. Wongvein C. Ruttanaweang P. Wongwattanavuch P.. (2004). Chemical constituents and bioactivity of *Piper sarmentosum* . J. Ethnopharmacol. 1, 93(2–3):173-6. doi: 10.1016/j.jep.2004.01.022 15234750

[B88] Salwan R. Sharma V. (2020). Molecular and biotechnological aspects of secondary metabolites in actinobacteria. Microbiol. Res. 231, 126374. doi: 10.1016/j.micres.2019.126374 31756597

[B89] Schulz B. Wanke U. Draeger S. Aust H. J. (1993). Endophytes from herbaceous plants and shrubs: effectiveness of surface sterilization methods. Mycol. Res. 97, 1447–1450. doi: 10.1016/S0953-7562(09)80215-3

[B90] Schwyn B. Neilands J. B. (1987). Universal chemical assay for the detection and determination of siderophores. Anal. Biochem. 160, 47–56. doi: 10.1016/0003-2697(87)90612-9 2952030

[B91] Ser H. L. Palanisamy U. D. Yin W. F. Abd Malek S. N. Chan K. G. Goh B. H. (2015). Presence of antioxidative agent, Pyrrolo [1, 2-a] pyrazine-1, 4-dione, hexahydro-in newly isolated Streptomyces mangrovisoli sp. nov. Front. Microbiol 6, 854. doi: 10.3389/fmicb.2015.00854 26347733 PMC4542459

[B92] Shah S. Chand K. Rekadwad B. Shouche Y. S. Sharma J. Pant B. (2021). A prospectus of plant growth promoting endophytic bacterium from orchid (Vanda cristata). BMC Biotechnol. 21 (1), 1–9. doi: 10.1186/s12896-021-00676-9 33618710 PMC7901085

[B93] Sharma P. Thakur D. (2020). Antimicrobial biosynthetic potential and diversity of culturable soil actinobacteria from forest ecosystems of northeast India. Sci. Rep. 10, 1–18. doi: 10.1038/s41598-020-60968-6 32139731 PMC7057963

[B94] Shimizu M. (2011). “Endophytic actinomycetes: biocontrol agents and growth promoters,” in Bacteria in agrobiology: Plant growth responses. Ed. Maheshwari D. (Berlin: Springer), 201–220. doi: 10.1007/978-3-642-20332-9_10

[B95] Shirling E. B. Gottlieb D. (1966). Methods for characterization of streptomyces species. Int. J. Syst. Bacteriol. 16, 313–340. doi: 10.1099/00207713-16-3-313

[B96] Singh R. Dubey A. K. (2018). Diversity and applications of endophytic actinobacteria of plants in special and other ecological niches. Front. Microbiol. 9, 1767. doi: 10.3389/fmicb.2018.01767 30135681 PMC6092505

[B97] Skujins J. J. Potgieter H. J. Alexander M. (1965). Dissolution of fungal cell walls by a *Streptomycete* chitinase and beta-(1–3) glucanase. Arch. Biochem. Biophys. 111, 358–364. doi: 10.1016/0003-9861(65)90197-9 5861997

[B98] Smaoui S. Mathieu F. Elleuch L. Coppel Y. Merlina G. Karray-Rebai I.. (2012). Taxonomy, purification and chemical characterization of four bioactive compounds from new streptomyces sp. TN256 strain. World J. Microbiol. Biotechnol. 28 (3), 793–804. doi: 10.1007/s11274-011-0872-6 22805798

[B99] Srivastav A. L. (2020). “Chemical fertilizers and pesticides: role in groundwater contamination,” in Agrochemicals detection, treatment and remediation (Oxford, UK: Butterworth-Heinemann), 143–159. doi: 10.1016/B978-0-08-103017-2.00006-4

[B100] Stoop J. M. Williamson J. D. Pharr D. M. (1996). Mannitol metabolism in plants: a method for coping with stress. Trends Plant Sci. 1 (5), 139–144. doi: 10.1016/S1360-1385(96)80048-3

[B101] Suárez J. P. KottKe I. (2016). Main fungal partners and different levels of specificity of orchid mycorrhizae in the tropical mountain forests of Ecuador. Lankesteriana 16 (2), 299–305. doi: 10.15517/lank.v16i2.26014

[B102] Sun W. Zhang F. He L. Karthik L. Li Z. (2015). Actinomycetes fromthe south China Sea sponges: isolation, diversity, and potential for aromaticpolyketides discovery. Front. Microbiol. 6. doi: 10.3389/fmicb.2015.01048 PMC4589764 26483773

[B103] Supaphon P. Phongpaichit S. Rukachaisirikul V. Sakayaroj J. (2013). Antimicrobial potential of endophytic fungi derived from three seagrass species: *Cymodocea serrulata*, *Halophila ovalis* and *Thalassia hemprichii* . PLoS One 8, e72520. doi: 10.1371/journal.pone.0072520 23977310 PMC3745589

[B104] Swarnalakshmi K. Senthilkumar M. Ramakrishnan B. (2016). “Endophytic actinobacteria: nitrogen fixation, phytohormone production, and antibiosis,” in Plant growth promoting actinobacteria (Singapore: Springer), 123–145. doi: 10.1007/978-981-10-0707-1_8

[B105] Tamura K. Nei M. (1993). Estimation of the number of nucleotide substitutions in the control region of mitochondrial DNA in humans and chimpanzees. Mol. Biol. Evol. 10 (3), 512–526. doi: 10.1093/oxfordjournals.molbev.a040023 8336541

[B106] Tang-um J. Niamsup H. (2012). Chitinase production and antifungal potential of endophytic *Streptomyces* strain P4. Maejo. Int. J. Sci. Technol. 6, 95–104. doi: 10.14456/mijst.2012.8

[B107] Tan L. Ser H. Yin W. Chan K. Lee L. Goh B. (2015). Investigation of antioxidative and anticancer potentials of streptomyces sp. MUM256 isolated from Malaysia mangrove soil. Front. Microbiol. 6. doi: 10.3389/fmicb.2015.01316 PMC4659911 26635777

[B108] Tedsree N. Likhitwitayawuid K. Sritularak B. Tanasupawat S. (2022). Diversity and antimicrobial activity of plant growth promoting endophytic actinomycetes isolated from Thai orchids. Environ. Nat. Resour. J 20(4), 379–392. doi: 10.32526/ennrj/20/202200039.

[B109] Thilagam R. Hemalatha N. (2019). Plant growth promotion and chilli anthracnose disease suppression ability of rhizosphere soil actinobacteria. J. Appl. Microbiol. 126 (6), 1835–1849. doi: 10.1111/jam.14259 30901131

[B110] Trivedi P. Leach J. E. Tringe S. G. Sa T. Singh B. K. (2020). Plant–microbiome interactions: from community assembly to plant health. Nat. Rev. Microbiol. 18 (11), 607–621. doi: 10.1038/s41579-020-0412-1 32788714

[B111] Tsavkelova E. A. Cherdyntseva T. A. Botina S. G. Netrusov A. I. (2007a). Bacteria associated with orchid roots and microbial production of auxin. Microbiol. Res. 162 (1), 69–76. doi: 10.1016/j.micres.2006.07.014 17140781

[B112] Tsavkelova E. A. Cherdyntseva T. A. Klimova S. Y. Shestakov A. I. Botina S. G. Netrusov A. I. (2007b). Orchid-associated bacteria produce indole-3-acetic acid, promote seed germination, and increase their microbial yield in response to exogenous auxin. Arch. Microbiol. 188 (6), 655–664. doi: 10.1007/s00203-007-0286-x 17687544

[B113] Velmourougane K. Saxena G. Prasanna R. (2017). “Plant-microbe interactions in the rhizosphere: mechanisms and their ecological benefits,” in Plant-microbe interactions in agro-ecological perspectives (Singapore: Springer), 193–219. doi: 10.1007/978-981-10-6593-4_7

[B114] Wang S. S. Liu J. M. Sun J. Sun Y. F. Liu J. N. Jia N.. (2019). Diversity of culture-independent bacteria and antimicrobial activity of culturable endophytic bacteria isolated from different dendrobium stems. Sci. Rep. 9 (1), 1–12. doi: 10.1038/s41598-019-46863-9 31316117 PMC6637234

[B115] Wang W. Qiu Z. Tan H. Cao L. (2014). Siderophore production by actinobacteria. Biometals 27 (4), 623–631. doi: 10.1007/s10534-014-9739-2 24770987

[B116] Wang C. Wang Z. Qiao X. Li Z. Li F. Chen M.. (2013). Antifungal activity of volatile organic compounds from streptomyces alboflavus TD-1. FEMS Microbiol. Lett. 341, 45–51. doi: 10.1111/1574-6968.12088 23351181

[B117] Weisburg W. G. Barns S. M. Pelletier D. A. Lane D. J. (1991). 16S ribosomal DNA amplification for phylogenetic study. J. Bacteriol. Res. 173 (2), 697–703. doi: 10.1128/jb.173.2.697-703.1991 PMC207061 1987160

[B118] Williamson N. Brian P. Wellington E. M. H. (2000). Molecular detection of bacterial and. Streptomycete chitinases environ. AVL. 78 (3), 315–321. doi: 10.1023/A:1010225909148 11386354

[B119] Xu T. Vo Q. A. Barnett S. J. Ballard R. A. Zhu Y. Franco C. M. (2022). Revealing the underlying mechanisms mediated by endophytic actinobacteria to enhance the rhizobia-chickpea (Cicer arietinum l.) symbiosis. Plant Soil 474, 299–318. doi: 10.1007/s11104-022-05335-2

[B120] Yang R. Liu P. Ye W. (2017). Illumina-based analysis of endophytic bacterial diversity of tree peony (Paeonia sect. moutan) roots and leaves. braz. J. Microbiol. 48, 695–705. doi: 10.1016/j.bjm.2017.02.009 PMC5628320 28606427

[B121] Yassin M. T. Mostafa A. A. F. Al Askar A. A. (2021). *In vitro* evaluation of biological activities and phytochemical analysis of different solvent extracts of punica granatum L.(Pomegranate) peels. Plants 10 (12), 2742. doi: 10.3390/plants10122742 34961213 PMC8709468

[B122] Yogeswari S. Ramalakshmi S. Neelavathy R. Muthumary J. (2012). Identification and comparative studies of different volatile fractions from monochaetia kansensis by GCMS. Global J. Pharmacol. 6, 65–71.

[B123] Yoolon S. Kruasuwan W. Pham H. Jaemsaeng R. Jantasuriyarat C. Thamchaipenet A. (2019). Modulation of salt tolerance in Thai jasmine rice (Oryza sativa l. cv. KDML105) by *Streptomyces venezuelae* ATCC 10712 expressing ACC deaminase. Sci. Rep. 9, 1275. doi: 10.1038/s41598-018-37987-5 30718781 PMC6361907

[B124] Zhang Y. Yu X. Zhang W. Lang D. Zhang X. Cui G.. (2019). Interactions between endophytes and plants: beneficial effect of endophytes to ameliorate biotic and abiotic stresses in plants. J. Plant Biol. 62, 1–13. doi: 10.1007/s12374-018-0274-5

[B125] Zhao K. Li J. Shen M. Chen Q. Liu M. Ao X.. (2018a). Actinobacteria associated with chinaberry tree are diverse and show antimicrobial activity. Sci. Rep. 8 (1), 1–10. doi: 10.1038/s41598-018-29442-2 30038421 PMC6056502

[B126] Zhao K. Li J. Zhang X. Chen Q. Liu M. Ao X.. (2018b). Actinobacteria associated with glycyrrhiza inflata bat. are diverse and have plant growth promoting and antimicrobial activity. Sci. Rep. 8, 13661. doi: 10.1038/s41598-018-32097-8 30209357 PMC6135863

[B127] Zhou N. Zhao S. Tian C.-Y. (2017). Effect of halotolerant rhizobacteria isolated from halophytes on the growth of sugar beet (Beta vulgaris l.) under salt stress. FEMS Microbiol. Lett. 364, 11. doi: 10.1093/femsle/fnx091 28460054

