# Supplementary material for: Phylogenetic affiliation of endophytic actinobacteria associated with selected orchid species and their role in growth promotion and suppression of phytopathogens
Source: Front Plant Sci. 2022 Dec 7;13:1058867. doi: 10.3389/fpls.2022.1058867 (PMC9769409; doi:10.3389/fpls.2022.1058867)
Supplement: Supplementary file 1 [file DataSheet_1.docx]

**Supplementary Figure 1.** Plant growth promoting characterization of endophytic actinobacterial strains associated with Orchid. A. Chitinase enzyme production. B. Phosphate solubilization. C. Protease activity. D.IAA production. E. Ammonia production and F. Hi-Carbohydrate test


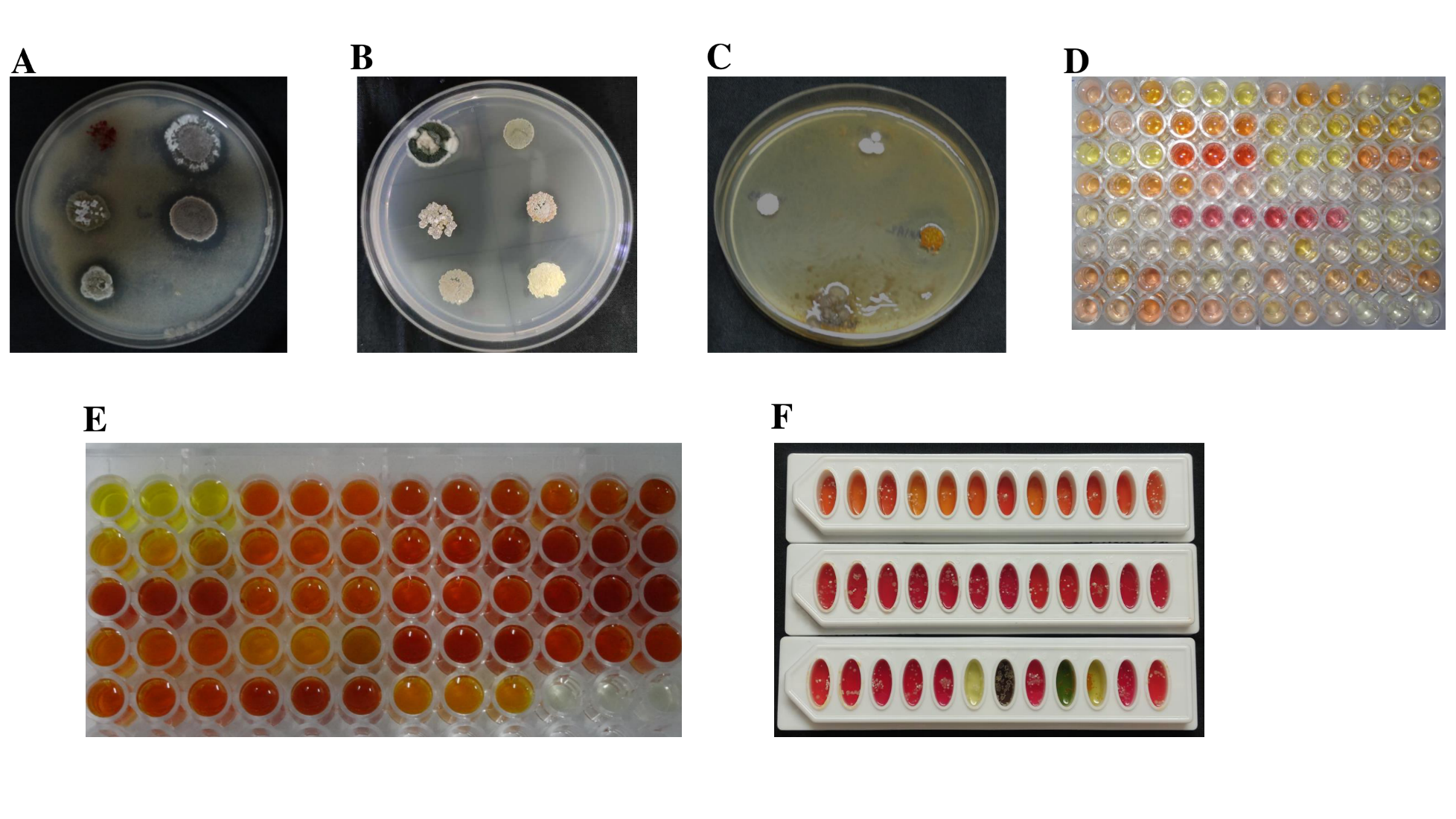


**Supplementary Figure 2** In-vitro antifungal activity of orchid associated endophytic actinobacteria against 1. *Poria hypobrunnea* (ITCC 4141) , and 2. *Colletotrichum capsici* (MTCC-8473) in PDA media (Potato dextrose agar) (A) amphotericin B (positive control) (B) 10% DMSO (negative control) and (C) Ethyl acetate actinobacterial crude extract.


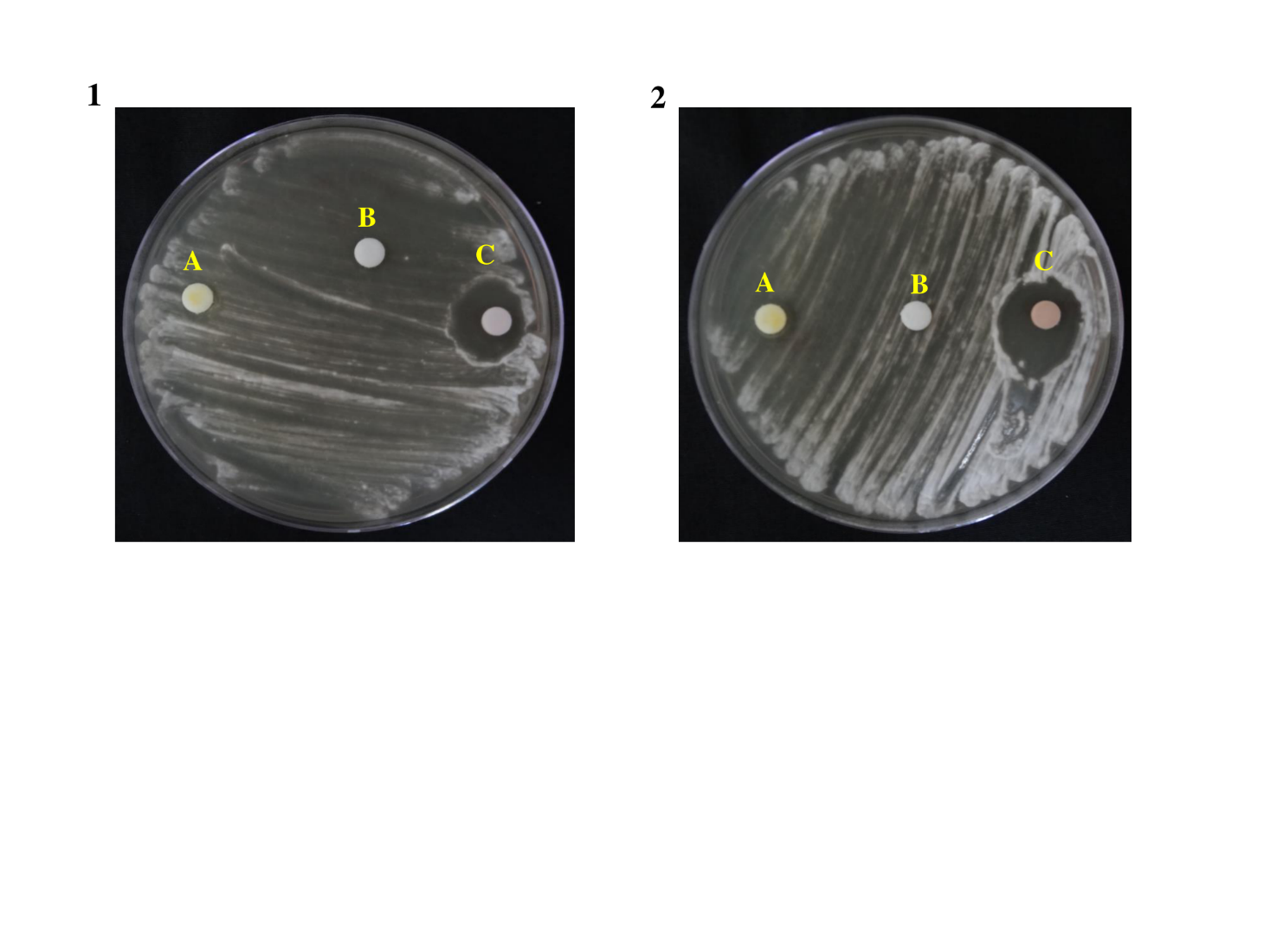


**Supplementary Figure 3** PCR amplification and detection of chitinase (glycoside hydrolase 18 family chitinase gene) gene (400bp) using GAIF and GAIR primers in potent endophytic actinobacterial strains.


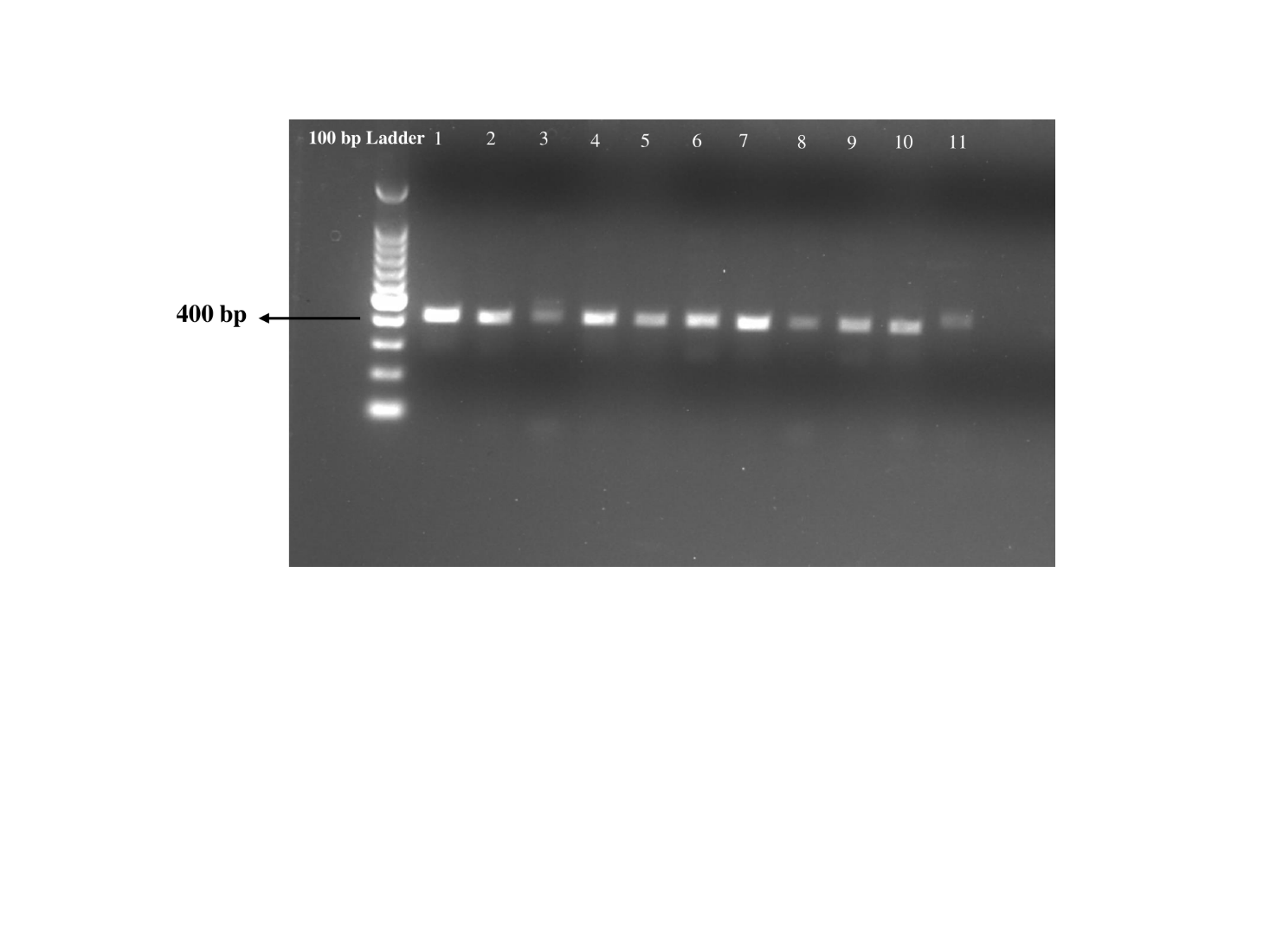


**Supplementary Figure 4** Cultural and physiological characteristics of **A-B.** VCLA3 and **C-D** RVRA7


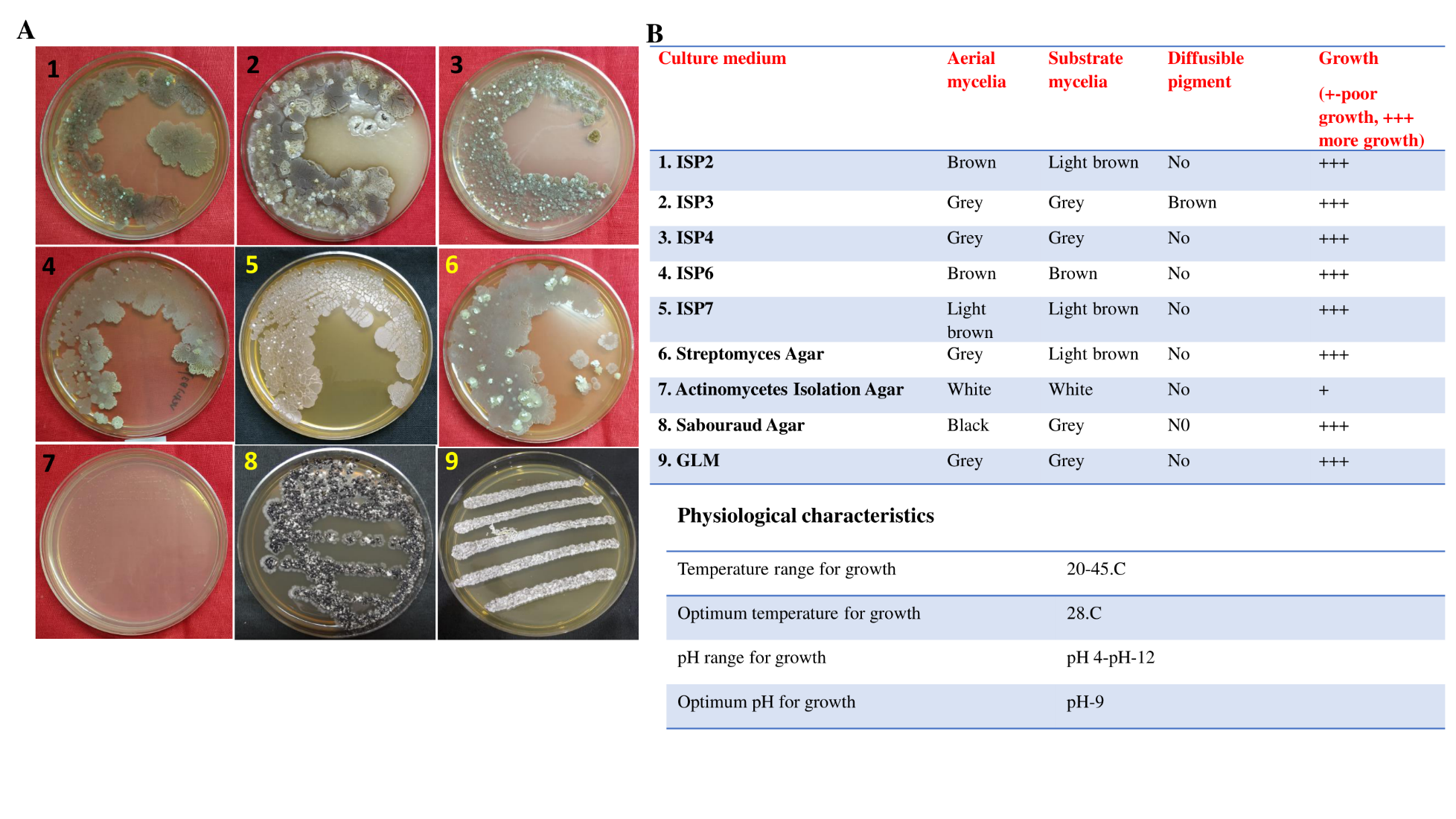


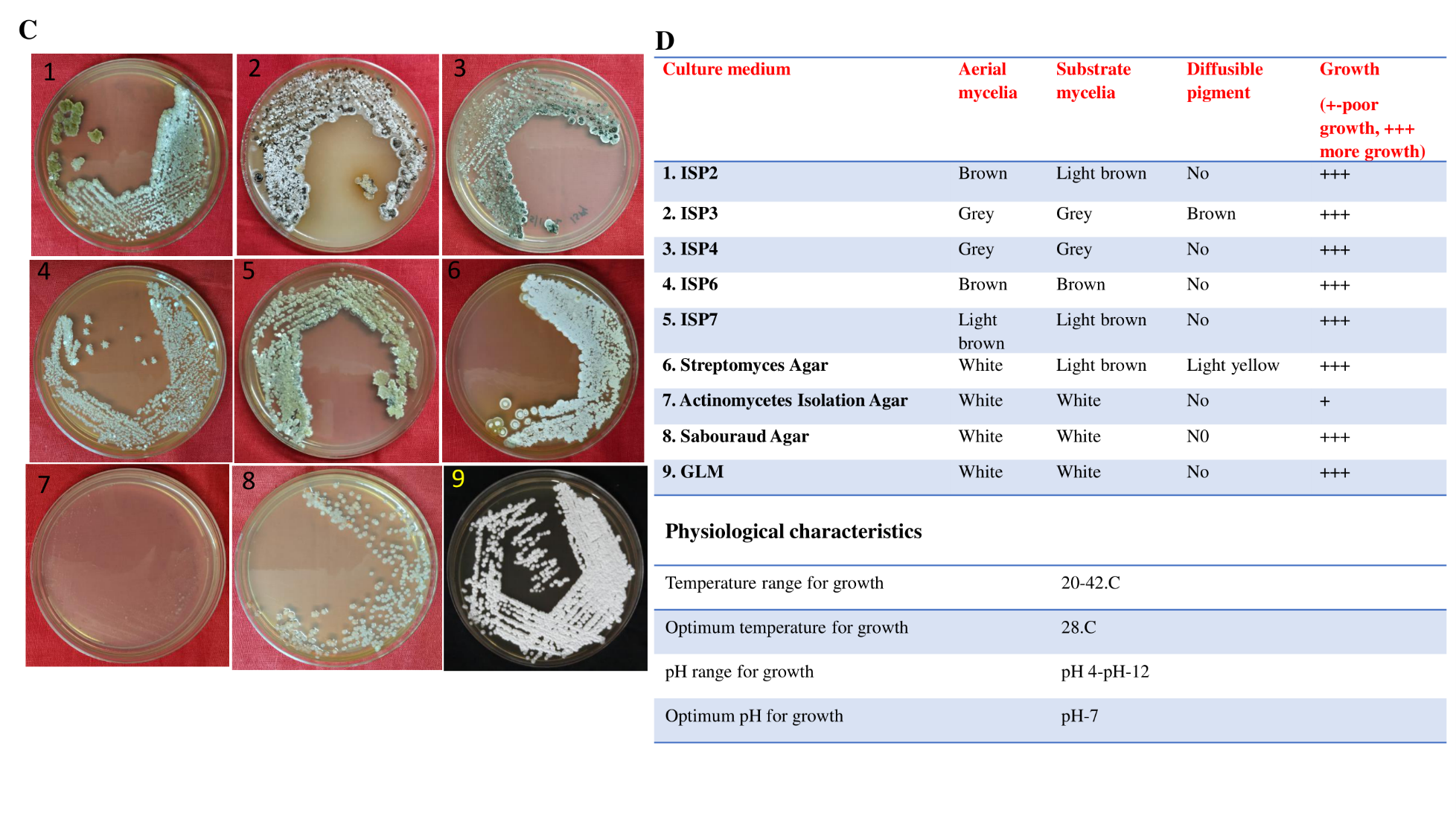


**Supplementary Figure 5** Scanning electron micrograph showing spore chain morphology of *Streptomyces* sp. **(A)**VCLA3 and **(B)** RVRA7


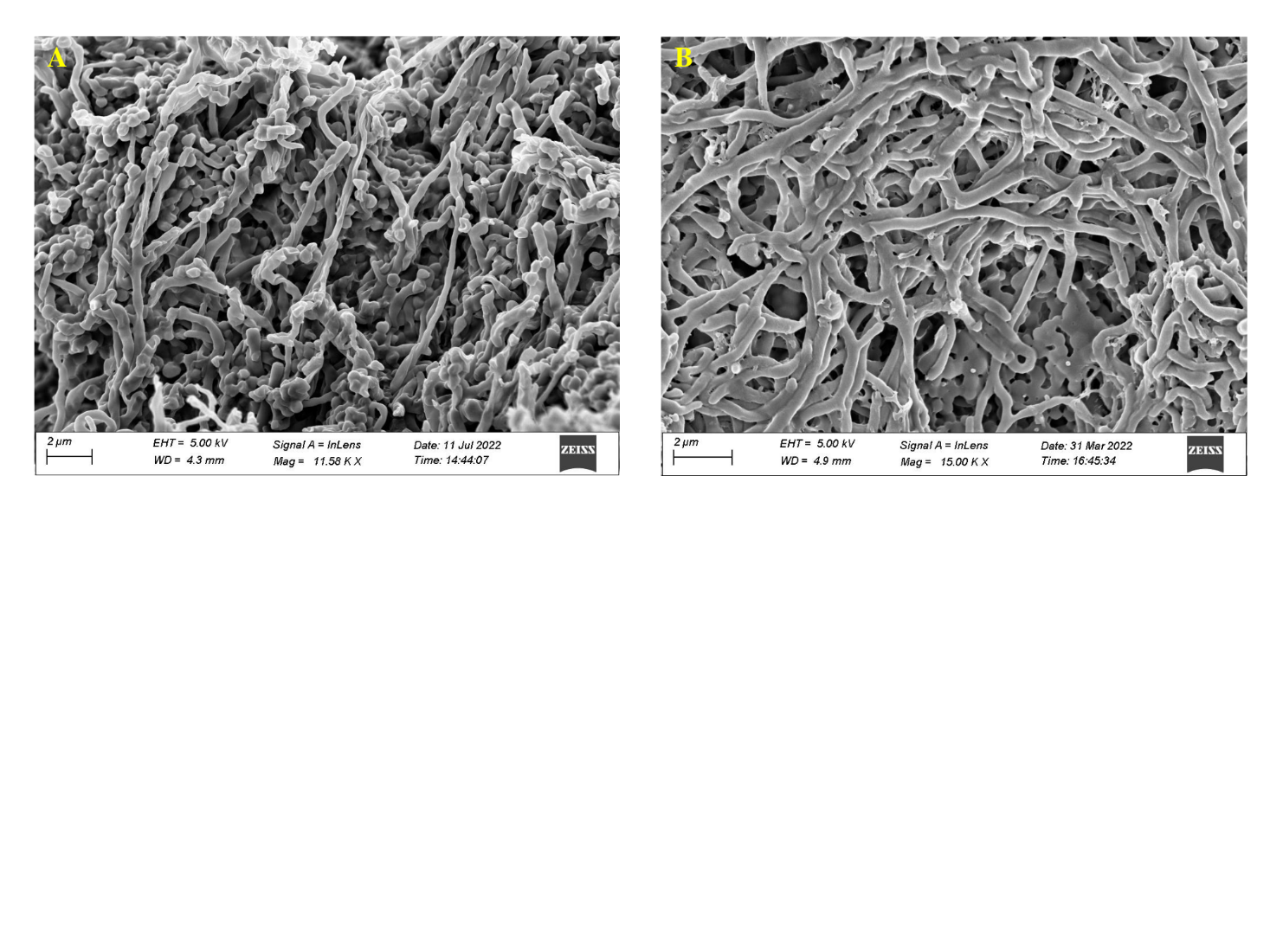


**Supplementary Figure 6** *In vivo* plant growth promotion experiment. **(A)** plant growth promotion experiment on pots, **(B)** Root of Inoculated chilli plant vs control **(C)** Endophytic actinobacteria treated chilli plant vs uninoculated control.
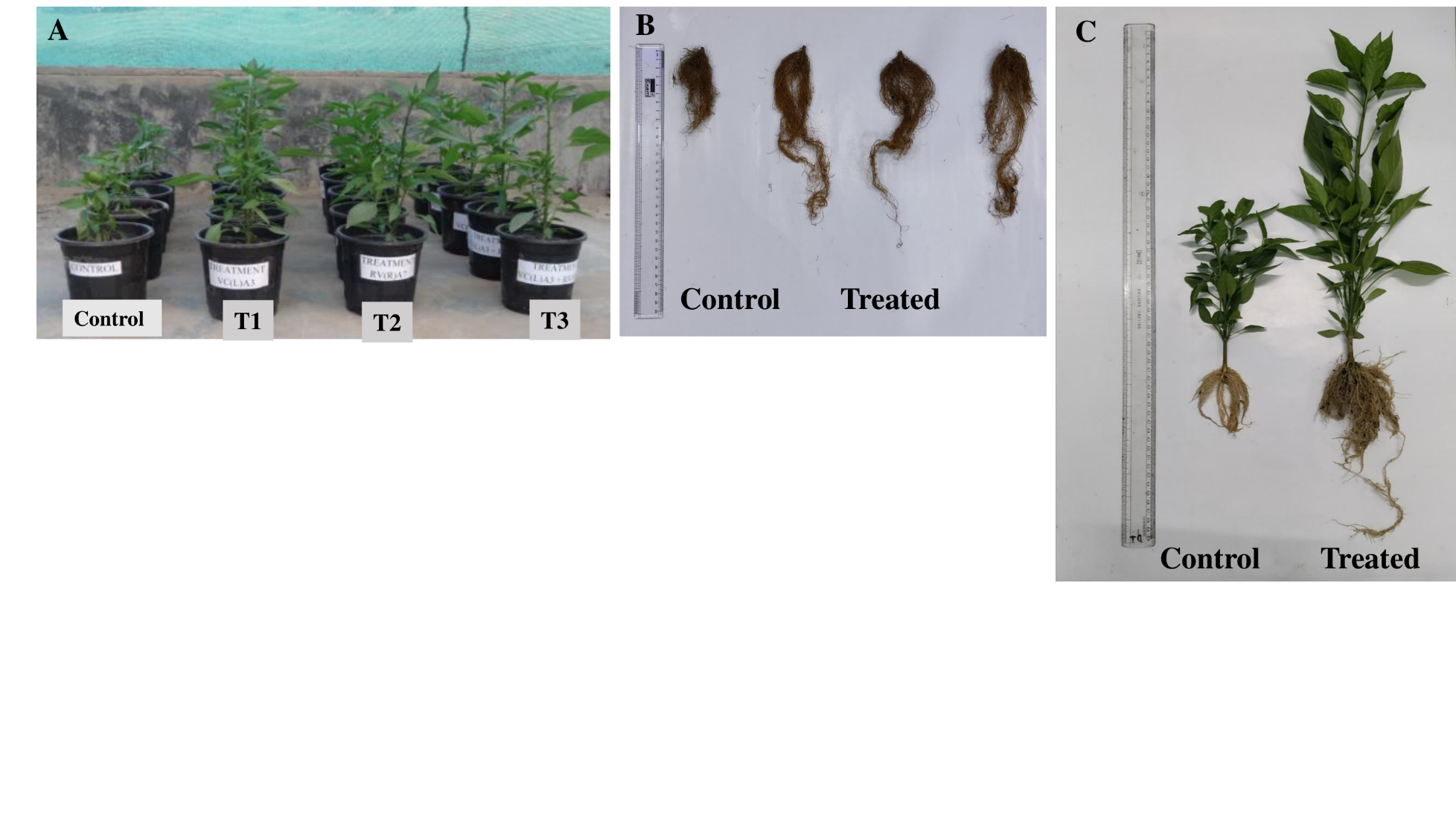


**Supplementary figure 7:** Fold change analysis of chilli plant treated with endophytic actinobacterial strains**. 3 A, B & C-35 days treatment; 3 D, E, & F-70 days Treatment. Treatment 1(VCLA3), Treatment 2 (RVRA7), Treatment 3 (VCLA3+ RVRA7).**


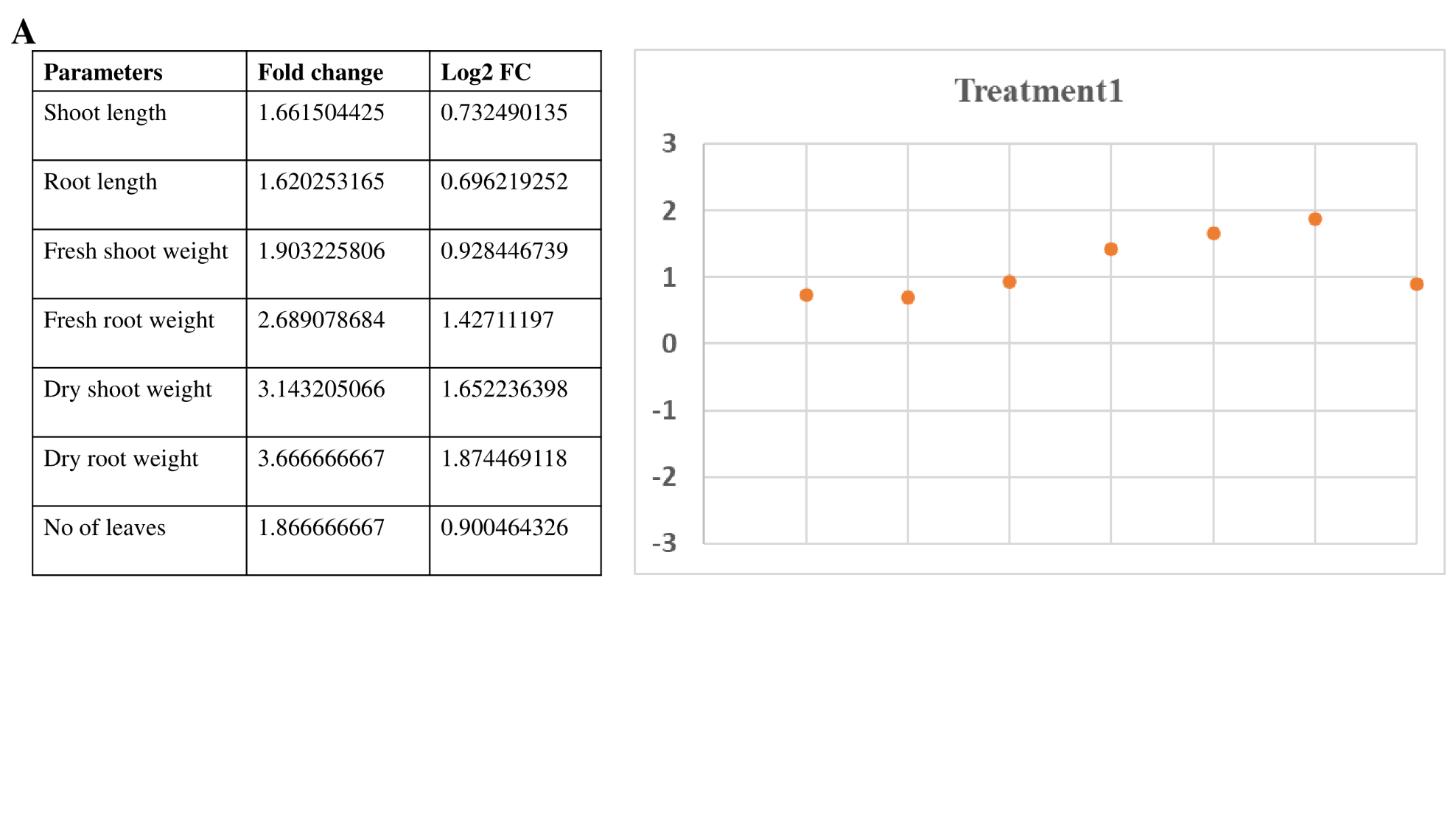


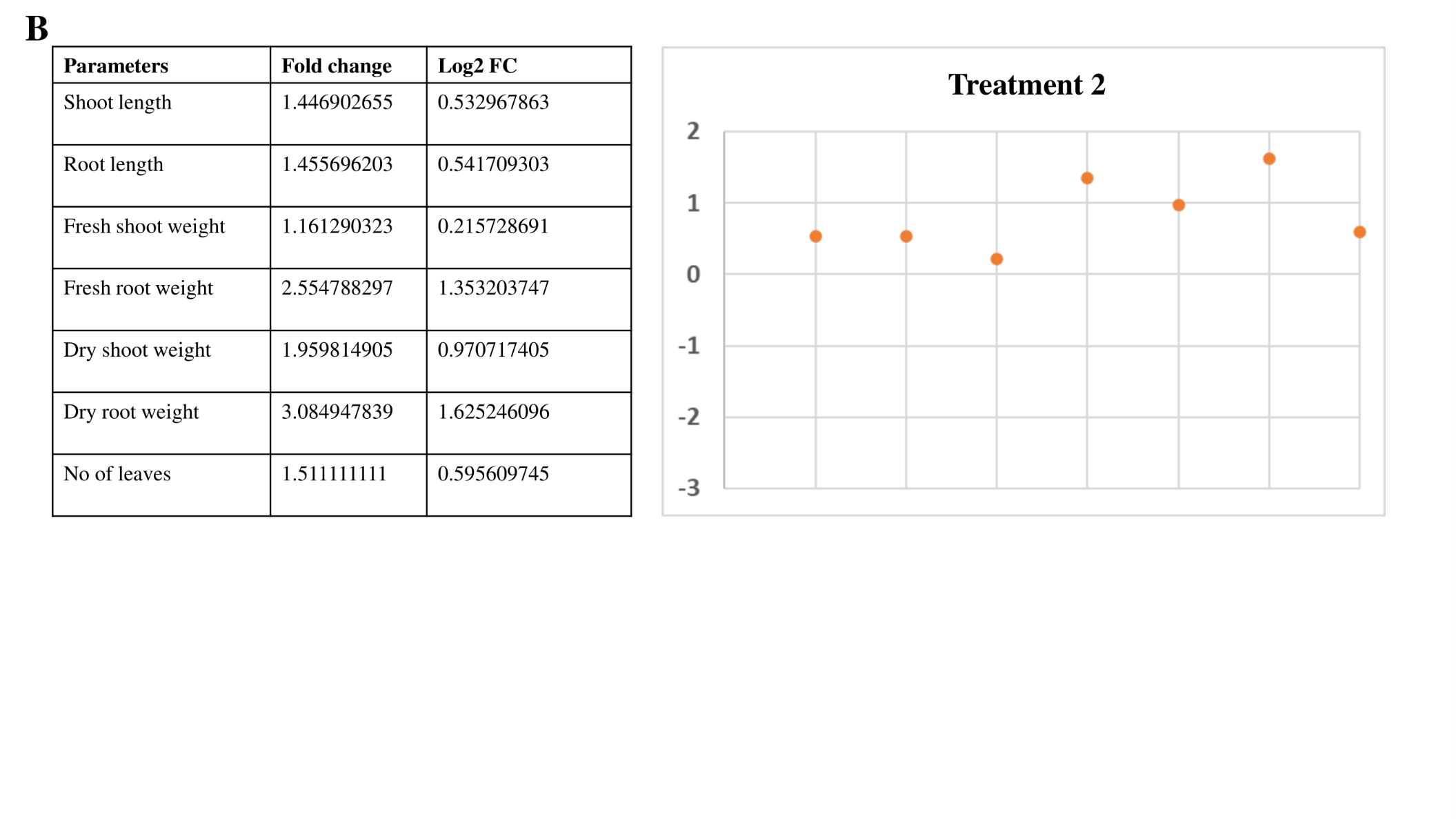


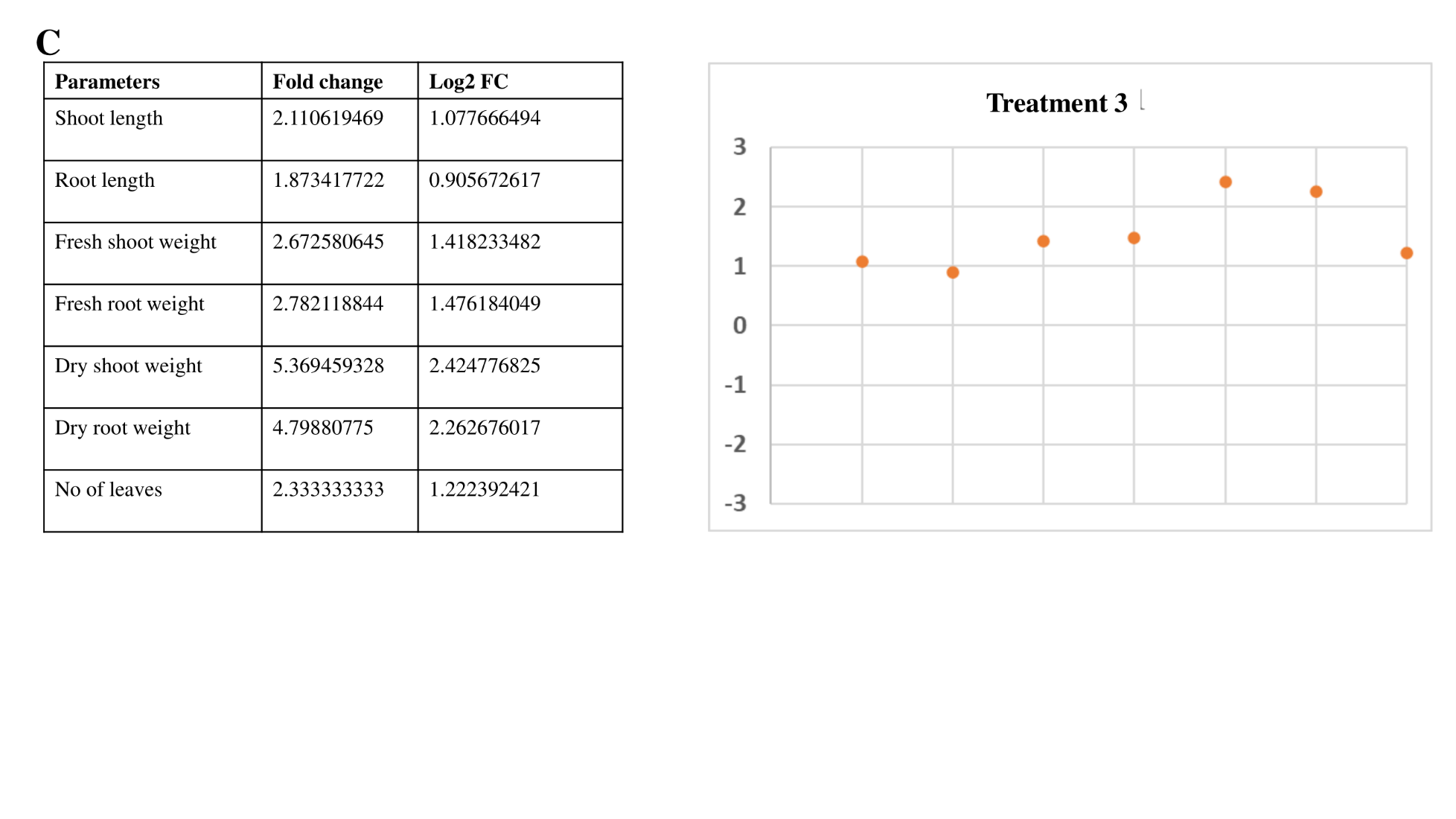


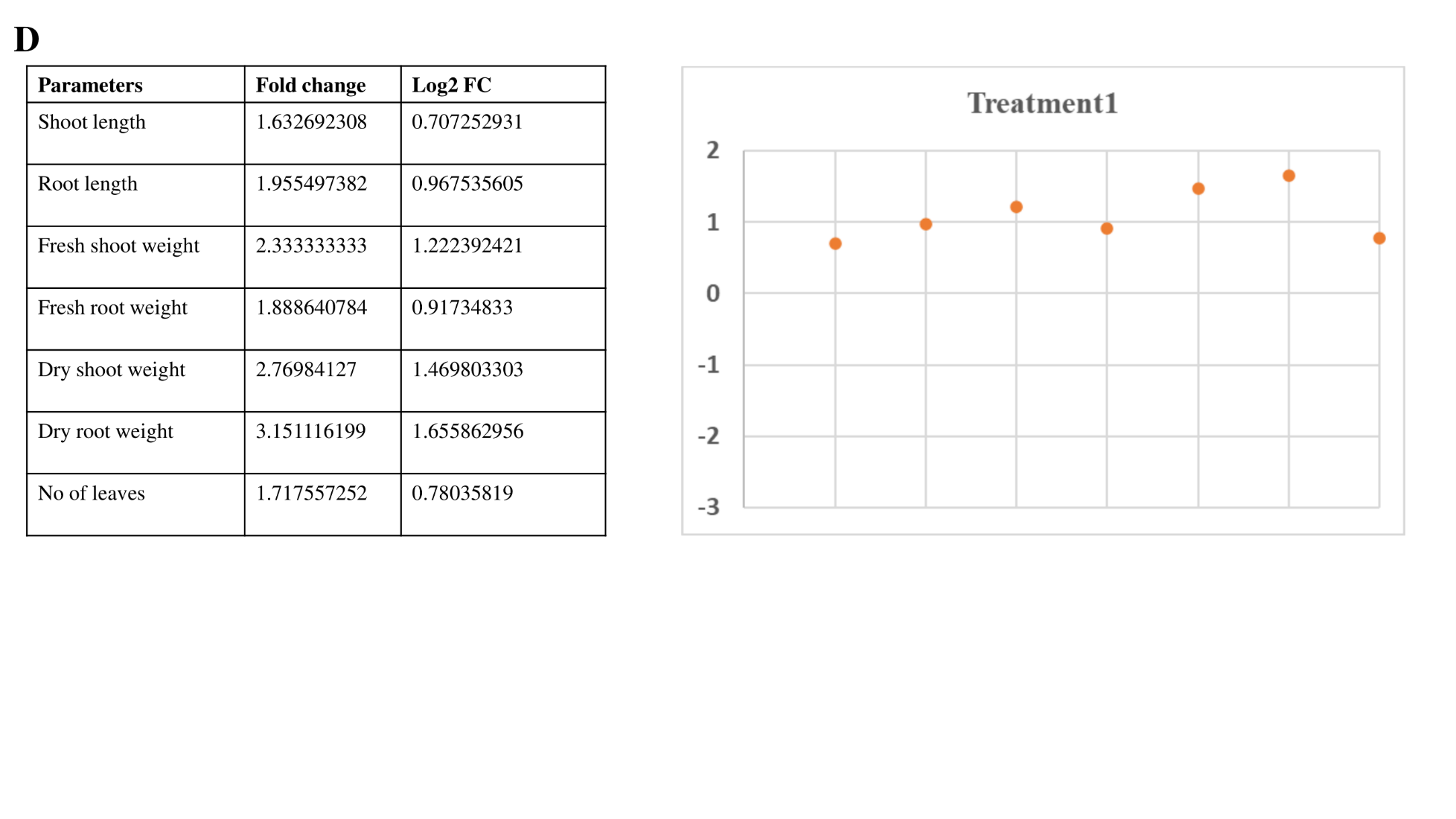


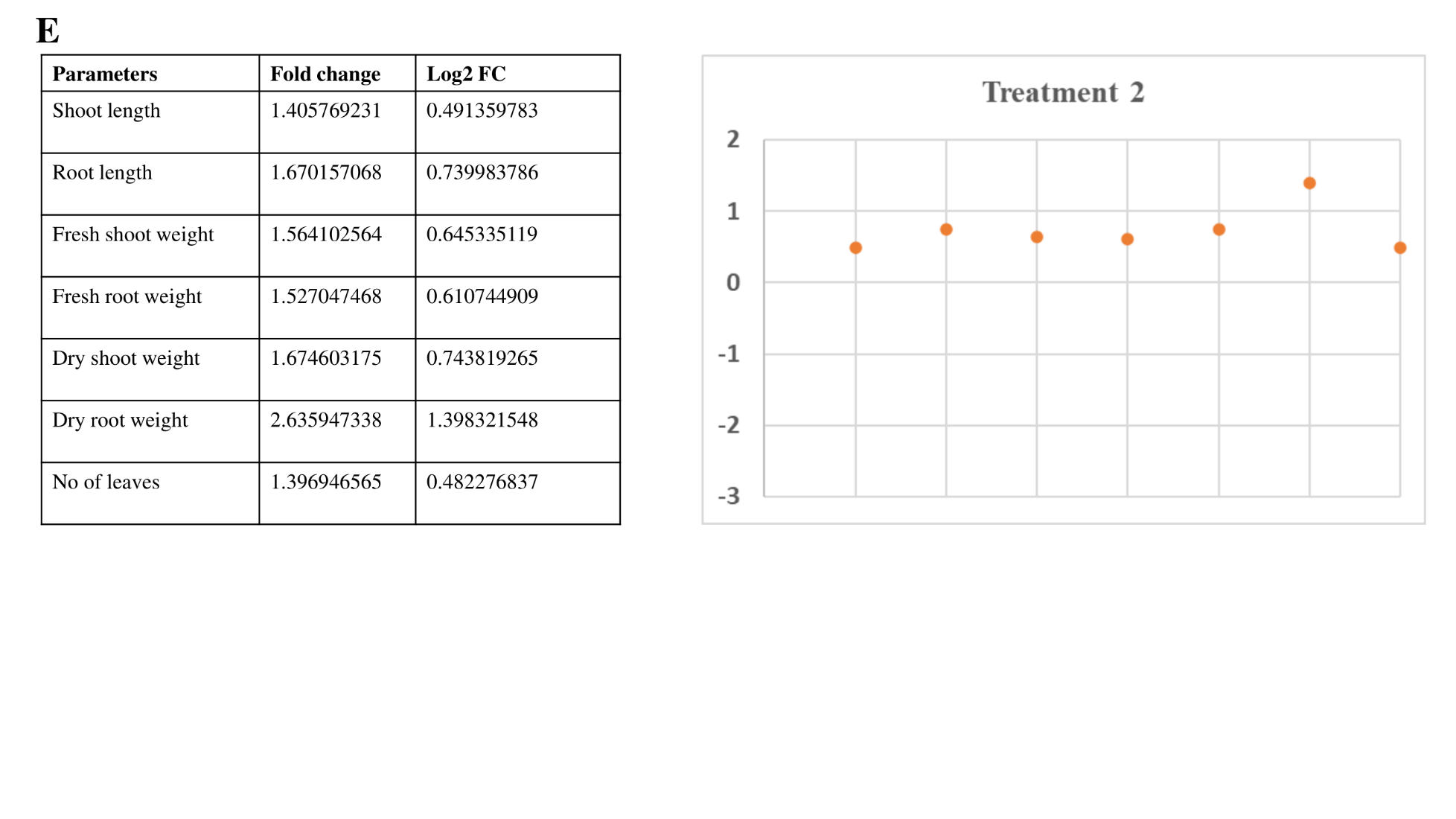


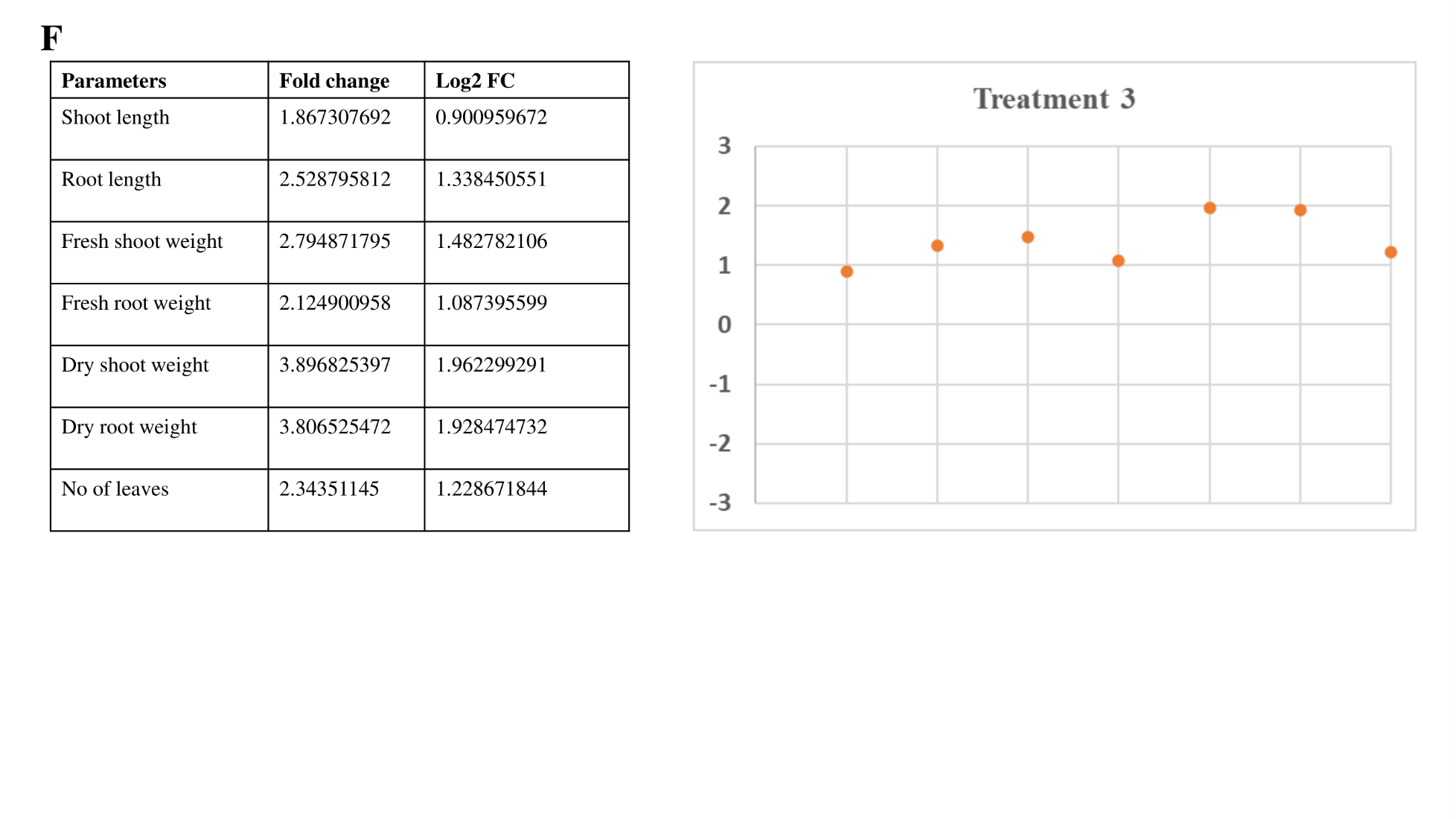


**Supplementary Table1.** Sample collection

| **Sl no.** | **Orchid species** | **Tissue of origin** | **Sampling site and GIS location** |
| --- | --- | --- | --- |
| 1 | *Dendrobium Nobile* | Root, Leaf | The orchid society of eastern Himalaya, regional orchid germplasm conservation and propagation centre, Tinsukia 786156, Assam, India.  27^o^ 38’ 55’N  95^o^31’04’’E |
| 2 | *Dendrobium chrysotoxum* | Root, Leaf |  |
| 3 | *Dendrobium moschatum* | Root, Leaf |  |
| 4 | *Dendrobium densiflorum* | Root, Leaf |  |
| 5 | *Dendrobium fugax* | Root, Leaf |  |
| 6 | *Vanda corulea* | Root, Leaf |  |
| 7 | *Micropera obtusa* | Root, Leaf |  |
| 8 | *Cymbidium eburneum* | Root, Leaf |  |
| 9 | *Renanthera imschootiana* | Root, Leaf |  |
| 10 | *Vanda corulea* | Root, Leaf |  |

**Supplementary Table 2.** Molecular identification of orchid associated endophytic actinobacteria based on 16S rRNA gene sequences.

| **Sl. No** | **Isolate code** | **NCBI Gen Bank accession no** | **Base pair** | **Tophit taxon name with accession no (EzTaxon)** | **Similarity %** | **Species identification** |
| --- | --- | --- | --- | --- | --- | --- |
| 1 | DNRA1 | OM746936 | 1468 | *Streptomyces albiflaviniger AJ391812* | 97.88 | *Streptomyces* sp. |
| 2 | DNRA2 | OM776921 | 1513 | *Nocardiopsis yanglingensis GQ463465* | 99.09 | *Nocardiopsis* sp. |
| 3 | DNAR3 | OM773537 | 1425 | *Nocardiopsis exhalans AY036000* | 98.59 | *Nocardiopsis* sp. |
| 4 | DNRA4 | OM674666 | 1431 | *Streptomyces alba CP103788* | 97.80 | *Streptomyces* sp. |
| 5 | DNRA5 | OM648298 | 1429 | *Streptomyces sioyaensis DQ026654* | 98.60 | *Streptomyces* sp. |
| 6 | DNLA1 | OM773535 | 1422 | *Nocardiopsis yanglingensis GQ463465* | 98.94 | *Nocardiopsis* sp. |
| 7 | DNLA2 | OP313739 | 1444 | *Streptomyces yanii AB006159* | 100 | *Streptomyces* sp |
| 8 | DNLA13 | OM674397 | 1425 | *Streptomyces kronopolitis KP050495* | 99.44 | *Streptomyces* sp. |
| 9 | DCRA1 | OP279764 | 1409 | *Streptomyces kebangsaanensis HM449824* | 99.79 | *Streptomyces* sp. |
| 10 | DCRA2 | MK140996 | 1362 | *Streptomyces griseoflavus AJ781322* | 99.71 | *Streptomyces* sp. |
| 11 | DCRA3 | OM773478 | 1425 | *Streptomyces althioticus AY999791* | 99.08 | *Streptomyces* sp. |
| 12 | DCLA1 | OM674281 | 1425 | *Streptomyces fagopyri MN044908* | 98.63 | *Streptomyces* sp. |
| 13 | DCLA2 | OM674576 | 1410 | *Streptomyces flaviolus AB184786* | 99.08 | *Streptomyces* sp. |
| 14 | DCLA5 | MK140994 | 1363 | *Streptomyces greseoflavus AJ781322* | 100 | *Streptomyces* sp. |
| 15 | DMRA1 | OM772762 | 1432 | *Streptomyces chumphonensis AB738400* | 97.90 | *Streptomyces* sp. |
| 16 | DMRA2 | OP314453 | 1448 | *Pseudonocardia endophytica DQ887489* | 99.79 | *Pseudonocardia* sp. |
| 17 | DMRA3 | OP279737 | 1428 | *Nocardia callitridis FJ805428* | 99.28 | *Nocardia* sp. |
| 18 | DMLA1 | OP313501 | 1423 | *Microbacteroum phyllosphaerae AJ277840* | 99.27 | *Microbacterium* sp. |
| 19 | DMLA8 | OP315317 | 1435 | *Streptomyces microflavus AB184284* | 99.93 | *Streptomyces* sp. |
| 20 | DDRA1 | OM674455 | 1401 | *Pseudonocardia antarctica AJ576010* | 99.36 | *Pseudonocardia* sp. |
| 21 | DDRA2 | OM672679 | 1402 | *Actinomadura nitrigenes AY035999* | 99.79 | *Actinomadura* sp. |
| 22 | DDRA3 | OM680925 | 1422 | *Streptomyces kronopolitis KP050495* | 99.29 | *Streptomyces* sp. |
| 23 | DDRA4 | OP279747 | 1502 | *Streptomyces cyslabdanicus AB915216* | 99.45 | *Streptomyces* sp. |
| 24 | DDLA1 | OM753103 | 1463 | *Streptomyces adustus LC026279* | 100 | *Streptomyces* sp. |
| 25 | DDLA2 | OM735535 | 1481 | *Actinomadura glauciflava AF153881* | 99.51 | *Actinomadura* sp. |
| 26 | DFAR1 | OP265707 | 1459 | *Nocardia ninae DQ235687* | 97.65 | *Nocardia* sp. |
| 27 | DFRA2 | OP313605 | 1454 | *Mycolocibacterium anyangense KJ855063* | 98.71 | *Mycolicibacterium* sp. |
| 28 | DFRA3 | OP279748 | 1524 | *Strptomyces cinnabarigrisus MF536523* | 99.06 | *Streptomyces* sp. |
| 29 | DFLA1 | MK140993 | 1463 | *Streptomyces yokosukanensis KQ948269* | 99.03 | *Streptomyces* sp. |
| 30 | DFLA4 | OP279740 | 1460 | *Nocardia bhagyanarayanae JX076851* | 98.33 | *Nocardia* sp. |
| 31 | CARA1 | OP279749 | 1522 | *Streptomyces polygonate KP208836* | 99.93 | *Streptomyces* sp. |
| 32 | CARA2 | OP313683 | 1381 | *Nocardioides kongjuensis DQ218275* | 97.44 | *Nocardioides* sp. |
| 33 | CARA3 | OP256561 | 1420 | *Actinomadura darangshiensis FN646682* | 99.64 | *Actinomadura* sp. |
| 34 | CALA1 | OM753111 | 1436 | *Streptomyces pratensis JQ806215* | 100 | *Streptomyces* sp. |
| 35 | RRRA1 | OM674459 | 1428 | *Streptomyces chumphonensis AB738400* | 97.75 | *Streptomyces* sp. |
| 36 | RRR46 | ON076552 | 1507 | *Streptomyces staurosporininus FR692111* | 99.23 | *Streptomyces* sp. |
| 37 | RRLA1 | OP314520 | 1443 | *Pseudonocardia nantogensis JQ819252* | 99.79 | *Pseudonocardia* sp |
| 38 | RVRA1 | OP313605 | 1402 | *Nocardia suismassiliense LT984490* | 98.85 | *Nocardia* sp. |
| 39 | RVRA2 | OM648299 | 1418 | *Actinomadura maheshkhaliensis AB331731* | 99.71 | *Actinomadura* sp. |
| 40 | RVRA3 | MH493677 | 1346 | *Streptomyces lydicus KF712383* | 99.48 | *Streptomyces* sp. |
| 41 | RVRA4 | OM746929 | 1432 | *Streptomyces sioyaensis DQ026654* | 98.86 | *Streptomyces* sp. |
| 42 | RVRA6 | OM672245 | 1519 | *Streptomyces cocklensis FR692107* | 98.88 | *Actinomadura* sp. |
| 43 | RVRA7 | OM672243 | 1427 | *Streptomyces argenteolus AB184528* | 99.86 | *Streptomyces* sp. |
| 44 | RVAR8 | OP313686 | 1436 | *Nocardiopsis dassonvillei LR606207* | 99.44 | *Nocardiopsis* sp. |
| 45 | RVLA1 | OP314452 | 1505 | *Streptomyces ardesiacus DQ026631* | 99.72 | *Streptomyces* sp. |
| 46 | MORA11 | OP339857 | 1389 | *Nocardioides endophyticus KC878444* | 99.14 | *Nocardioides* sp. |
| 47 | MOLA1 | MK140995 | 1453 | *Streptomyces olivaceus MT543222* | 99.85 | *Streptomyces* sp. |
| 48 | VCRA1 | OP256850 | 1501 | *Actinomadura bangladeshensis AB331652* | 98.75 | *Actinomadura* sp. |
| 49 | VCRA2 | OP267965 | 1391 | *Streptomyces albofaciens AB045880* | 99.14 | *Streptomyces* sp. |
| 50 | VCLA1 | OP287967 | 1471 | *Nocardioides marinus DQ401093* | 96.65 | *Nocardioides* sp. |
| 51 | VCLA3 | OM674577 | 1427 | *Streptomyces chattanoogensis KM573812* | 99.65 | *Streptomyces* sp. |

**Supplementary Table 3**. Biochemical profiling of actinobacterial isolates VCLA3 and RVRA7 (KB009 Hi Carbohydrate kit, HiMedia).

| **Sl. no** | **Test** | **VCLA3** | **RVRA7** |
| --- | --- | --- | --- |
| 1 | Lactose | + | + |
| 2 | Xylose | - | - |
| 3 | Maltose | + | + |
| 4 | Fructose | + | + |
| 5 | Dextrose | + | + |
| 6 | Galactose | + | + |
| 7 | Raffinose | + | - |
| 8 | Trihalose | + | - |
| 9 | Melibiose | + | + |
| 10 | Sucrose | + | + |
| 11 | L-arabinose | + | + |
| 12 | Mannose | + | + |
| 13 | Inulin | + | + |
| 14 | Sodium gluconate | + | + |
| 15 | Glycerol | + | + |
| 16 | Salicin | - | + |
| 17 | Dulcitol | - | + |
| 18 | Inositol | - | + |
| 19 | Sorbitol | - | + |
| 20 | Mannitol | + | + |
| 21 | Adonitol | + | + |
| 22 | Arabitol | + | + |
| 23 | Erythritol | - | - |
| 24 | α- methyl-D-glucoside | - | - |
| 25 | Rhamnose | + | + |
| 26 | Cellobiose | + | + |
| 27 | Melezitose | - | - |
| 28 | α-methyl-D-Mannoside | - | + |
| 29 | Xylitol | - | + |
| 30 | DNPG | + | + |
| 31 | Esculin hydrolysis | + | + |
| 32 | D- arabinose | + | + |
| 33 | Citrate | + | + |
| 34 | Malonate | + | - |
| 35 | Sorbose | + | - |

**+**, Positive for test; **−**, Negative for test
